# Developing Red and Near-Infrared Delayed Fluorescence Emission in Nitrogen-Substituted Donor–Acceptor Polycyclic Hydrocarbon OLED Emitters: A Theoretical Study

**DOI:** 10.1021/acs.jpca.4c07345

**Published:** 2025-02-26

**Authors:** Smruti Ranjan Sahoo, Glib V. Baryshnikov, Hans Ågren

**Affiliations:** † Division of X-ray Photon Science, Department of Physics and Astronomy, Uppsala University, Box 516, SE-751 20 Uppsala, Sweden; ‡ Laboratory of Organic Electronics, Department of Science and Technology, Linköping University, SE-601 74 Norrköping, Sweden

## Abstract

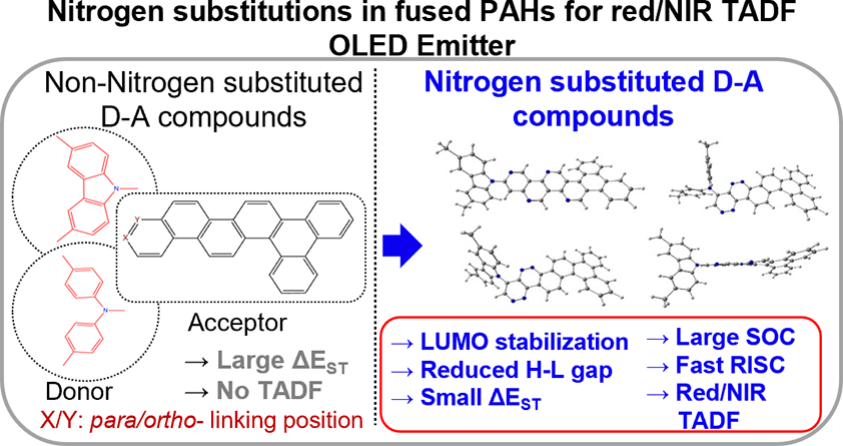

Nitrogen substitutions have shown a great impact for the development of thermally activated delayed fluorescence (TADF)-based organic light-emitting diode (OLED) materials. In particular, much focus has been devoted to nitrogen-substituted polycyclic aromatic hydrocarbons (PAHs) for TADF emitters. In this context, we provide here a molecular design approach for symmetric nitrogen substitutions in fused benzene ring PAHs based on the dibenzo[*a*,*c*]picene (DBP) molecule. We designed possible donor–acceptor (D–A) compounds with dimethylcarbazole (DMCz) and dimethyldiphenylamine (DMDPA) donors and studied the structure and photophysics of the designed D–A compounds. The twisted and extended D–A-type PAH emitters demonstrate red and near-infrared (NIR) TADF emission. Nitrogen substitutions lead to significant LUMO stabilization and reduced HOMO–LUMO energy gaps as well. Additionally, we computed significantly smaller singlet–triplet energy splittings (Δ*E*_ST_) in comparison to non-nitrogen-substituted compounds. The investigated *ortho*-linked D–A compounds show relatively large donor–acceptor twisting separation and small Δ*E*_ST_ compared to their *para*-linked counterparts. For higher number nitrogen (4N)-substituted emitters, we predict small adiabatic Δ*E*_ST_ (Δ*E*_ST_^adia^) in the range 0.01–0.13 eV, and with the *tert*-butylated donors, we even obtained Δ*E*_ST_^adia^ values as small as 0.007 eV. Computed spin–orbit coupling (SOC) for the T_1_ triplet state on the order of 0.12–2.28 cm^–1^ suggests significant repopulation of singlet charge transfer (^1^CT) excitons from the triplet CT and locally excited (^3^CT+LE) states. Importantly, the small Δ*E*_ST_^adia^ and large SOC values induce a reverse intersystem crossing (RISC) rate as high as 1 × 10^6^ s^–1^, which will cause red and NIR delayed fluorescence in the 4N-substituted D–A emitters. Notably, we predict red TADF emission for the *para*-linked compound **B4** at 670 nm and the *ortho*-linked compound **D4** at 713 nm and delayed NIR emission at 987 and 1217 nm for the *ortho*-linked compounds **D3** and **E3**, respectively.

## I Introduction

Since the pioneering work by Adachi in 2012, the third-generation thermally activated delayed fluorescence (TADF)-based organic emitters have increasingly received wide attention in organic light emitting diode (OLED) industries because of their high efficiency, stability, and color purity and because they could replace the current state-of-the-art phosphorescent light-emitting materials.^1−4^ The TADF materials have the potential to produce dual fluorescence emission: one is 25% singlet exciton emission in terms of prompt fluorescence, and the other one is the delayed emission originating from the upconversion of 75% triplet excitons through reverse intersystem crossing (RISC) from relevant triplet (T_*n*_, *n* = 1, 2, 3, etc.) states to the singlet S_1_ state.^1,5,6^ The mechanism of harvesting both singlet and triplet excitons and the possibility of achieving 100% internal quantum efficiency (IQE) have made them attractive in current OLED development.^6,7^

Efficient RISC processes in TADF materials are characterized by a small singlet–triplet energy splitting (Δ*E*_ST_) and a large spin–orbit coupling (SOC) between the lowest singlet state and the relevant triplet state.^1,5,8^ In donor–acceptor (D–A)-type molecular TADF emitters, the effective twisting in the D–A framework leads to significant spatial separation of the highest occupied molecular orbital (HOMO) and lowest unoccupied molecular orbital (LUMO), resulting in a small Δ*E*_ST_.^5^ The forbidden spin-flip process between singlet (^1^CT) and triplet (^3^CT) charge-transfer states is generally due to the small SOC values in organic compounds, whereas the coupling between the triplet locally excited (^3^LE) or ^3^CT+LE states and ^1^CT states of different spatial localization gives rise to large SOC matrix elements, which then induces spin flip and subsequently an efficient RISC mechanism.^1,8^

Polycyclic aromatic hydrocarbons (PAHs) such as linearly arranged acenes, helically shaped helicenes, and the fused benzene ring zigzag array-based phenancene family and their derivatives have gathered great interest as organic electronic materials for organic field-effect transistors (OFETs) as well as for OLEDs.^9−17^ Nitrogen atom substitutions have been shown to greatly influence the semiconducting properties of the organic materials^16,18^ and have been used in the design of PAH fluorescence emitters,^10^ TADF-based OLED materials,^17,19,20^ and multiple-resonance-type TADF materials based on heteroatom-doped PAHs.^21,22^ Recently, You et al. successfully reported the “nitrogen effect” on the performance of TADF-sensitized fluorescence for nitrogen-doped PAHs and fabricated an imine-embedded PAH (IE-PAH)-type blue OLED with 32.7% external quantum efficiency (EQE).^10^ Nitrogen-substituted PAHs have been seen as highly promising in TADF and OLEDs because of their low- lying LUMO acceptors in D–A-type compounds. In this regard, Zysman-Colman and his group^19^ reported four orange-to-red TADF D–A compounds with 11-(9,9-dimethylacridin-10(9*H*)-yl) (DMA) as the donor and nitrogen-doped dibenzo[*a*,*c*]phenazine (DBPZ) as the acceptor and found that an increase of the number of nitrogen atoms in the acceptor led to a stabilized LUMO, a smaller Δ*E*_ST_, and faster RISC. The report by Lindner, Data, and Kubas showed the development of nitrogen-doped PAHs with a D–A–D framework (with the twisted donor and bulky *O*-alkyl chain at peripheries) for efficient TADF emission with a small Δ*E*_ST_ of 0.04–0.28 eV and an excellent solid-state photoluminescence quantum yield (PLQY) of 96% with yellow or orange-red emission as well as 21.9% maximum EQE.^23^ Similarly, Xu et al.^24^ developed a T-shaped D–A–D-type TADF molecule with two triphenylamine (TPA) units as donors (at both *ortho* and *para* positions of the acceptor) and planar dipyridophenazine (DPPZ) as the acceptor (with four nitrogen atoms). In that report, the efficient RISC and high radiative decay constant resulted in a PLQY of 87%, and the bilayer OLED with *p*TPA-DPPZ as an emissive layer produced deep-red electroluminescence (EL) with excellent color purity, achieving an EQE of 12.3%. With the triazatruxene (TAT) as the donor and DBPZ as the acceptor, Tong and Wang developed novel TADF emitters which showed high solid-state PLQY because of their rigid and large planar conjugated structures.^25^ Large steric hindrance of the structures showed here an effective TADF mechanism with a small Δ*E*_ST_, and the solution-processable OLED device based on TAT-DBPZ displayed a red EL peak at 604 nm and 15.4% EQE. Additionally, a corresponding fluorine-substituted acceptor (FDBPZ)-based OLED device showed red-shifted EL emission at 611 nm with an EQE of 9.2%.

Hence, nitrogen-substituted PAH-based D–A-type compounds have been successfully demonstrated as orange-red TADF emitters.^19,23−26^ In the PAH family, fused-benzene-ring aromatic moieties are found to be favorable for extended electron delocalization and narrower energy gap and therefore are suitable for near-infrared (NIR) emission.^27−30^ Herein we considered dibenzo[*a*,*c*]picene (DBP), a PAH with seven fused benzene rings, as the electron-accepting molecule, which in earlier studies by Yamaji et al.^15^ showed visible fluorescence and phosphorescence emission with 15% fluorescence quantum yield in nonpolar cyclohexane. Based on its photophysical properties, extended conjugation, and twisted molecular geometry, we tried to rationally design D–A-type compounds by considering nitrogen-substituted DBPs as acceptors and dimethylcarbazole (DMCz) and dimethyldiphenylamine (DMDPA) as donors. We carried out a systematic theoretical investigation on molecular design, structure–property relationship, and photophysical properties based on calculations within the density functional theory (DFT) framework. In comparison to previous studies on nitrogen substitutions for orange-red emission, our theoretical report on these larger seven-ring systems with nitrogen-substituted DBP-based D–A compounds predicts red/NIR delayed fluorescence emission and TADF OLED emission.

**Scheme 1:**
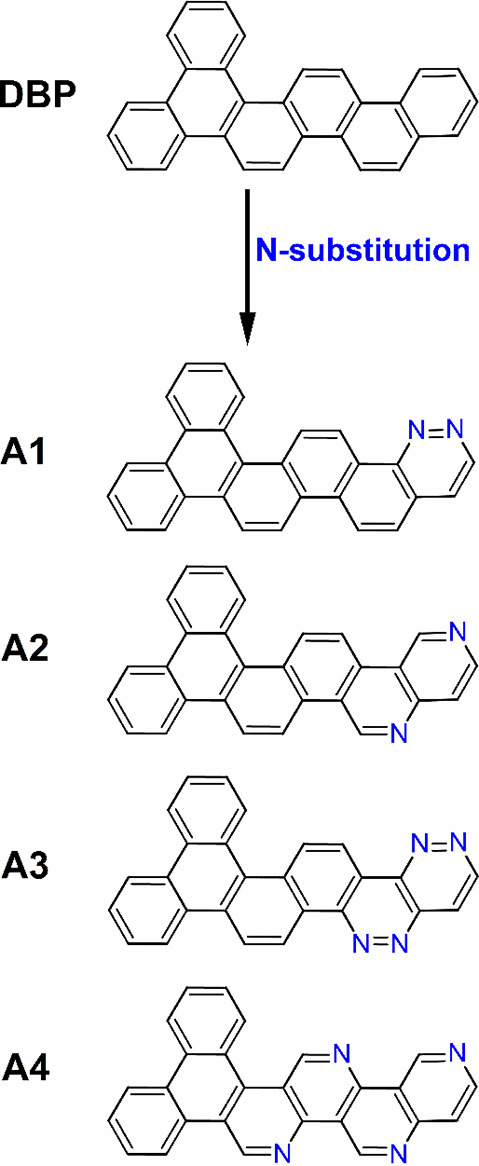
Chemical Structures and Molecular Design of Nitrogen-Substituted DBP Compounds **A1**–**A4**

The development of red and NIR TADF emitters has relied on several factors, like increase in molecular rigidity, significant twisting separation between donors and acceptors, strong electron-donation and electron-accepting properties, and LUMO stabilization.^4,31,32^ Herein we report the effect of nitrogen functionalization for creating efficient red and NIR thermally activated delayed emission in D–A-type organic emitters and exemplify the effect by studying symmetric nitrogen functionalization in PAHs with seven fused benzene rings. The design principle is presented in Scheme 1 and described in Section II. We have considered strong electron donors such as DMCz and DMDPA for D–A emitters and also investigated the effect of their linking position to the acceptor (*ortho* or *para* linkage) and the photophysical properties of the studied emitters. To the best of our knowledge, there is no report available on studies of the effects of symmetric nitrogen functionalization on the DBP acceptor and the linking position of the donor molecule on the TADF emission properties of these D–A-type organic emitters. We designed two types of D–A-type emitters: one type is with nitrogen-substituted DBP acceptor-based emitters (16 compounds), and the other type is without nitrogen substitutions (four compounds). Eventually, we found that among the designed compounds, the D–A emitters substituted with a higher number of nitrogens (4N) exhibited a smaller adiabatic Δ*E*_ST_ (Δ*E*_ST_^adia^) in the range 0.01–0.13 eV and higher triplet (T_1_) → singlet (S_1_) RISC rate constants (*k*_RISC_) on the order of 10^6^ s^–1^. This resulted in red and NIR delayed fluorescence emission in nonpolar cyclohexane solvent at room temperature. In addition, these properties have been characterized for both groups of the investigated *ortho*- and *para*-linked D–A emitters. Furthermore, we considered stronger donors such as *tert*-butylated DMCz and DMDPA (hence called DTBCz and DTBDPA) along with the 4N-substituted DBP acceptor, and as anticipated, we could predict an efficient TADF process with narrower Δ*E*_ST_^adia^, in fact in the range 0.007–0.06 eV.

## II Molecular Design and Theoretical Methods

**Figure 1:**
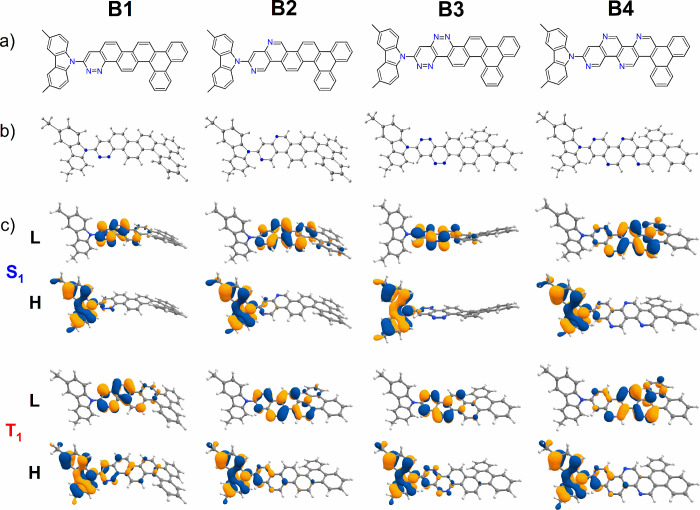
Chemical structures from theoretical calculations on compounds **B1**–**B4**. (a) Chemical structures of **B1**–**B4**. (b) B3LYP/6-31+G(d)-optimized ground-state (S_0_) structures. (c) Calculated HOMO (H) and LUMO (L) charge density distribution plots for the excited states S_1_ and T_1_ obtained using the TDDFT/B3LYP/6-31+G(d) method in cyclohexane solution state.

**Figure 2:**
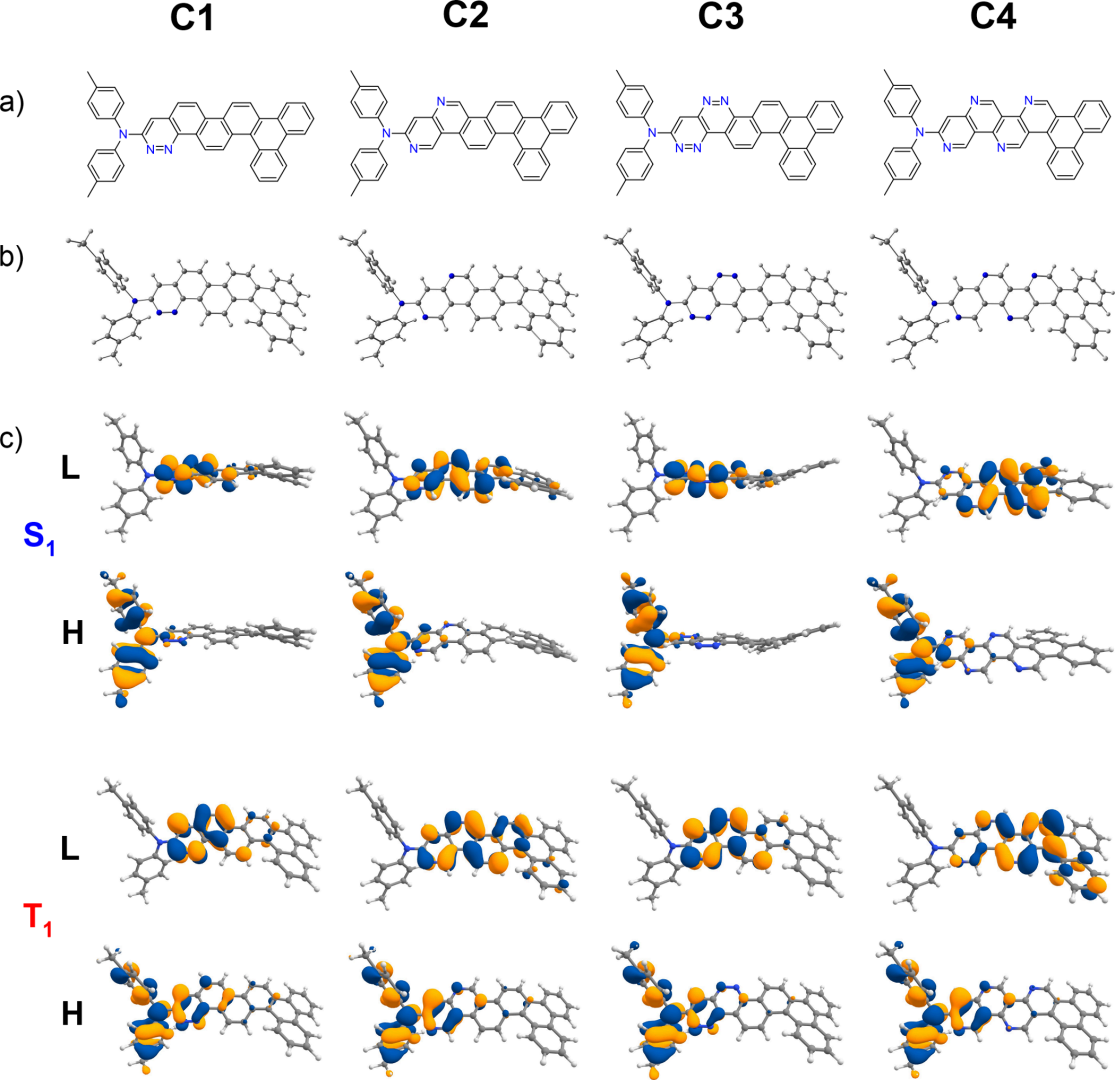
Chemical structures from theoretical calculations on compounds **C1**–**C4**. (a) Chemical structures of **C1**–**C4**. (b) B3LYP/6-31+G(d)-optimized ground-state (S_0_) structures. (c) Calculated HOMO (H) and LUMO (L) charge density distribution plots for the excited states S_1_ and T_1_ obtained using the TDDFT/B3LYP/6-31+G(d) method in cyclohexane solution state.

**Figure 3:**
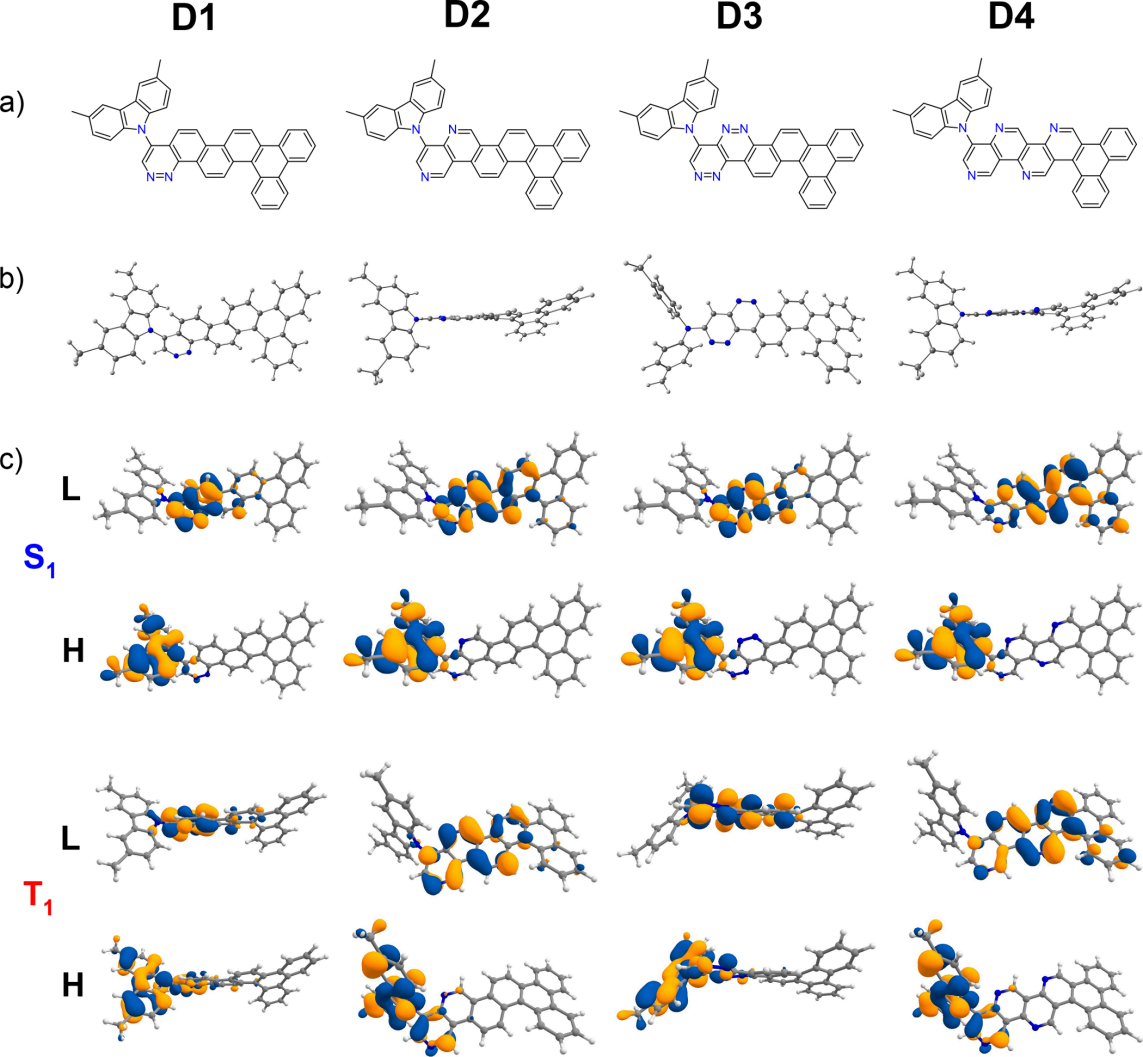
Chemical structures from theoretical calculations on compounds **D1**–**D4**. (a) Chemical structures of **D1**–**D4**. (b) B3LYP/6-31+G(d)-optimized ground-state (S_0_) structures. (c) Calculated HOMO (H) and LUMO (L) charge density distribution plots for the excited states S_1_ and T_1_ obtained using the TDDFT/B3LYP/6-31+G(d) method in cyclohexane solution state.

**Figure 4:**
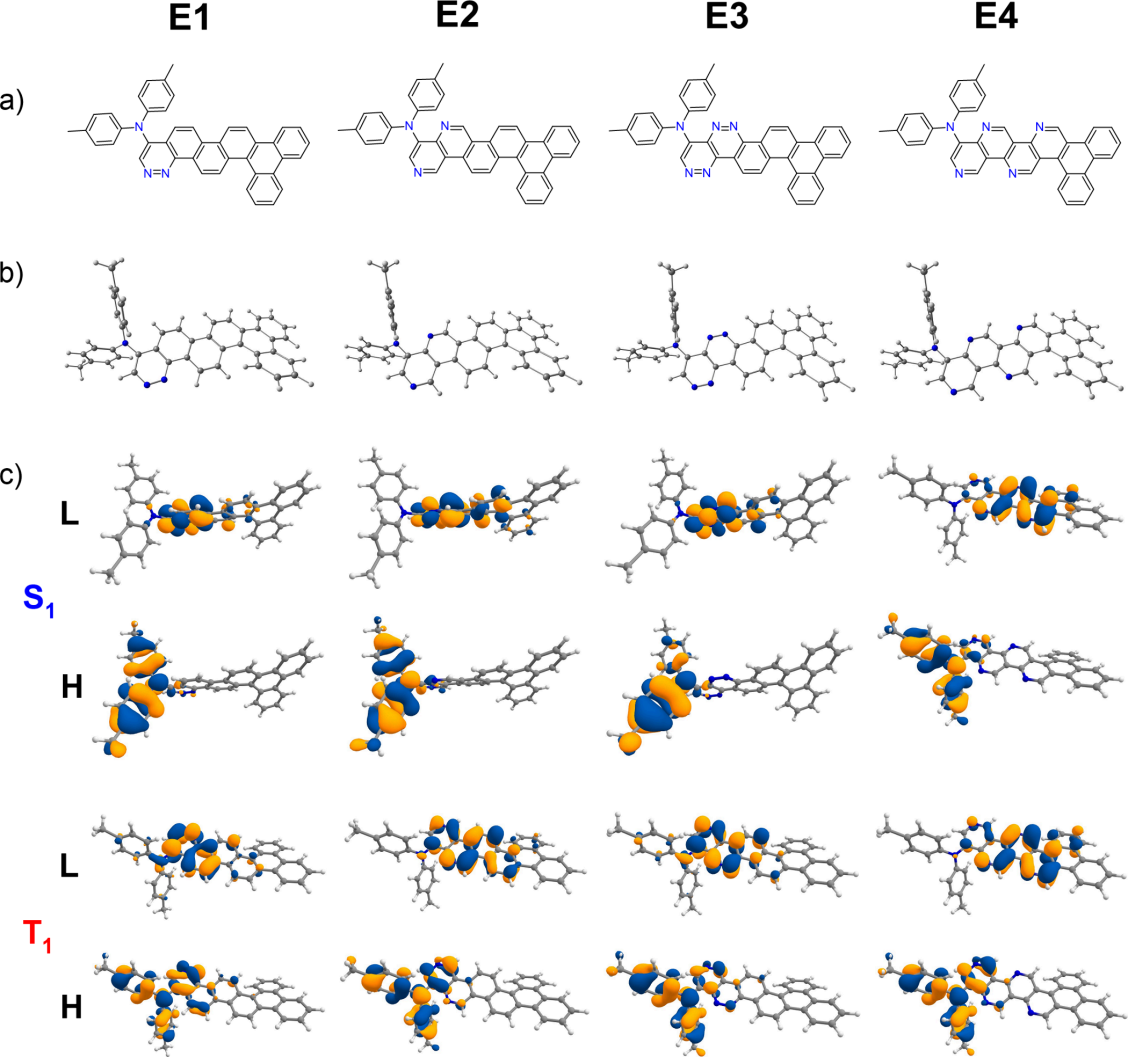
Chemical structures from theoretical calculations on compounds **E1**–**E4**. (a) Chemical structures of **E1**–**E4**. (b) B3LYP/6-31+G(d)-optimized ground-state (S_0_) structures. (c) Calculated HOMO (H) and LUMO (L) charge density distribution plots for the excited states S_1_ and T_1_ obtained using the TDDFT/B3LYP/6-31+G(d) method in cyclohexane solution state.

The X-ray crystallographic structure CCDC 1521686, obtained from the Cambridge Crystallographic Data Center, was chosen as the initial structure of DBP.^15^ We introduced nitrogen (N) atoms into the parent DBP molecule backbone to design an N-rich electron acceptor. Though there are several possibilities of substitutions, herein we focused on the symmetric substitution of the N atoms (i.e., 2N and 4N) depending on the number of fused benzenes in the picene unit, to study the structure–property relationships of the DBPs. Additionally, depending upon the structure, we considered the *ortho* and *meta* substitutions of the N atoms in the benzene ring. Eventually, we obtained two 2N-substituted (**A1**, **A2**) and two 4N-substituted (**A3**, **A4**) DBP compounds (see Scheme 1). The effect of nitrogen functionalization on the molecular structures and electronic and photophysical properties has been analyzed in detail. To design D–A-type red and NIR TADF emitters we investigated strong electron donors like DMCz and DMDPA (where the latter possesses greater donor strength) along with the N-rich highly conjugated DBP electron acceptors **A1**–**A4**. We followed two types of linking (viz., *para* and *ortho*) of the electron donors to design the possible 16 D–A structures. Henceforth, we denote the designed *para*-linked D–A-type compounds with DMCz as the donor, namely, *p*-DMCz–**A1**, *p*-DMCz–**A2**, *p*-DMCz–**A3**, and *p*-DMCz–**A4**, as **B1**, **B2**, **B3**, and **B4**, respectively. Those with DMDPA as the donor, namely, *p*-DMDPA–**A1**, *p*-DMDPA–**A2**, *p*-DMDPA–**A3**, and *p*-DMDPA–**A4**, are denoted as **C1**, **C2**, **C3**, and **C4**, respectively. The detailed structures of these designed D–A compounds are shown in Figures 1 and 2. Similarly, in the case of *ortho*-linked D–A-type compounds, we denote *o*-DMCz–**A1**, *o*-DMCz–**A2**, *o*-DMCz–**A3**, and *o*-DMCz–**A4** as **D1**, **D2**, **D3**, and **D4**, respectively, and *o*-DMDPA–**A1**, *o*-DMDPA–**A2**, *o*-DMDPA–**A3**, and *o*-DMDPA–**A4** as **E1**, **E2**, **E3**, and **E4**, respectively (see Figures 3 and 4).

We first employed the B3LYP hybrid functional^33^ and the 6-31+G(d) basis set^34−36^ for ground-state (S_0_) geometry optimization and frequency analysis for the DBP molecule within the DFT formalism.^33,37,38^ This methodology has been successfully applied for the DFT calculations of related helicenes.^15^ The optimized geometry was then considered as an initial guess where nitrogen substitutions were carried out. The resulting N-rich DBP compounds (**A1**–**A4**) and the D–A compounds **B1**–**B4**, **C1**–**C4**, **D1**–**D4**, and **E1**–**E4** were optimized through ground-state geometry optimization and vibrational analysis at the same level of theory. We employed the same B3LYP functional with the 6-31+G(d) basis set for the excited-state structure calculations. The singlet excited state S_1_ geometry relaxation and Hessian were obtained at the time-dependent density functional theory (TDDFT) level, while the triplet state T_1_ geometries were computed by using the spin-unrestricted B3LYP/6-31+G(d) methodology. Additionally, relevant adiabatic and vertical excitation energy calculations were carried out at the TDDFT/B3LYP/6-31G+(d) level of theory. For a reliable description of the photophysical properties for the designed D–A structures, the polarizable continuum solvation model (PCM)^40−42^ in the nonpolar cyclohexane^15^ solvent environment was employed. All the above calculations were performed using the Gaussian 16 package.^43^

It should be noted that for an improved description of the singlet–triplet splitting energy, herein we considered the adiabatic excited energy for the S_1_ and T_1_ states and the singlet–triplet energy gaps between them (Δ*E*_ST_^adia^)^8^ rather than vertical singlet–triplet gap energies (though this is successfully considered for many studies).

The perturbative spin–orbit coupling (pSOC) calculations were performed at the TD-DFT level. Within the scalar relativistic (SR) approximation, we used the triple-ζ polarized (TZP) basis set^44^ with no frozen core and the B3LYP functional to obtain the perturbative excitation energies which include SOC effects. Additionally, the conductor-like screening model (COSMO)^45^ continuum solvation approach was considered to analyze the matrix effects. Here, the SOC matrix elements were calculated as the root sum square, i.e., the square root of the sum of squares of SOC matrix elements of all sublevels of the unoccupied states:^8,46^
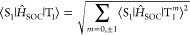
where *m* is the relevant triplet-state sublevel. Here it is notable that the singlet S_1_ state geometries were considered for the direct intersystem crossing (ISC) rate calculations, whereas the RISC rate calculations were carried out at the T_1_ state geometry for all studied compounds (this has been recommend in previous studies).^8^ The pSOC calculations were performed using the Amsterdam Density Functional (ADF) 2023 package.^47^

We implemented the Fermi golden rule to compute direct and reverse ISC rate constants:^8,48−50^
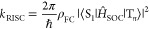
1where the second factor has been defined before and the first factor ρ_FC_ is the Franck–Condon-weighted density of states, which can be calculated using Marcus theory as
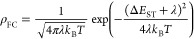
2where λ denotes the reorganization energy. For the RISC rate, λ can be calculated as the difference between the energy of the S_1_ state at the T_1_ state geometry and the energy of the S_1_ state at the S_1_ geometry, i.e., λ = *E*_S_1_/T_1__ – *E*_S_1_/S_1__. Similarly, to compute the ISC rate, λ is the difference between the energy of the T_1_ state at the S_1_ state geometry and the energy of the T_1_ state at the T_1_ geometry, i.e., λ = *E*_T_1_/S_1__ – *E*_T_1_/T_1__. Here Δ*E*_ST_ < 0 for the ISC rate, and it has the same magnitude as the RISC rate with the opposite sign, i.e., Δ*E*_ST_ > 0.

To calculate the radiative fluorescence decay rates, we used the Strickler–Berg equation:^51,52^*k*_r_ = (1/1.5)*fE*_S_1_→S_0__, where *f* is the intensity of the transition (calculated oscillator strength value) and *E*_S_1_→S_0__ is the de-excitation energy.

## III Results and Discussion

### A Structural Confirmation

Extended conjugation of acceptors contributes to stabilization of the LUMO and reduction of the electronic HOMO–LUMO energy gap as well as the singlet and triplet energies of the emitters. Additionally, an increase of N-functionalization leads to LUMO stabilization and increases the electron-accepting character of the acceptor molecule.^4^ These factors help in developing red and NIR TADF emitters with a combination of strong donors and acceptors. Initially, to design stabilized extended conjugated heteroatomic acceptor structures, we performed a symmetrical substitution of N atoms in the DBP molecule—the compounds are shown in Scheme 1. Subsequently, the strong electron donors DMCz and DMDPA were considered to form D–A-type compounds along with our designed N-substituted DBP acceptors with the aim of designing red- and NIR-based TADF emitters. The molecular design architectures of the possible *para*- and *ortho*-linked D–A compounds are given in Figures 1and 2 and Figures 3 and 4, respectively. These two types of linkage have earlier been found to be favorable for designing D–A-type organic emitters.^4,17,24,25,53^

**Figure 5:**
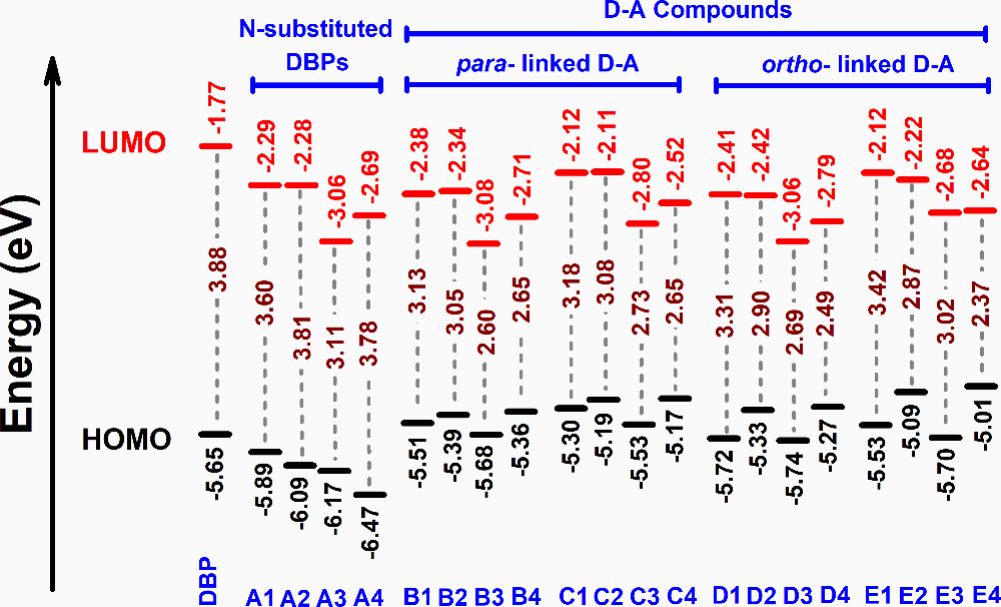
Plots of the calculated HOMO/LUMO energy levels and HOMO–LUMO gaps of the studied compounds.

The optimized geometry of DBP presented a close resemblance with the X-ray crystallographic structure; we did not observe any significant changes in geometrical parameters. The twisting angle between the benzene and picene units (i.e., at nonplanarity) varied only in the range of 0.58–0.75°. Nitrogen substitutions in DBP also do not show significant distortion in molecular geometry. For example, as shown in Figure S1, with respect to DBP the twisting angle in the designed **A1**, **A2**, and **A3** compounds increased by about 0.22°, 0.12°, and 0.35° respectively, and for **A4** we observed a decrease by about 1.69–2.45°. It is notable that N substitutions result in HOMO and LUMO stabilization as well as reduction in the HOMO–LUMO energy gap, and that this is more significant with the increase in the number of nitrogen atoms. To give an example, the HOMO/LUMO energies and HOMO–LUMO gap for DBP are −5.65/–1.77 and 3.88 eV, respectively, and for compound **A4** these are calculated to be −6.47/–2.69 and 3.78 eV, respectively. This increases the conjugation and electron-accepting characteristics of N-substituted DBPs **A1**–**A4**. The electronic energies with calculated HOMO–LUMO gaps of the studied compounds are listed in Figure 5. Further, the HOMOs of DBP and **A1**–**A4** are delocalized over the whole structure, while the LUMOs of **A1**–**A4** are mostly located on the N-substituted fused benzene unit of picene (whereas it was located on the whole of DBP; see Figure S1). This suggests the idea of designing the donor–acceptor architectures by the inclusion of donors in the **A1**–**A4** acceptor unit (see Figures 1–4). Herein, we followed two types of D–A molecular design strategies, i.e., *para* and *ortho* donor linking to the fused edge N-substituted benzene ring of the acceptor unit, and investigated the structure–property relationships. Linking of the donor to the tail position of the N-substituted DBP acceptor could possibly be synthesized in a head-to-tail crystallographic arrangement like the parent DBP molecule.^15^

In the case of the D–A compounds, in general we observed increased HOMO and slightly lowered LUMO energy levels in comparison to nitrogen-substituted the DBPs. Simultaneously, this is followed by a reduction in the HOMO–LUMO energy gaps (Figure 5). For instance, the *para*-linked **B4** is defined by HOMO/LUMO energies of −5.36/–2.71 eV and a HOMO–LUMO gap of 2.65 eV, and the *ortho*-linked **D4** is defined by the values −5.27/–2.79 and 2.49 eV, respectively. We noticed similar higher HOMO levels for the *para*-linked compounds **C1**–**C4** and *ortho*-linked compounds **E1**–**E4**, and also, their LUMO levels were found to be narrowly raised compared to those of the N-substituted DBPs. Compounds such as **B3**, **C3**, **D3**, and **E3** are characterized by lower HOMO and LUMO levels. Herein, reduction in HOMO–LUMO gaps and alignment of HOMO/LUMO energy levels suggested enhancement in donor–acceptor intermolecular interactions and, in particular, more intermolecular charge transfer (CT) interactions for the compounds with a higher number of nitrogens (4N), such as **B3**/**B4**, **C3**/**C4**, **D3**/**D4**, and **E3**/**E4**.

We observed a significant twisting angle between the donor and acceptor units in both the *para* and *ortho* linkages, suggesting a reduction in HOMO and LUMO electron overlap that results in a low Δ*E*_ST_. The results of the quantum-chemistry calculations for twisting angles are provided in Table S1, and the calculated HOMO/LUMO charge density distributions are shown in Figures 1–4.

We found that with the GD3 correction,^54^ no significant change in molecular geometries was predicted as well as for the electronic properties. For example, in 2N-substituted compounds like **B1**, **C1**, **D1**, and **E1**, the GD3-corrected dihedral angle between the donor and acceptor moieties showed a change in the range only 0.14–5.9° at the S_0_ state as compared to the non-GD3 calculations (see Tables S1 and S18). Similarly, for higher-nitrogen-substituted compounds such as **B4**, **C4**, **D4**, and **E4**, the change was found to be 0.8–8.7°. Additionally, within the GD3 correction method, we computed very similar HOMO/LUMO energy levels and HOMO–LUMO energy gaps (Table S19) as those of non-GD3 values (Figure 5).

It can be noted that the DMCz donor unit was more twisted in cases of *ortho*-linked D–A compounds, which is due to the steric hindrance. For instance, at the S_0_ geometry, the *para*-linked **B3** and **B4** possess D–A twisting angles of −40.185°/–36.091° and −40.405°/–35.578°, whereas those for *ortho*-linked **D3** and **D4** are −56.418°/–46.756° and −67.920°/–68.952°, respectively. Similarly, at the S_1_ geometry, these angles are calculated to be −70.220°/–72.739° and −55.754°/–57.586° for **B3** and **B4** and 88.288°/91.584° and 90.414°/93.684° for **D3** and **D4**. The larger twisting angles lead to a relatively lower Δ*E*_ST_ for the *ortho*-linked compounds **D3** and **D4**. In fact, we noticed about 0.1 eV or even smaller Δ*E*_ST_ (see Tables 1–4) for both the pairs **B3**/**B4** and **D3**/**D4**, which would empower the delayed fluorescence emission in these compounds. The DMDPA-donor-based compounds studied herein provide a more twisted confirmation due to the presence of the unfused benzene group in the donor, and we also calculated a lower Δ*E*_ST_ for both the *para*-linked pair **C3**/**C4** and the *ortho*-linked pair **E3**/**E4** (see the Supporting Information for detailed twisting angles between the D and A units). Herein, the high twisting confirmations, extended conjugation, stabilization of LUMO, and reduction in the HOMO–LUMO gap help to achieve red/NIR TADF emission in the investigated emitters.

**Table 1:** Photophysical Properties of D–A Compounds **B1**–**B4** in Cyclohexane^a^

compd	λ_abs_ (nm)	λ_em_ (nm)	S_1_ (eV)	T_1_ (eV)	Δ*E*_ST_^adia^ (eV)	*k*_f_ (10^6^ s^–1^)	λ_T_ (eV)	SOC^S_1_^ (cm^–1^)	*k*_ISC_ (10^7^ s^–1^)	λ_S_ (eV)	SOC^T_1_^ (cm^–1^)	*k*_RISC_ (s^–1^)
**B1**	474	618	2.01	1.72	0.334	6.99	0.232	0.41	5.81	0.241	0.59	1.03 × 10^2^
**B2**	460	589	2.10	1.95	0.244	1.06	0.162	0.45	8.69	0.193	0.73	7.36 × 10^3^
**B3**	603	927	1.34	1.22	0.105	0.14	0.062	0.18	2.53	0.453	0.54	4.86 × 10^4^
**B4**	511	670	1.85	1.92	0.044	0.45	0.007	0.17	1.34	0.162	0.34	1.97 × 10^6^

a λ_abs_ and λ_em_ are the wavelengths of the absorption and emission maxima, respectively. Excited energies were obtained at their respective equilibrium geometries.

**Table 2:** Photophysical Properties of D–A Compounds **C1**–**C4** in Cyclohexane^a^

compd	λ_abs_ (nm)	λ_em_ (nm)	S_1_ (eV)	T_1_ (eV)	Δ*E*_ST_^adia^ (eV)	*k*_f_ (10^6^ s^–1^)	λ_T_ (eV)	SOC^S_1_^ (cm^–1^)	*k*_ISC_ (10^7^ s^–1^)	λ_*S*_ (eV)	SOC^T_1_^ (cm^–1^)	*k*_RISC_ (s^–1^)
**C1**	478	706	1.76	1.62	0.302	1.042	0.193	0.32	3.28	0.286	0.65	5.60 × 10^2^
**C2**	473	671	1.85	1.82	0.206	1.185	0.111	0.35	4.32	0.213	0.68	2.96 × 10^4^
**C3**	587	1125	1.10	1.26	0.134	0.153	0.066	0.24	2.91	0.224	0.51	1.84 × 10^5^
**C4**	533	773	1.60	1.72	0.056	0.535	0.009^b^	0.15	0.12	0.151	0.26	9.71 × 10^5^

a λ_abs_ and λ_em_ are the wavelengths of the absorption and emission maxima, respectively. Excited energies were obtained at their respective equilibrium geometries.

b λ_T_ for **C4** was calculated as the reverse of the expression given in Molecular Design and Theoretical Methods.

**Table 3:** Photophysical Properties of D–A Compounds **D1**–**D4** in Cyclohexane^a^

compd	λ_abs_ (nm)	λ_em_ (nm)	S_1_ (eV)	T_1_ (eV)	Δ*E*_ST_^adia^ (eV)	*k*_f_ (10^6^ s^–1^)	λ_T_ (eV)	SOC^S_1_^ (cm^–1^)	*k*_ISC_ (10^7^ s^–1^)	λ_S_ (eV)	SOC^T_1_^ (cm^–1^)	*k*_RISC_ (s^–1^)
**D1**	464	583	2.13	1.33	0.373	0.059	0.247	0.99	27.16	0.389	1.38	1.40 × 10^2^
**D2**	495	625	1.98	1.78	0.121	0.034	0.115	0.04	0.121	0.263	0.30	6.61 × 10^4^
**D3**	587	987	1.26	1.17	0.019	0.014	0.002	0.28	13.69	0.583	2.28	1.44 × 10^6^
**D4**	551	713	1.74	1.69	0.014	0.013	0.017^b^	0.02	0.045	0.225	0.19	9.69 × 10^5^

a λ_abs_ and λ_em_ are the wavelengths of the absorption and emission maxima, respectively. Excited energies were obtained at their respective equilibrium geometries.

b λ_T_ for **D4** was calculated as the reverse of the expression given in Molecular Design and Theoretical Methods.

**Table 4:** Photophysical Properties of D–A Compounds **E1**–**E4** in Cyclohexane^a^

compd	λ_abs_ (nm)	λ_em_ (nm)	S_1_ (eV)	T_1_ (eV)	Δ*E*_ST_^adia^ (eV)	*k*_f_ (10^6^ s^–1^)	λ_T_ (eV)	SOC^S_1_^ (cm^–1^)	*k*_ISC_ (10^7^ s^–1^)	λ_S_ (eV)	SOC^T_1_^ (cm^–1^)	*k*_RISC_ (s^–1^)
**E1**	446	644	1.93	1.38	0.383	6.352	0.283	0.50	8.60	0.430	0.67	2.05 × 10^1^
**E2**	517	707	1.75	1.56	0.203	4.937	0.126	0.21	2.03	0.248	0.31	6.02 × 10^3^
**E3**	517	1217	1.02	1.26	0.071	0.085	0.049	0.23	5.57	0.454	0.63	1.41 × 10^5^
**E4**	598	831	1.49	1.44	0.055	2.222	0.010	0.08	0.24	0.194	0.12	1.29 × 10^5^

a λ_abs_ and λ_em_ are the wavelengths of the absorption and emission maxima, respectively. Excited energies were obtained at their respective equilibrium geometries.

We can confirm the large structural changes between the S_0_ and S_1_ states through the calculated D–A twisting angles (see Table S1). The contribution of possible vibronic transitions may result in a broad emission spectrum for the studied D–A compounds. However, the compounds presented a significant TDDFT oscillator strength (*f*) values for the 0–1 transitions in the range 10^–2^–10^–3^, enabling the the S_1_ → S_0_ CT emission. The photophysics of singlet and triplet excited states of the studied compounds is described below within the TDDFT framework.

### B Photophysics

The theoretically predicted absorption and fluorescence spectra of DBP in the nonpolar solvent cyclohexane (ε = 2.0165) are in close agreement with experiment^15^ (see Tables S2 and S3). The same solution state was used to investigate the photophysical properties of all the D–A compounds. Keeping in mind that increasing solvent polarity red-shifts the emission spectrum to longer wavelengths, we only considered the above low-polarity solvent to predict the possibility of the fluorescence emission even in a nonpolar solvent.

**Figure 6:**
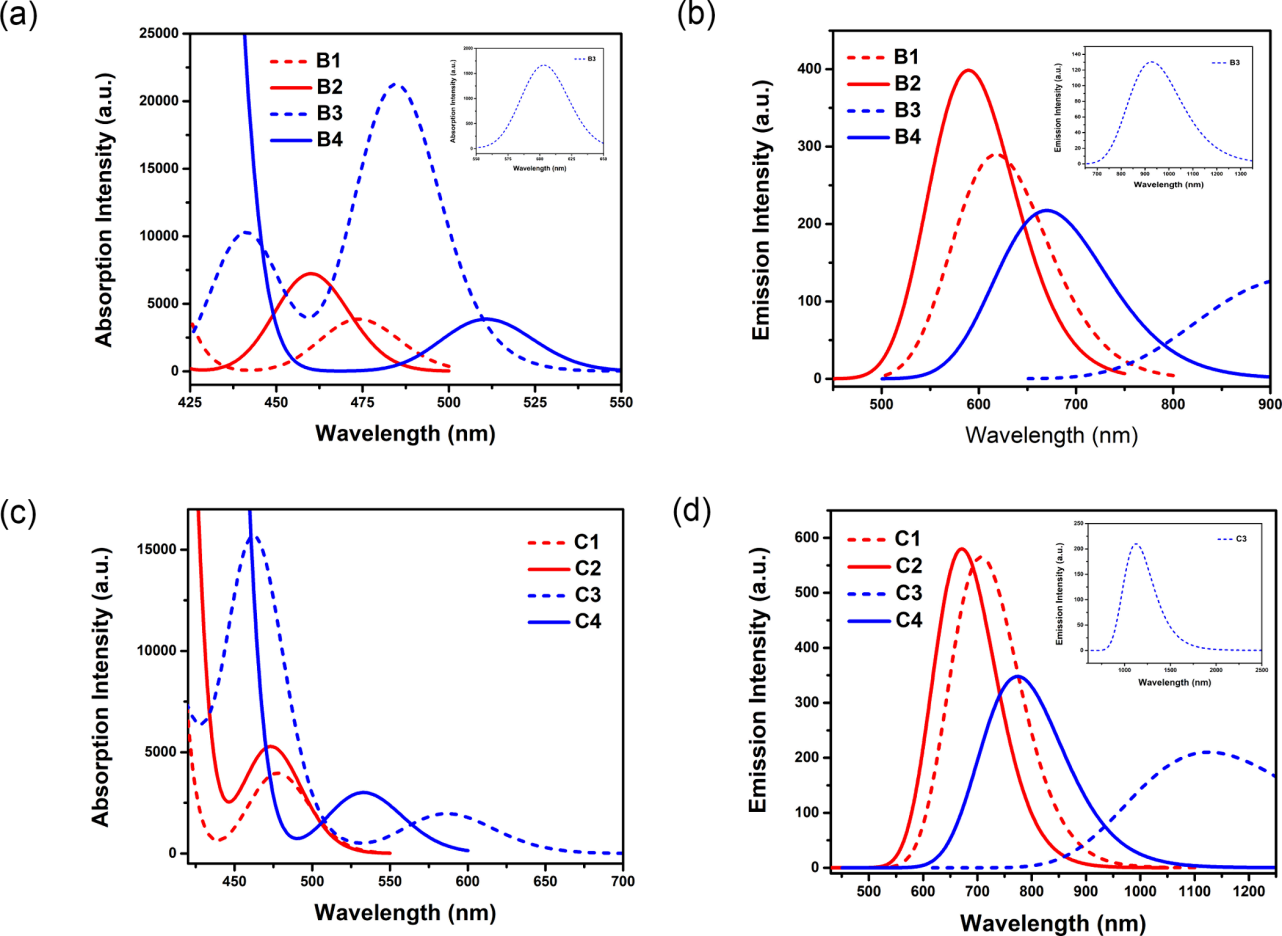
Computed absorption spectra (a) and (c) and emission spectra (b) and (d) showing intensity for peak wavelengths for *para*-linked compounds **B1**–**B4** and **C1**–**C4** in cyclohexane solution state. Here the full width at half-maximum (fwhm) was fixed at 1200 and 3000 cm^–1^ for the absorption and emission spectra, respectively.

**Figure 7:**
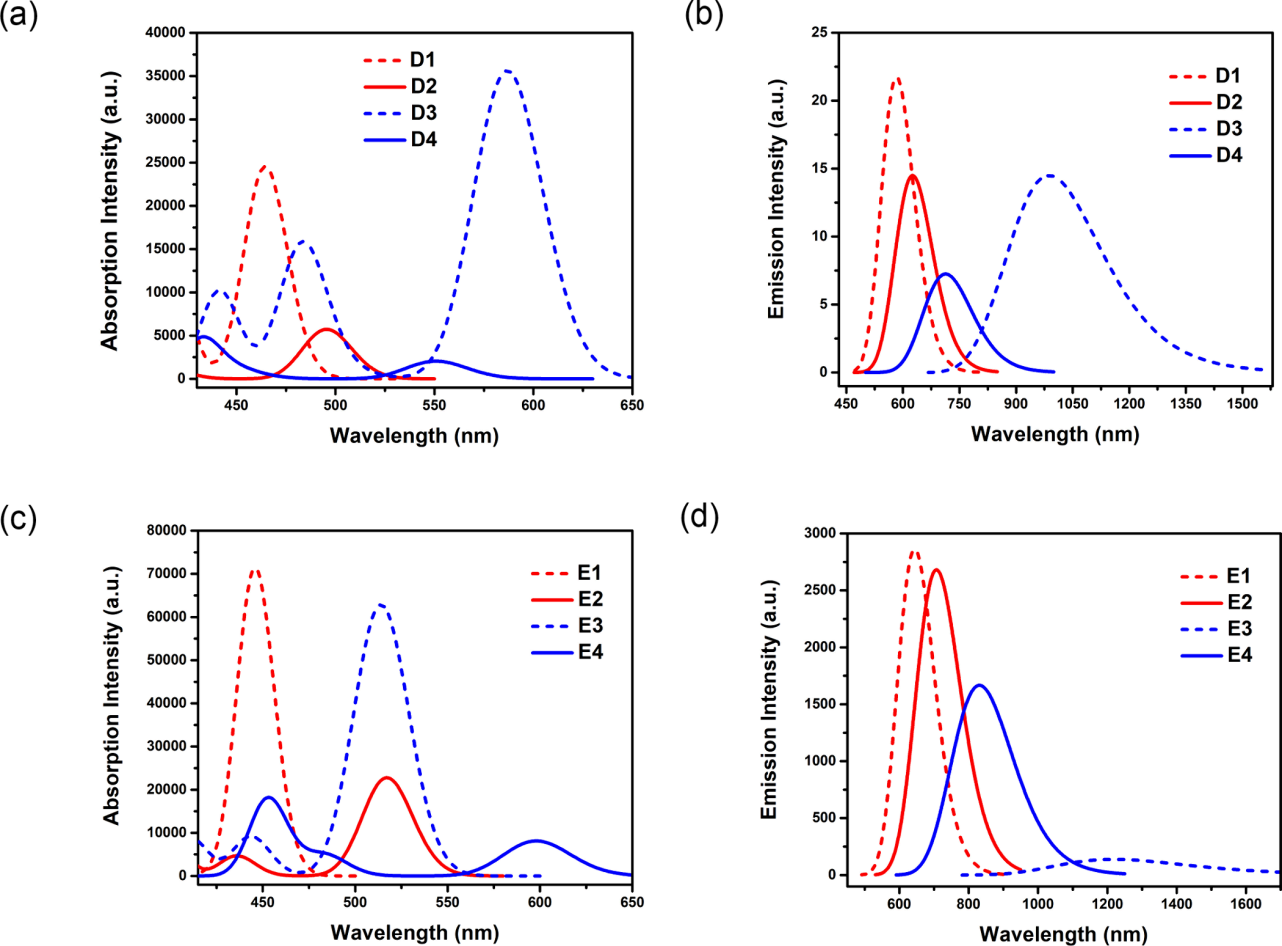
Computed absorption spectra (a) and (c) and emission spectra (b) and (d) showing intensity for peak wavelengths for *ortho*-linked compounds **D1**–**D4** and **E1**–**E4** in cyclohexane solution state. Here the full width at half-maximum (fwhm) was fixed at 1200 and 3000 cm^–1^ for the absorption and emission spectra, respectively.

The calculated absorption spectra and emission spectra of the studied compounds are presented in Figures 6 and 7, and the results are summarized in Tables S2–S11. For DBP, Yamaji et al. reported the single-crystal absorption to be about 350 nm, and the fluorescence wavelength in cyclohexane solution (λ_flu_^sol^) was 395 nm, whereas that in the solid state (λ_flu_^powder^) was 426 nm.^15^ Herein, the calculated lowest-energy absorption peak for DBP was obtained at 368 nm, and nitrogen substitutions red-shifted the absorption peak to 380–510 nm for **A1**–**A4** (see Figure S2 and Table S2). Large red shifts were found for **A1** and **A3** compared to the other two. Similarly, as summarized in Table S3 and shown in Figure S3, we obtained red shifts in fluorescence emission for **A1**–**A4** in contrast to the DBP molecule (λ_em_ = 433 nm). The large red shift may be because of the significant LUMO localization at the N-substituted fused benzene unit as well as the reduction in LUMO energy levels decrease the HOMO–LUMO gap in **A1** and **A3** compared to **A2** and **A4**, where for the latter we observed delocalization of LUMO on the whole molecule (see Figures 5 and S1). In the case of phosphorescence spectra, we noticed significant red shifts for compounds **A1**–**A4** compared to DBP. To give an example, the T_1_ state wavelength of **A1**/**A2** were calculated to 743/731 nm, where the parent DBP showed the lowest-energy phosphorescence at about 650 nm.^15^ Low triplet energy states in the studied N-substituted DBPs lead to only singlet-state emission. Here, the 4N-substituted compounds **A3** and **A4** showed triplet-state energy levels at 1.25 and 2.01 eV. The delocalization of the LUMO over the whole molecule could possibly result in both fluorescence and phosphorescence emission in the visible range for **A4**.

Likewise, in the case of both *para*- and *ortho*-linked D–A compounds, we noticed bathochromic shifts in absorption and emission spectra for **B1**/**B3**, **C1**/**C3**, **D1**/**D3**, and **E1**/**E3** compared with their corresponding compounds **B2**/**B4**, **C2**/**C4**, **D2**/**D4**, and **E2**/**E4**. The *para*-linked **B1**–**B4** showed a maximum absorption (λ_abs_) peak at 450–610 nm, which corresponds to a π–π* transition (see Figure 6a and Table S4). In addition, **B1** possesses a strong S_0_ → S_2_ absorption peak at 410 nm and an S_0_ → S_5_ peak at 384 nm, which are prominently HOMO–1 → LUMO and HOMO → LUMO+2 intermolecular CT (ICT) transitions between the acceptor and donor. Similarly, we observed a strong absorption S_0_ → S_2_ peak at 394 nm, an S_0_ → S_4_ peak at 484 nm, and an S_0_ → S_3_ peak at 424 nm for compounds **B2**, **B3**, and **B4**, respectively, where only for **B3** it is a HOMO–2 → LUMO intramolecular charge delocalization, and the other two show ICT nature. As summarized in Table S6, the calculated absorption bands for *para*-linked **C1**–**C4** are found in the range of 350–600 nm. For these compounds, the absorption peaks with high intensity are characterized by the mixing of CT and locally excited (LE) features, except for **C3**, where the strong S_0_ → S_3_ peak at 462 nm shows an intramolecular charge redistribution, which is defined by a HOMO–1 → LUMO transition. In the case of *ortho*-linked **D1**–**D4**, we found λ_abs_ nearly in the same range as their counterpart in the *para*-linked compounds, i.e., 450–600 nm (see Figure 7a). It should be noted that the strong absorption bands for **D1** and **D3** are characterized by S_0_ → S_1_ ICT transitions, whereas **D2** shows an LE-type transition and **D4** shows a strong S_0_ → S_4_ ICT absorption peak. Similarly, the strong absorption bands for *ortho*-linked compounds **E1**–**E3** are attributed to the S_0_ → S_1_ transition and **E4** is defined by S_0_ → S_3_ absorption with the mixing of CT and LE features. The maximum absorbance for these compounds falls in the range of 440–600 nm. Herein, the calculated λ_abs_ values for all the D–A compounds are characterized by HOMO → LUMO π–π* transitions.

From the TDDFT calculations for the singlet S_1_ state emission, we found that the *para*-linked compounds **B1**–**B4** emit in the red/NIR region at 600–950 nm, where **B3** showed NIR emission at 927 nm (*f* = 0.0018) (see Figure 6b and Table S5). Apparently, the broad emission spectrum for **B3** is due to the significant S_1_ state ICT character, which originates in the highly twisted nature of the molecular skeleton with twisting angle −70.2°/–72.7° and effective separation between the HOMO and LUMO (see Figure 1). Among the *para*-linked DMDPA donor-based compounds **C1**–**C4**, **C3** possesses a relatively strong ICT feature and small dipole moment at the S_1_ state, which results in IR emission at 1125 nm (*f* = 0.0029), whereas the others show emission in the range of 650–800 nm (see Figure 6d and Table S7). The calculated S_1_ emissions for *ortho*-linked compounds **D1**–**D4** are noticed in the range of 580–1000 nm, where **D3** and **D4** emit at 987 nm (*f* = 0.0002) and 713 nm (*f* = 0.0001), respectively. Herein (see Figure 7b and Table S9), the more than 10 times smaller calculated emission intensity is due to the strong S_1_ state ICT character derived from the significantly large D–A twisting angles for **D1**–**D4** compared with *para*-linked **B1**–**B4**. The *ortho*-linked compounds **E1**–**E4** show a coincidental broad emission spectrum range of 640–1220 nm (see Figure 7d and Table S11) as the *para*-linked compounds **C1**–**C4**. To summarize, the studied compounds emit in the red/NIR region with a highly twisted D–A molecular skeleton and effective separation between HOMO and LUMO, which suggest small singlet–triplet energy gaps (Δ*E*_ST_). The large degree of charge-transfer character and large Stokes shifts lead to broad emission spectra for the compounds with larger numbers of nitrogen substitutions.

Additionally, accounting for the higher Hartree–Fock (HF) exchange, we considered the hybrid functional which is a mixture of DFT and 50% exact HF exchange, i.e., the Becke-Half and Half-LYP (BHandHLYP) functional, to investigate the CT state and photophysics of studied compounds. The TDDFT/BHandHLYP/6-31+G(d) calculations were performed using the DFT/B3LYP/6-31+G(d)-optimized S_0_ geometries. The nature of the S_1_ states’ CT character calculated at the BHandHLYP level (see Figures S7 and S8) were found very similar as that of the B3LYP calculations (Figures 1–4). However, in the case of the TDDFT results, as a result of including 50% exact HF exchange into the hybrid density functional, we noticed underestimated absorption and emission wavelengths for some studied compounds, e.g., **B1**–**E1** and **B4**–**E4**. The detailed TDDFT/BHandHLYP/6-31+G(d) calculations are summarized in Table S20.

We then investigated the lowest triplet state, T_1_, for the studied D–A compounds in the cyclohexane solution state. It is predicted that among the *para*-linked compounds, **B1** and **C1** show T_1_ wavelengths above 700 nm with a large Δ*E*_ST_ > 0.3 eV. Phosphorescence emission for **B2** was found in the visible range at 634 nm alongside its fluorescence emission at 441 nm, suggesting dual emission at room temperature. Remarkably, this is because of the higher S_1_ → T_1_ intersystem crossing rate (*k*_ISC_ = 8.69 × 10^7^ s^–1^). Similarly, phosphorescence emission of **C2** calculated at 682 nm relates to *k*_ISC_ = 4.32 × 10^7^ s^–1^. The *ortho*-linked compounds **D1** and **E1** present a T_1_ wavelength above 900 nm. The higher-nitrogen-substituted compounds such as **B3**/**B4**, **C3**/**C4**, **D3**/**D4**, and **E3**/**E4** were found to possess small Δ*E*_ST_ which significantly increases the T_1_ → S_1_ reverse intersystem crossing rate (*k*_RISC_). The detailed characteristics of these compounds are analyzed in the next section.

### C Small Δ*E*_ST_ and TADF Emission

**Figure 8:**
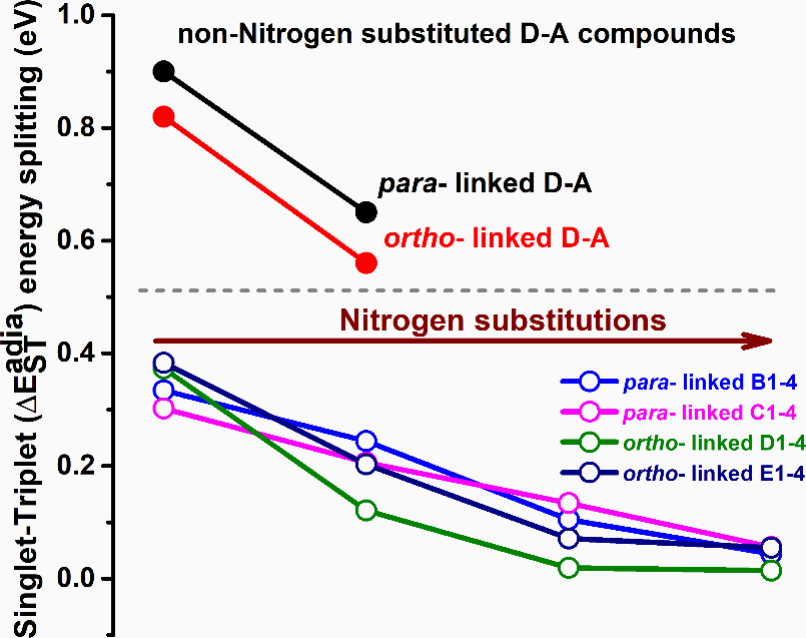
Variation of calculated adiabatic singlet–triplet energy splitting (Δ*E*_ST_^adia^) with and without nitrogen substitution of the D–A compounds.

In cyclohexane solvent medium, the TDDFT method with the B3LYP hybrid functional provides small Δ*E*_ST_ for the studied nitrogen-substituted D–A compounds. Evidently, the increased number of N substitutions significantly reduces the Δ*E*_ST_ by stabilizing the S_1_ state with respect to the T_1_ state. For instance, *para*-linked **B1** comprises S_1_ at 2.01 eV and T_1_ at 1.72 eV with an adiabatic singlet–triplet energy gap (Δ*E*_ST_^adia^) of 0.334 eV, whereas compound **B4** possesses S_1_ (^1^CT) and T_1_^3^(CT+LE) at 1.85 and 1.95 eV and a tiny Δ*E*_ST_^adia^ gap of 0.044 eV. The coupling between ^1^CT at 1.34 eV and ^3^(CT+LE) at 1.22 eV for **B3** results in a small Δ*E*_ST_^adia^ value of 0.105 eV (see Table 1). Similarly, among the *para*-linked compounds **C1**–**C4**, we calculated smaller Δ*E*_ST_^adia^ such as 0.134 and 0.056 eV for **C3** and **C4**, respectively. T_1_ states for all the *para*-linked D–A compounds show CT+LE character with large CT weight, whereas S_1_ states are of prominent CT nature. We observed substantial stabilization of both S_1_ and T_1_ energy levels for *ortho*-linked **D3** (1.26 and 1.17 eV) compared to **D1** (2.13 and 1.33 eV) and therefore a tiny Δ*E*_ST_^adia^ value of 0.019 eV. For **D3**, the T_1_ state presents mixing of CT and LE features which are composed of HOMO → LUMO (CT), HOMO–1 → LUMO (CT), HOMO–2 → LUMO (LE), HOMO–3 → LUMO (LE), and HOMO–4 → LUMO (LE) transitions. As summarized in Table 3, compounds **D2** and **D4** show only stabilization of the S_1_ state with respect to **D1**, whereas the opposite happens for the T_1_ states. Significant ICT between the acceptor and donor in both the S_1_ and T_1_ states of *ortho*-linked **D4** leads to a very tiny Δ*E*_ST_^adia^ of 0.014 eV. In case of *ortho*-linked **E1**–**E4**, the S_1_ and T_1_ energy levels are estimated to be 1.93 and 1.38 eV for **E1**, whereas **E3** and **E4** are composed of S_1_, T_1_ at 1.02, 1.26 eV and 1.49 eV, 1.44 eV, respectively. We calculated the tiny Δ*E*_ST_^adia^ to be 0.071 and 0.055 eV for **E3** and **E4**, respectively (see Table 4). In Figure 8, we present a graphical relationship between the calculated Δ*E*_ST_^adia^ with and without nitrogen substitutions for the studied D–A compounds. The above findings suggest predominant population of T_1_ from the S_1_ state with effective RISC, which is due to the calculated tiny Δ*E*_ST_^adia^ in some of the studied nitrogen-substituted D–A compounds. Therefore, we can conclude that TADF emission is present for those compounds.

**Figure 9:**
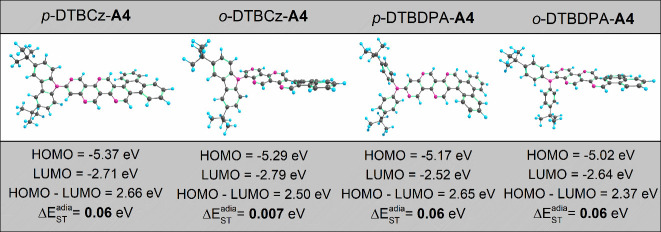
Optimized structures and calculated HOMO/LUMO energies, HOMO–LUMO gaps, and adiabatic singlet–triplet energy splittings Δ*E*_ST_^adia^ of the studied *tert*-butylated donor- and four-nitrogen-substituted acceptor-based D–A compounds.

To take advantage of strong electron donors, we considered *tert*-butylated DMCz and DMDPA, i.e., DTBCz and DTBDPA, respectively, and designed D–A compounds with 4N substitution of the **A4** acceptor molecule. The optimized structures of these compounds, namely, *p*-DTBCz–**A4**, *o*-DTBCz–**A4**, *p*-DTBDPA–**A4**, and *o*-DTBDPA–**A4**, are given in Figure 9. These D–A combinations provide a narrower Δ*E*_ST_^adia^ in the range of 0.007–0.057 eV, where the compound *o*-DTBCz–**A4** possesses the smallest Δ*E*_ST_^adia^. As we can notice from Figures 5 and 9, *p*-DTBCz–**A4**, *p*-DTBDPA–**A4**, *o*-DTBCz–**A4**, and *o*-DTBDPA–**A4** show similar HOMO/LUMO gaps as **B4**, **C4**, **D4**, and **E4**, respectively. However, the presence of the *tert*-butyl side group reduces the singlet–triplet energy splittings, particularly for *o*-DTBCz–**A4**. This suggests a more efficient TADF mechanism in this type of compounds.

For comparison, we estimated the vertical singlet–triplet energy gaps at S_0_ (Δ*E*_S_1_T_1__^vert^]^S_0_^), S_1_ (Δ*E*_S_1_T_1__^vert^]^S_1_^), and T_1_ (Δ*E*_S_1_T_1__^vert^]^T_1_^) state geometries using the same level of theory in cyclohexane solution. Simulated Δ*E*_S_1_T_1__^vert^ values are summarized in Tables S12–S17. The *para*-linked compounds like **B4** and **C4** presented small Δ*E*_S_1_T_1__^vert^]^S_0_^ values such as 0.166 and 0.220 eV, where the counterpart *ortho*-linked **D4** and **E4** showed values of 0.041 and 0.126 eV, respectively. Apparently, we calculated tiny Δ*E*_S_1_T_1__^vert^]^S_1_^ for both *para*-linked and *ortho*-linked compounds. These small Δ*E*_S_1_T_1__^vert^]^S_1_^ values suggest effective couplings and ISC between singlet and triplet states. Additionally, considering the lowest excited energy state, i.e., the T_1_ state, the calculated Δ*E*_S_1_T_1__^vert^]^T_1_^ is about 0.2 eV (see Tables S16 and S17), thus realizing the RISC process for compounds **B4**, **C4**, **D4**, and **E4**.

Moreover, the role of high-lying excited states is crucial in determining the RISC efficiency. In general, a thermal equilibrium between the S_1_ and T_1_ states leads to fast RISC for TADF emitters.^55^ This is further associated with the large triplet–triplet energy gap (Δ*E*_T_1_T_2__) that reduces the interference of high-lying excited states. However, a small Δ*E*_T_1_T_2__ leads to undesired nonradiative deactivation processes that slow down RISC between adjacent singlet and triplet energy levels.^56,57^ This fact has also been investigated in this study. To calculate Δ*E*_T_1_T_2__, we considered the optimized geometries of the S_0_, S_1_, and T_1_ states, and the values are given in Tables S12–S17. We investigated the behavior of Δ*E*_T_1_T_2__ in these three states. At the T_1_ geometry, the predicted large Δ*E*_T_1_T_2__^vert^]^T_1_^ energy gap suggests a reduction of the undesired nonradiative deactivation processes and thus facilitates the RISC process. In general, the calculated vertical Δ*E*_T_1_T_2__ energy gaps at the S_0_ (Δ*E*_T_1_T_2__^vert^]^S_0_^), S_1_ (Δ*E*_T_1_T_2__^vert^]^S_1_^), and T_1_ (Δ*E*_T_1_T_2__^vert^]^T_1_^) geometries show a linear dependence with increasing number of N substitutions (see Tables S12–S17 and Figure S5). In particular, the large Δ*E*_T_1_T_2__ for *para*-linked **B4** and **C4** and *ortho*-linked **D4** and **E4** suggests faster RISC from T_1_ to S_1_ and enables them to behave as efficient TADF emitters.

At the beginning, we considered the donor–acceptor framework without N substitutions in the acceptor DBP molecule and investigated their photophysical properties. The optimized structures of these D–A compounds are given in Figure S4. Although there is a significant twisting in these D–A skeletons, their photophysical properties (in cyclohexane solution state) suggest a lower T_1_ state relative to the S_1_ state, and hence, we computed a large Δ*E*_ST_^adia^ (see Figure 8). Figure S4 shows schematic pictures for the calculated excited energies and Δ*E*_ST_^adia^ for these non-nitrogen-substituted D–A compounds. The estimated Δ*E*_ST_^adia^ are 0.90, 0.82, 0.65, and 0.56 eV for *p*-DMCz–DBP, *o*-DMCz–DBP, *p*-DMDPA–DBP, and*o*-DMDPA–DBP, respectively. Herein, relatively low T_1_ and large Δ*E*_ST_^adia^ energies are accompanied by very small SOC values as well as small RISC from T_1_ to S_1_. Hence, these non-nitrogen-substituted D–A compounds do not show TADF emission, and only fluorescence emission is observed. The direct S_1_ → S_0_ fluorescence emission wavelengths and calculated oscillator strengths are presented in Figure S4. This suggests that introduction of N atoms into the acceptor DBP substantially reduces Δ*E*_ST_^adia^ for the designed N-substituted D–A compounds, making them effective for the RISC process and hence TADF emission.

To investigate the TADF emission properties of the studied designed D–A compounds, we performed singlet–triplet SOC calculations followed by calculations of direct and reverse intersystem crossing rates (*k*_ISC_ and *k*_RISC_). As we discussed earlier, the singlet–triplet energy splittings (Δ*E*_ST_^adia^) are predicted to be very small for the designed D–A compounds such as **B3**/**B4**, **C3**/**C4**, **D3**/**D4**, and **E3**/**E4** (see Tables 1–4), suggesting the capability to realize efficient T_1_ → S_1_ RISC channels and high triplet exciton utilization in OLEDs. Moreover, we also predicted larger SOC matrix element values at the T_1_ geometry than at the S_1_ geometry (see Tables 1–4), which implies effective T_1_ → S_1_ interaction for the studied compounds. In addition, the calculated ^1^CT state and mixed ^3^(CT+LE) state for the studied D–A compounds suggest efficient SOC matrix elements according to the El Sayed rule for the intersystem crossing process.^8,58^

However, as summarized in Tables 1–4 for compounds **B1**/**B2**, **C1**/**C2**, **D1**, and **E1**/**E2** we noticed very low *k*_RISC_ compared to their calculated radiative fluorescence decay rates (*k*_f_). For instance, *para*-linked **B1**/**B2** and *ortho*-linked **E1**/**E2** show *k*_RISC_ on the order of 10^2^–10^5^ lower than *k*_f_. The direct *k*_ISC_ rates for these compounds were calculated to be on the order of 10^7^–10^8^ s^–1^. Therefore, in this case, upconversion of T_1_ excitons into S_1_ is highly improbable, and we can only predict direct S_1_ → S_0_ fluorescence emission. As already noted, fast singlet S_1_ → S_0_ decay is identified as the prompt fluorescence (PF), whereas the slower decay can be referred to as the delayed fluorescence (DF), which comes from the reverse intersystem crossing. Hence, no delayed fluorescence emission is predicted for the studied compounds with a low number of nitrogen substitutions (2N). Similarly, computed relevant T_1_ energies and *k*_ISC_ values on the order of 10^7^ s^–1^ suggest the possibility of phosphorescence emission (in the range of 600–800 nm) in these compounds at room temperature.

**Figure 10:**
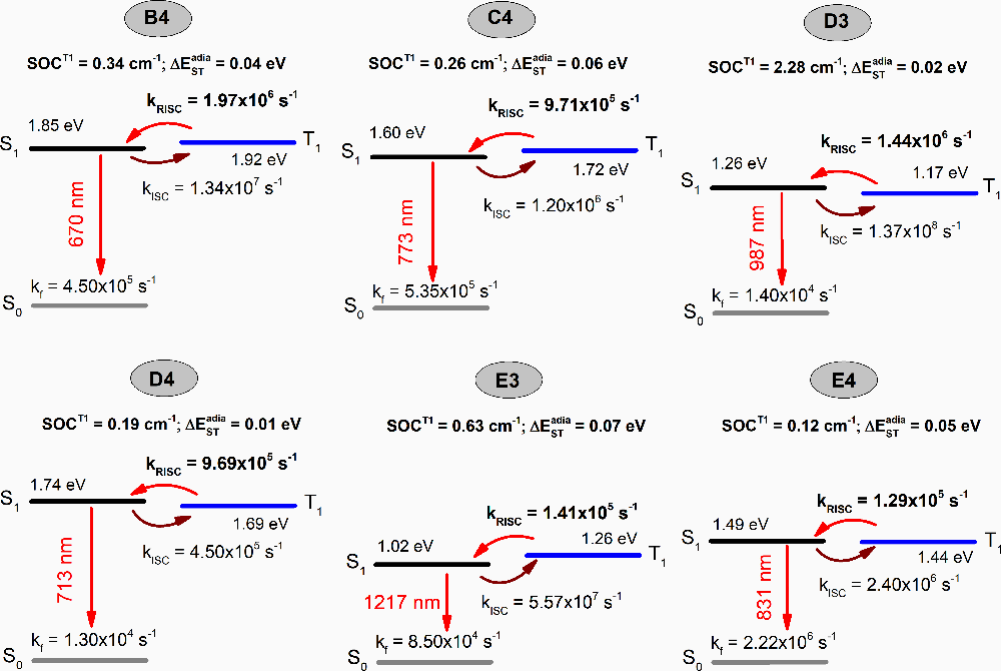
Schematic representation of excited state characteristics, including singlet/triplet energy levels, adiabatic singlet–triplet energy splittings (Δ*E*_ST_^adia^), spin–orbit coupling (SOC), ISC/RISC rate constants, radiative decay rates (*k*_f_), and delayed fluorescence emission wavelengths for the higher-nitrogen-substituted (4N) D–A compounds.

In comparison, at room temperature, *k*_RISC_ for the compounds with a higher number of nitrogen substitutions (4N) is 10–100 times higher than *k*_f_ (see Tables 1–4 and Figure 10). A summary of the predicted important photophysical properties is presented in Figure 10. One should note here that when we increase the number of nitrogen substitutions, we encounter a decrease in *k*_ISC_ and increase in *k*_RISC_. This indicates that nitrogen substitutions pave the way for effective triplet exciton spin flip as well as delayed fluorescence emission. The calculated SOC^T_1_^ matrix element of 0.34 cm^–1^ resulted in *k*_RISC_ = 1.97 × 10^6^ s^–1^ for *para*-linked **B4**. This causes delayed red fluorescence emission at 670 nm. Similarly, we calculated *k*_RISC_ ≈ 10^5^–10^6^ s^–1^ leading to NIR TADF emission at 1125 nm for **C3** and 773 nm for **C4**. The designed *ortho*-linked D–A compounds presented NIR delayed fluorescence emission. For example, **D3** showed delayed emission at 987 nm with a large SOC^T_1_^ matrix element of 2.28 cm^–1^ and *k*_RISC_ = 1.44 × 10^6^ s^–1^. With about 75 times higher *k*_RISC_ than *k*_f_, we predict delayed fluorescence at 713 nm for compound **D4**. In the case of compounds **E3** and **E4**, the *k*_RISC_ values of 1.41 × 10^5^ and 1.29 × 10^5^ s^–1^ recycle the T_1_ excitons into the S_1_ state and lead to emission at 1217 and 831 nm, respectively.

## IV Conclusions

As described in this work, several factors are important in the design of red and near-IR TADF emitters, foremost the increase in molecular rigidity, significant twisting separation between donors and acceptors, strong electron-donating and electron-accepting strengths, and LUMO stabilization. In this paper, we have demonstrated the effect of nitrogen functionalization for the development of red and near-IR thermally activated delayed emission in D–A-type organic emitters and illustrated the effect by studying symmetric nitrogen substitution in PAHs with seven fused benzene rings. We designed new acceptor molecules with symmetric nitrogen substitutions in the dibenzo[*a*,*c*]piecene (DBP) core. With the newly designed acceptor and commonly used strong donors such as dimethylcarbazole (DMCz) and dimethyldiphenylamine (DMDPA), we proposed and studied 16 D–A-type OLED emitters. Within the DFT framework, we performed a systematic investigation of molecular structures, conformations, structure–property relationships, and photophysical properties of such D–A-type PAH-based OLED emitters. We present our results as follows:

(a) Nitrogen substitutions lead to LUMO stabilization and reduced HOMO–LUMO energy gaps. Among the designed compounds, we predict that the higher-nitrogen-substituted (4N) compounds show significant TADF behavior with small Δ*E*_ST_^adia^ values (<0.2 eV). However, the corresponding non-nitrogen-substituted D–A compounds presented a significantly larger Δ*E*_ST_^adia^ and hence are predicted to be unsuitable for TADF emission.
(b) The effect of *ortho* and *para* linkages of donor molecules have been studied. The *ortho*-linked D–A compounds presented relatively large twisting separation between the D and A moieties for the singlet excited S_1_ state and showed comparatively small singlet–triplet energy splittings as well.
(c) A detailed investigation of the singlet–triplet energy splitting for the studied compounds has been presented through calculations of both adiabatic and vertical energy gaps (Δ*E*_ST_^adia^ and Δ*E*_ST_^vert^). We calculated a smaller Δ*E*_ST_^adia^ in the range 0.01–0.13 eV for higher-nitrogen-substituted (4N) D–A compounds. Similarly, at the S_1_ geometry, the computed Δ*E*_ST_^vert^ values are found to be on the order of 0.007–0.13 eV. Additionally, we investigated the effect of strong donor strengths by considering *tert*-butyl-substituted donors. With these donors, we could predict significantly smaller Δ*E*_ST_^adia^ values, in particular, 0.007 eV for the *o*-DTBCz–**A4** compound.
(d) We computed large spin–orbit coupling (SOC) matrix element values on the order of 0.12–2.28 cm^–1^ at the T_1_ state, which are found to be larger than those at the S_1_ state geometry. This suggests significant T_1_ → S_1_ couplings and an effective RISC process.
(e) The higher T_1_ → S_1_ RISC rates are calculated to be on the order of 10^6^ s^–1^ and are also found to be about 10–100 times higher than the radiative fluorescence decay rates (*k*_f_). The calculated red emission peak at 670 nm of *para*-linked compound **B4** is due to the large *k*_RISC_ value of 1.97 × 10^6^ s^–1^. The ∼75 times higher RISC rate relative to *k*_f_ results in delayed fluorescence emission at 713 nm for the *ortho*-linked compound **D4**. Calculated *k*_RISC_ values of 1.44 × 10^6^ and 1.41 × 10^5^ s^–1^ result in near-IR delayed fluorescence emission at 987 and 1217 nm for the *ortho*-linked compounds **D3** and **E3**, respectively.

We can conclude that the employed theoretical methodology forms a reliable basis for designing D–A-type PAHs based on red/NIR TADF OLED emitters. The results provide thumb rules for designing effective emitters, like symmetric higher nitrogen functionalization giving LUMO stabilization and reduced HOMO–LUMO energy gaps and *ortho* linking of D–A compounds giving small Δ*E*_ST_^adia^, large SOC, and fast RISC, which we believe will encourage future development of novel PAH-based red/NIR OLED emitters.

## Supporting Information Available

Optimized structures and HOMO/LUMO charge density plots for DBP and N-substituted DBP compounds, dihedral angles in the S_0_ and S_1_ states of all D–A compounds, absorption and emission spectra of DBP and **A1**–**A4** compounds, large Δ*E*_ST_^adia^ and no TADF emission in non-nitrogen-substituted D–A compounds, results for absorption and emission properties of all studied compounds from TDDFT calculations and comparison with experiment, computed excitation energies and vertical singlet–triplet/triplet–triplet energy gaps at the S_0_, S_1_, and T_1_ state conformations, linear-fit presentation of calculated vertical triplet–triplet energy splitting gaps at the S_1_ and T_1_ state geometries, validation with experimental results for nitrogen-substituted PAH based emitters, calculated dihedral angles and HOMO/LUMO gaps using GD3 empirical dispersion, nature of charge transfer states and TDDFT results at the BHandHLYP level, and optimized S_0_ state coordinates for all compounds (PDF)
