# Correction to “Developing Red and Near-Infrared Delayed Fluorescence Emission in Nitrogen-Substituted Donor–Acceptor Polycyclic Hydrocarbon OLED Emitters: A Theoretical Study”

**DOI:** 10.1021/acs.jpca.5c04457

**Published:** 2025-07-14

**Authors:** Smruti Ranjan Sahoo, Glib V. Baryshnikov, Hans Ågren

In our original manuscript, there is an error on page no. 2400 in Figure 3(b) related to the optimized ground-state (S_0_) structure of compound **D3**. The figure shows the same structure as for compound **C3**. This is here replaced by the correct structure for **D3**. Below, we have provided the correct figure (Figure 3 in the manuscript).

**Figure 3:**
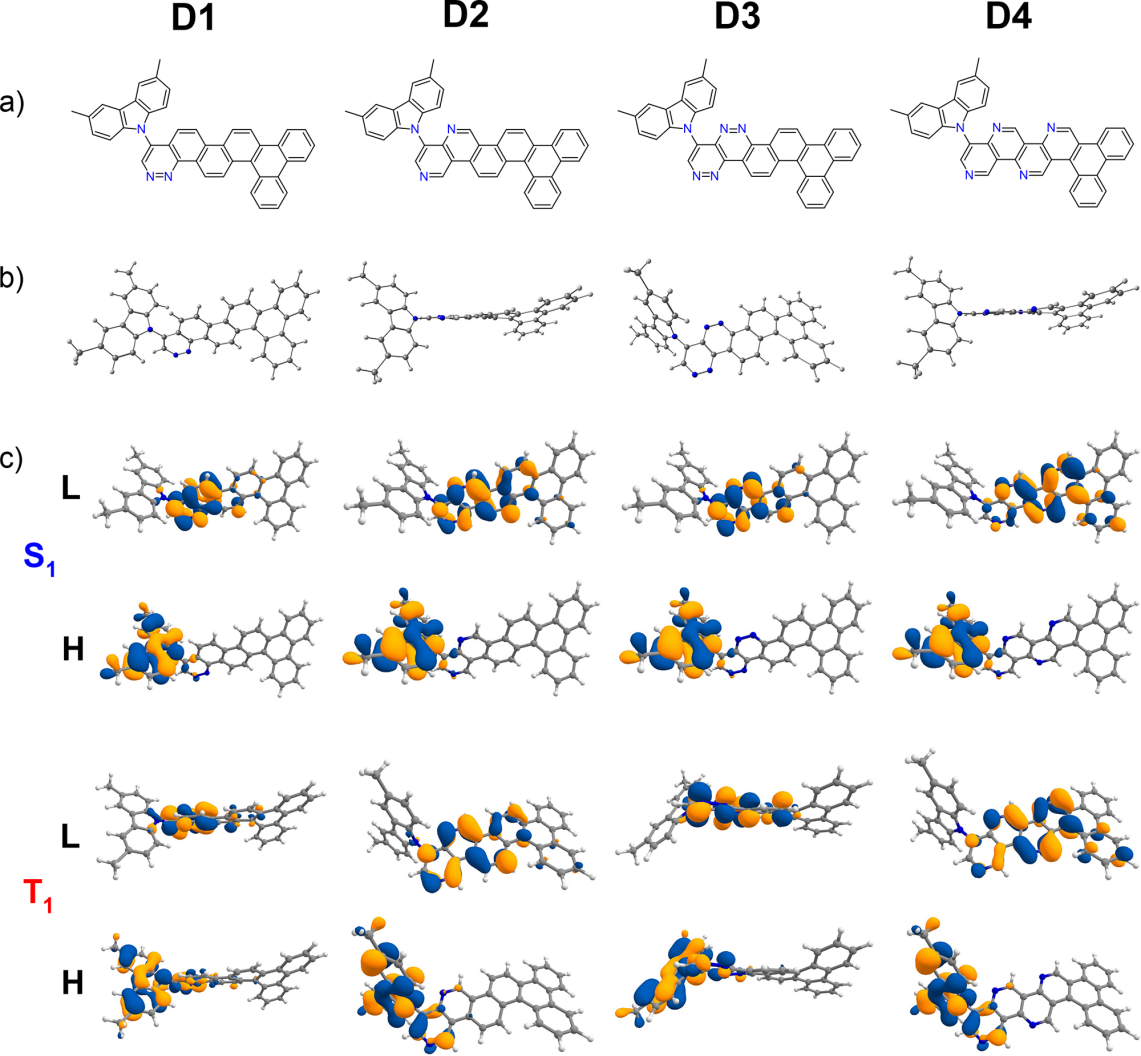
Chemical structures from theoretical calculations on compounds **D1**–**D4**. (a) Chemical structures of **D1**–**D4**. (b) B3LYP/6-31+G­(d)-optimized ground-state (S_0_) structures. (c) Calculated HOMO (H) and LUMO (L) charge density distribution plots for the excited states S_1_ and T_1_ obtained using the TDDFT/B3LYP/6-31+G­(d) method in cyclohexane solution state.

