# Supplementary material for: Developing Red and Near-Infrared Delayed Fluorescence Emission in Nitrogen-Substituted Donor–Acceptor Polycyclic Hydrocarbon OLED Emitters: A Theoretical Study
Source: J Phys Chem A. 2025 Feb 26;129(10):2396–410. doi: 10.1021/acs.jpca.4c07345 (PMC11912487; doi:10.1021/acs.jpca.4c07345)
Supplement: Supplementary file 1 — jp4c07345_si_001.pdf [file jp4c07345_si_001.pdf]

Supplementary Information

For

Developing Red and Near Infrared Delayed Fluorescence Emission in Nitrogen Substituted Donor-Acceptor Polycyclic Hydrocarbon OLED Emitters – A Theoretical Study

Smruti Ranjan Sahoo[1 2], *Glib V. Baryshnikov*[2*], *Hans Ågren*[1*]

[1]Division of X-ray Photon Science, Department of Physics and Astronomy, Box 516, SE-75120, Uppsala University, Sweden

[2]Laboratory of Organic Electronics, Department of Science and Technology, Linköping University, Norrköping 60174, Sweden

Description:

Optimized structures and HOMO/LUMO charge density plots for **DBP** and N-substituted **DBP**s, Dihedral angles at S_0 and S_1 state of all D-A compounds, Absorption and emission spectrum of **DBP** and **A1-4** compounds, Large ΔE_{ST}^{adia} gap and no TADF emission in non-nitrogen substituted D-A compounds, Results for absorption and emission properties of all studied compounds from TDDFT calculations, and comparison with experiment, Computed excitation energies, and vertical singlet-triplet/triplet-triplet energy gaps at S_0, S_1, and T_1 state confirmation, Linear-fit presentation of calculated vertical triplet-triplet energy splitting gaps at S_1 and T_1 state geometry, Validation with experimental results for nitrogen substituted PAH based emitters, Calculated dihedral angles and HOMO/LUMO using GD3 empirical dispersion, Nature of charge transfer states and TDDFT results at BHandHLYP level, Optimized S_0 state coordinates for all compounds.

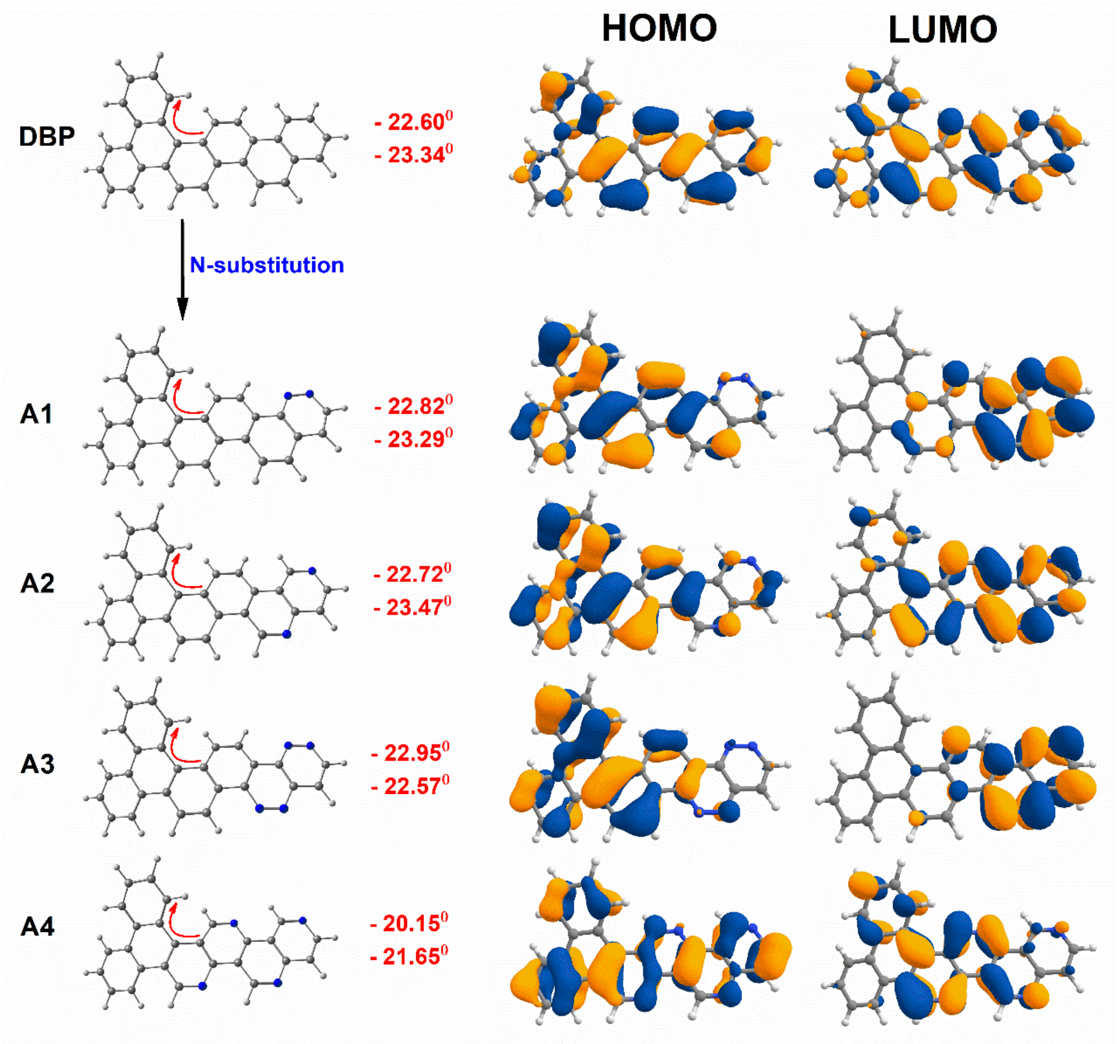

Fig. S1: Optimized structures of **DBP** and N-substituted DBPs with calculated twisting angles in between the benzene and picene units. The highest occupied molecular orbital (HOMO) and lowest unoccupied molecular orbital (LUMO) charge densities are shown on the right side.

Table S1: Calculated twisting angles (in degree) between donor and acceptor at ground state and singlet excited geometries of the studied compounds.

Compounds	At ground state (S_0)	At singlet excited state (S_1)
**B1**	-38.648/-42.862	-68.225/-65.247
**B2**	-38.448/-41.320	-60.740/-59.126
**B3**	-40.185/-36.091	-70.220/-72.739
**B4**	-40.405/-35.578	-55.754/-57.586
**C1**	-52.403/-47.754	-31.674/-26.248
**C2**	-53.785/-48.274	-30.937/-30.193
**C3**	-54.655/-50.080	-27.311/-30.429
**C4**	-54.417/-48.559	-54.417/-48.559
**D1**	66.301/58.195	90.474/90.441
**D2**	111.759/112.330	-90.165/-86.693
**D3**	-56.418/-46.756	88.288/91.584

**D4**	-67.920/-68.952	90.414/93.684
**E1**	-33.211/-53.340	-35.194/-18.846
**E2**	-35.713/-47.025	-23.686/-34.561
**E3**	-46.103/-54.926	-60.900/-6.487
**E4**	-46.064/-35.946	-34.701/-27.287

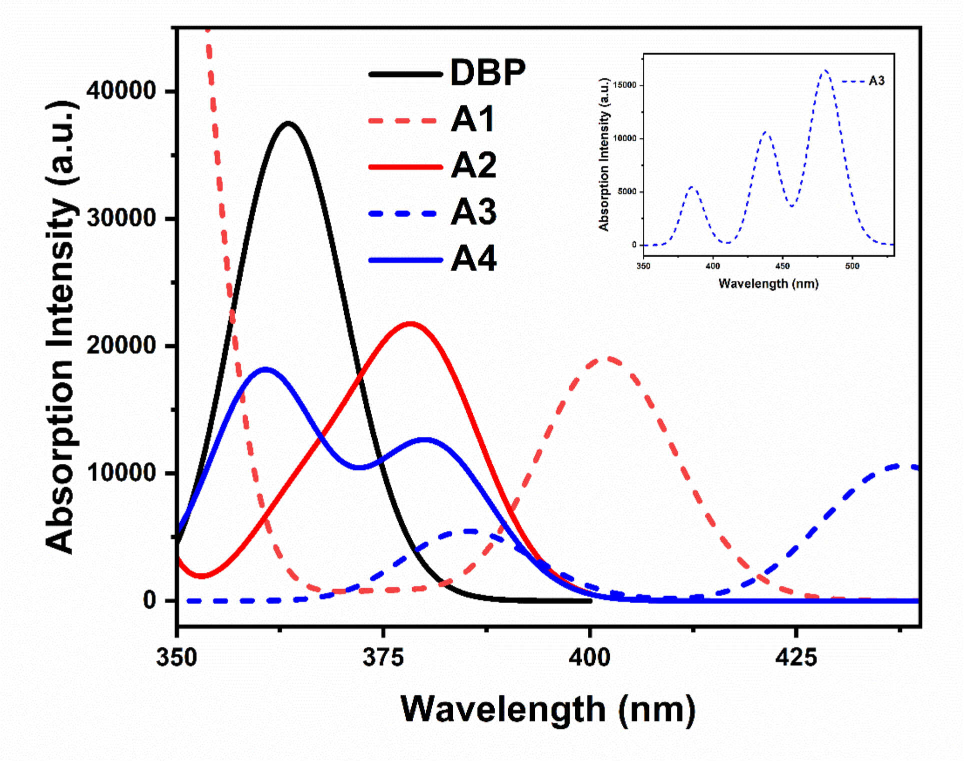

Fig. S2: Absorption spectrum showing intensity for peak wavelengths of absorption for **DBP** and **A1-4** compounds in cyclohexane solution state. The spectrum for **A3** (inserted one) shows whole range of absorption.

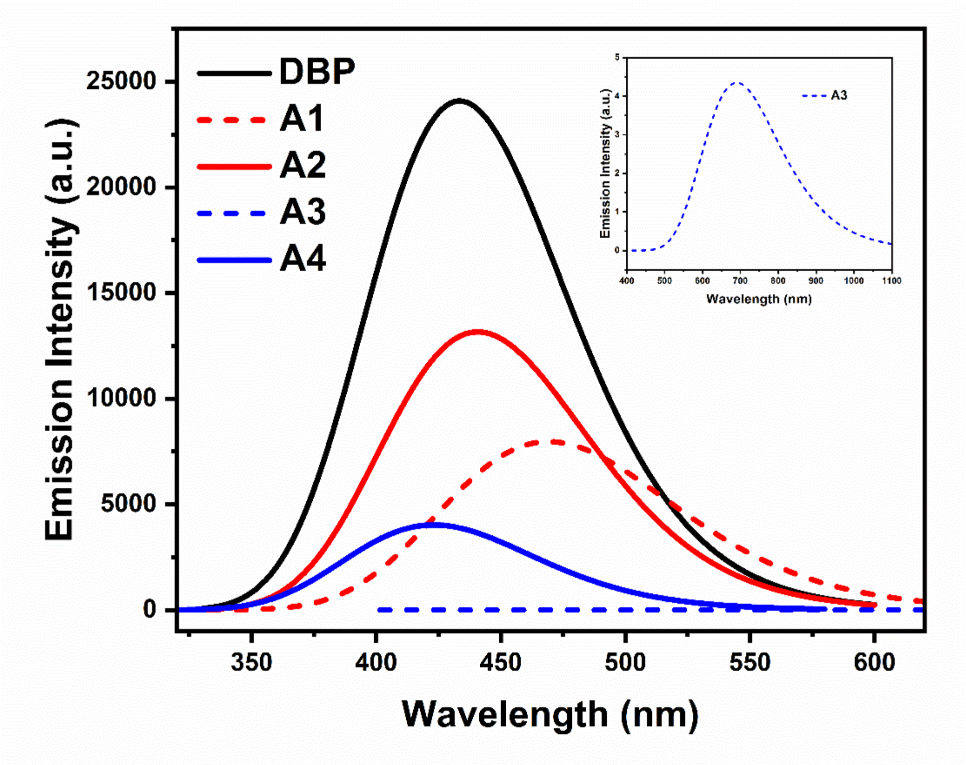

Fig. S3: Calculated emission spectrum showing intensity for peak wavelengths of emission for **DBP** and **A1-4** compounds in cyclohexane solution state. The maximum emission spectrum for **A3** is shown as inserted plot.

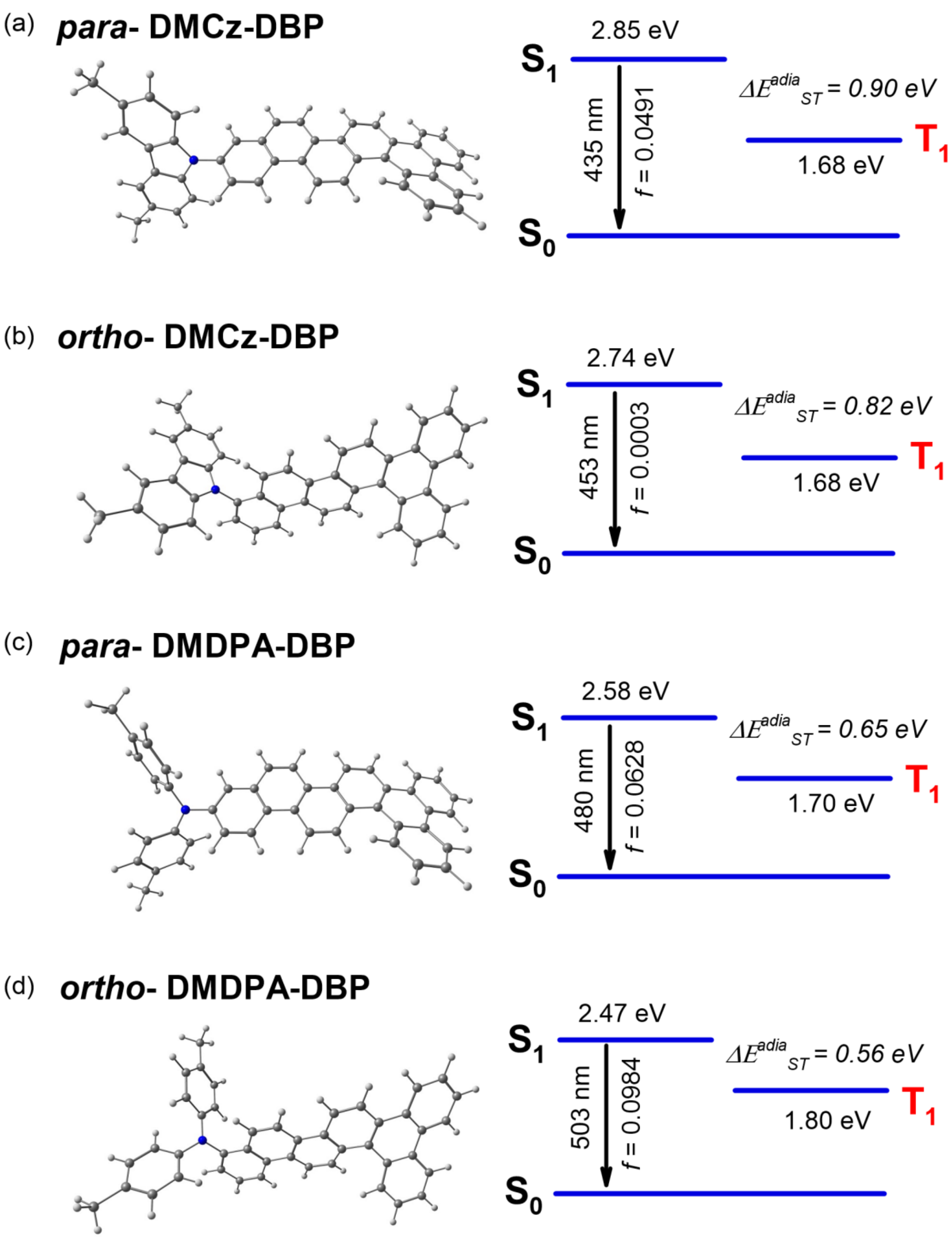

Fig. S4: (left side) Optimized geometry structures for ground state (S_0) of *para*- and *ortho*-linked donor-acceptor compounds. (right side) Schematic representation of the calculated excited energy levels, adiabatic singlet-triplet energy gap, $S_1 \rightarrow S_0$ fluorescence wavelength and oscillator strength values. Calculated large ΔE_{ST}^{adia} gap indicates no TADF emission and fluorescence emission being present for these D-A compounds.

Table S2: Calculated absorption for **DBP** and **A1-4** compounds in cyclohexane at B3LYP/6-31+G(d) level of theory. Experimental absorption data by Yamaji *et al.* is given here.

Compound	Excitations	Energy (eV)	Wavelength (nm)	Oscillator strength (f)	Experiment (nm)
**DBP**	S_1	3.37	368	0.0229	350
	S_2	3.42	363	0.1885	
	S_3	3.79	327	0.0518	
	S_4	3.82	324	1.6308	
	S_5	3.91	317	0.0255	
**A1**	S_1	3.08	402	0.1012	
	S_2	3.13	396	0.0048	
	S_3	3.31	375	0.0043	
	S_4	3.59	346	0.5270	
	S_5	3.66	339	0.1176	
**A2**	S_1	3.27	379	0.1119	
	S_2	3.39	366	0.0448	
	S_3	3.72	334	0.6615	
	S_4	3.79	328	0.0407	
	S_5	3.85	322	0.1389	
**A3**	S_1	2.44	509	0.0005	
	S_2	2.58	480	0.0905	
	S_3	2.83	438	0.0585	
	S_4	3.17	391	0.0050	
	S_5	3.23	384	0.0267	
**A4**	S_1	3.26	380	0.0681	
	S_2	3.44	360	0.0990	
	S_3	3.59	345	0.0022	
	S_4	3.75	331	0.4194	
	S_5	3.88	319	0.0062	

Table S3: Calculated S1 → S0 emission results for **DBP** and **A1-4** compounds in cyclohexane at B3LYP/6-31+G(d) level of theory. Experimental results by Yamaji *et al.* are given [a]in cyclohexane and [b]in powder state.

Compound	Energy (eV)	Wavelength (nm)	Oscillator strength (f)	Experiment (nm)
**DBP**	2.86	433	0.5540	395[a], 426[b]
**A1**	2.64	469	0.1832	
**A2**	2.81	441	0.3027	
**A3**	1.80	690	0.0001	
**A4**	2.93	423	0.0926	

Table S4: Calculated absorption results for **B1-4** DA in cyclohexane at B3LYP/6-31+G(d) level of theory.

Compound	Excitations	Energy (eV)	Wavelength (nm)	Oscillator strength (f)
**B1**	S_1	2.62	474	0.0213
	S_2	3.02	410	0.0907
	S_3	3.08	402	0.0044
	S_4	3.20	387	0.0773
	S_5	3.23	384	0.6558
**B2**	S_1	2.70	460	0.0399
	S_2	3.15	394	0.7094
	S_3	3.16	392	0.0114
	S_4	3.26	380	0.2121
	S_5	3.35	370	0.0426
**B3**	S_1	2.06	603	0.0092
	S_2	2.44	508	0.0020
	S_3	2.47	502	0.0058
	S_4	2.56	484	0.1150
	S_5	2.81	441	0.0568
**B4**	S_1	2.43	511	0.0213
	S_2	2.88	431	0.0001
	S_3	2.92	424	0.6185
	S_4	3.05	406	0.0063
	S_5	3.30	375	0.0607

Table S5: Calculated S1 → S0 emission results of **B1-4** DA compounds in cyclohexane at B3LYP/6-31+G(d) level of theory.

Compound	Energy (eV)	Wavelength (nm)	Oscillator strength (f)
**B1**	2.01	618	0.0040
**B2**	2.10	589	0.0055
**B3**	1.34	927	0.0018
**B4**	1.85	670	0.0030

Table S6: Calculated absorption results for **C1-4** DA in cyclohexane at B3LYP/6-31+G(d) level of theory.

Compound	Excitations	Energy (eV)	Wavelength (nm)	Oscillator strength (f)
**C1**	S_1	2.60	478	0.0365
	S_2	3.10	401	0.0380
	S_3	3.16	392	0.0777
	S_4	3.23	384	1.0529
	S_5	3.31	375	0.0090
**C2**	S_1	2.62	473	0.0486

	S_2	3.10	400	0.8491
	S_3	3.26	383	0.0253
	S_4	3.34	370	0.3778
	S_5	3.50	355	0.1517
**C3**	S_1	2.11	587	0.0181
	S_2	2.46	505	0.0041
	S_3	2.68	462	0.1424
	S_4	2.95	421	0.0343
	S_5	3.12	398	0.0912
**C4**	S_1	2.33	533	0.0277
	S_2	2.89	430	0.7549
	S_3	2.98	417	0.0616
	S_4	3.34	371	0.0604
	S_5	3.43	362	0.0828

Table S7: Calculated S1 → S0 emission results of **C1-4** DA compounds in cyclohexane at B3LYP/6-31+G(d) level of theory.

Compound	Energy (eV)	Wavelength (nm)	Oscillator strength (*f*)
**C1**	1.76	706	0.0078
**C2**	1.85	671	0.0080
**C3**	1.10	1125	0.0029
**C4**	1.60	773	0.0048

Table S8: Calculated absorption results for **D1-4** DA in cyclohexane at B3LYP/6-31+G(d) level of theory.

Compound	Excitations	Energy (eV)	Wavelength (nm)	Oscillator strength (f)
**D1**	S_1	2.67	464	0.1356
	S_2	2.99	415	0.1027
	S_3	3.11	399	0.0003
	S_4	3.20	388	0.0078
	S_5	3.24	382	0.0152
**D2**	S_1	2.50	495	0.0316
	S_2	2.93	422	0.0021
	S_3	3.01	412	0.0062
	S_4	3.21	386	0.1347
	S_5	3.28	378	0.0360
**D3**	S_1	2.11	587	0.1967
	S_2	2.38	520	0.0001
	S_3	2.44	507	0.0038
	S_4	2.56	483	0.0871
	S_5	2.81	441	0.0564
**D4**	S_1	2.25	551	0.0113
	S_2	2.66	466	0.0008
	S_3	2.75	452	0.0050

	S_4	2.87	433	0.0260
	S_5	3.16	393	0.0046

Table S9: Calculated S1 → S0 emission results for **D1-4** DA compounds in cyclohexane at B3LYP/6-31+G(d) level of theory.

Compound	Energy (eV)	Wavelength (nm)	Oscillator strength (*f*)
**D1**	2.13	583	0.0003
**D2**	1.98	625	0.0002
**D3**	1.26	987	0.0002
**D4**	1.74	713	0.0001

Table S10: Calculated absorption results for **E1-4** DA in cyclohexane at B3LYP/6-31+G(d) level of theory.

Compound	Excitations	Energy (eV)	Wavelength (nm)	Oscillator strength (f)
**E1**	S_1	2.78	446	0.3941
	S_2	3.15	394	0.0662
	S_3	3.23	384	0.0224
	S_4	3.24	382	0.0149
	S_5	3.41	363	0.0098
**E2**	S_1	2.40	517	0.1257
	S_2	2.85	436	0.0259
	S_3	3.12	398	0.0790
	S_4	3.29	377	0.1203
	S_5	3.41	364	0.0437
**E3**	S_1	2.40	517	0.2577
	S_2	2.46	505	0.1239
	S_3	2.80	443	0.0508
	S_4	3.01	411	0.0480
	S_5	3.27	379	0.0268
**E4**	S_1	2.07	598	0.0448
	S_2	2.56	483	0.0271
	S_3	2.74	453	0.0999
	S_4	3.26	381	0.0352
	S_5	3.27	379	0.0378

Table S11: Calculated S1 → S0 emission results for **E1-4** DA compounds in cyclohexane at B3LYP/6-31+G(d) level of theory.

Compound	Energy (eV)	Wavelength (nm)	Oscillator strength (*f*)
**E1**	1.93	644	0.0395
**E2**	1.75	707	0.0368
**E3**	1.02	1217	0.0019
**E4**	1.49	831	0.0230

Table S12: Calculated vertical singlet, triplet excitations, and singlet-triplet energy gaps at ground-state (S_0) geometry of **B1-4** and **C1-4** DA compounds in cyclohexane at B3LYP/6-31+G(d) level of theory.

Compound	Singlet states (eV)		Triplet states (eV)		$\Delta E_{S1T1}^{vert}]^{S0}$ (eV)	$\Delta E_{T1T2}^{vert}]^{S0}$ (eV)
**B1**	S1	2.616	T1	2.178	0.438	0.204
	S2	3.025	T2	2.382		
	S3	3.200	T3	2.596		
**B2**	S1	2.695	T1	2.319	0.376	0.240
	S2	3.146	T2	2.559		
	S3	3.165	T3	2.889		
**B3**	S1	2.056	T1	1.751	0.305	0.156
	S2	2.441	T2	1.907		
	S3	2.468	T3	2.166		
**B4**	S1	2.427	T1	2.261	0.166	0.301
	S2	2.877	T2	2.562		
	S3	2.923	T3	2.721		
**C1**	S1	2.596	T1	2.066	0.530	0.284
	S2	3.095	T2	2.350		
	S3	3.161	T3	2.535		
**C2**	S1	2.619	T1	2.224	0.395	0.256
	S2	3.101	T2	2.480		
	S3	3.235	T3	2.781		
**C3**	S1	2.114	T1	1.674	0.440	0.206
	S2	2.456	T2	1.880		
	S3	2.682	T3	2.219		
**C4**	S1	2.327	T1	2.107	0.220	0.389
	S2	2.886	T2	2.496		
	S3	2.977	T3	2.649		

Table S13: Calculated vertical singlet, triplet excitations, and singlet-triplet energy gaps at ground-state (S_0) geometry of **D1-4** and **E1-4** DA compounds in cyclohexane at B3LYP/6-31+G(d) level of theory.

Compound	Singlet states (eV)		Triplet states (eV)		$\Delta E_{S1T1}^{vert}]^{S0}$ (eV)	$\Delta E_{T1T2}^{vert}]^{S0}$ (eV)
**D1**	S1	2.669	T1	2.230	0.439	0.166
	S2	2.986	T2	2.396		
	S3	3.108	T3	2.638		
**D2**	S1	2.503	T1	2.338	0.165	0.134
	S2	2.935	T2	2.472		
	S3	3.009	T3	2.908		
**D3**	S1	2.113	T1	1.779	0.334	0.118

	S2	2.384	T2	1.897		
	S3	2.445	T3	2.155		
**D4**	S1	2.252	T1	2.211	0.041	0.303
	S2	2.663	T2	2.514		
	S3	2.746	T3	2.699		
**E1**	S1	2.781	T1	2.170	0.611	0.278
	S2	3.151	T2	2.448		
	S3	3.227	T3	2.611		
**E2**	S1	2.398	T1	2.108	0.290	0.344
	S2	2.845	T2	2.452		
	S3	3.115	T3	2.666		
**E3**	S1	2.396	T1	1.883	0.513	0.006
	S2	2.456	T2	1.889		
	S3	2.796	T3	2.320		
**E4**	S1	2.074	T1	1.948	0.126	0.456
	S2	2.566	T2	2.404		
	S3	2.739	T3	2.512		

Table S14: Calculated vertical singlet, triplet excitations, and singlet-triplet energy gaps at singlet excited state (S_1) geometry for **B1-4** and **C1-4** DA compounds in cyclohexane at B3LYP/6-31+G(d) level of theory.

Compound	Singlet states (eV)		Triplet states (eV)		$\Delta E_{S1T1}^{vert}]^{S1}$ (eV)	$\Delta E_{T1T2}^{vert}]^{S1}$ (eV)
**B1**	S1	2.007	T1	1.905	0.102	0.165
			T2	2.070		
			T3	2.595		
**B2**	S1	2.104	T1	2.022	0.082	0.175
			T2	2.197		
			T3	2.728		
**B3**	S1	1.337	T1	1.295	0.042	0.475
			T2	1.770		
			T3	1.858		
**B4**	S1	1.852	T1	1.815	0.037	0.451
			T2	2.266		
			T3	2.493		
**C1**	S1	1.755	T1	1.645	0.110	0.413
			T2	2.058		
			T3	2.592		
**C2**	S1	1.848	T1	1.753	0.095	0.423
			T2	2.176		
			T3	2.536		
**C3**	S1	1.102	T1	1.034	0.068	0.756
			T2	1.790		

			T3	1.861		
**C4**	S1	1.603	T1	1.547	0.056	0.685
			T2	2.232		
			T3	2.383		

Table S15: Calculated vertical singlet, triplet excitations, and singlet-triplet energy gaps at singlet excited state (S_1) geometry for **D1-4** and **E1-4** DA compounds in cyclohexane at B3LYP/6-31+G(d) level of theory.

Compound	Singlet states (eV)		Triplet states (eV)		$\Delta E_{S1T1}^{vert}]^{S1}$ (eV)	$\Delta E_{T1T2}^{vert}]^{S1}$ (eV)
**D1**	S1	2.127	T1	2.001	0.126	0.087
			T2	2.088		
			T3	2.523		
**D2**	S1	1.983	T1	1.976	0.007	0.177
			T2	2.153		
			T3	2.648		
**D3**	S1	1.256	T1	1.239	0.017	0.494
			T2	1.733		
			T3	1.772		
**D4**	S1	1.739	T1	1.736	0.003	0.512
			T2	2.248		
			T3	2.391		
**E1**	S1	1.926	T1	1.826	0.100	0.230
			T2	2.056		
			T3	2.528		
**E2**	S1	1.754	T1	1.677	0.077	0.502
			T2	2.179		
			T3	2.516		
**E3**	S1	1.019	T1	0.996	0.023	0.765
			T2	1.761		
			T3	1.813		
**E4**	S1	1.493	T1	1.448	0.045	0.765
			T2	2.213		
			T3	2.275		

Table S16: Calculated vertical singlet, triplet excitations, and singlet-triplet energy gaps at triplet excited state (T_1) geometry of **B1-4** and **C1-4** DA compounds in cyclohexane at B3LYP/6-31+G(d) level of theory.

Compound	Singlet states (eV)		Triplet states (eV)		$\Delta E_{S1T1}^{vert}]^{T1}$ (eV)	$\Delta E_{T1T2}^{vert}]^{T1}$ (eV)
**B1**	S1	2.30	T1	1.72	0.58	0.49
			T2	2.21		
			T3	2.52		

**B2**	S1	2.39	T1	1.95	0.44	0.37
			T2	2.32		
			T3	2.73		
**B3**	S1	1.78	T1	1.22	0.56	0.54
			T2	1.76		
			T3	2.01		
**B4**	S1	2.12	T1	1.92	0.20	0.44
			T2	2.36		
			T3	2.60		
**C1**	S1	2.21	T1	1.62	0.59	0.63
			T2	2.25		
			T3	2.47		
**C2**	S1	2.24	T1	1.82	0.42	0.51
			T2	2.33		
			T3	2.65		
**C3**	S1	1.62	T1	1.26	0.36	0.58
			T2	1.84		
			T3	1.95		
**C4**	S1	1.92	T1	1.72	0.20	0.61
			T2	2.33		
			T3	2.46		

Table S17: Calculated vertical singlet, triplet excitations, and singlet-triplet energy gaps at triplet excited state (T_1) geometry of **D1-4** and **E1-4** DA compounds in cyclohexane at B3LYP/6-31+G(d) level of theory.

Compound	Singlet states (eV)		Triplet states (eV)		$\Delta E_{S1T1}^{vert}]^{T1}$ (eV)	$\Delta E_{T1T2}^{vert}]^{T1}$ (eV)
**D1**	S1	2.09	T1	1.33	0.76	0.89
			T2	2.22		
			T3	2.42		
**D2**	S1	2.17	T1	1.78	0.39	0.51
			T2	2.29		
			T3	2.69		
**D3**	S1	1.78	T1	1.17	0.61	0.55
			T2	1.72		
			T3	1.99		
**D4**	S1	1.90	T1	1.69	0.21	0.64
			T2	2.33		
			T3	2.48		
**E1**	S1	2.19	T1	1.38	0.81	0.90
			T2	2.28		
			T3	2.46		
**E2**	S1	2.01	T1	1.56	0.45	0.77

			T2	2.33		
			T3	2.53		
**E3**	S1	1.77	T1	1.26	0.51	0.55
			T2	1.81		
			T3	2.00		
**E4**	S1	1.69	T1	1.44	0.25	0.77
			T2	2.21		
			T3	2.31		

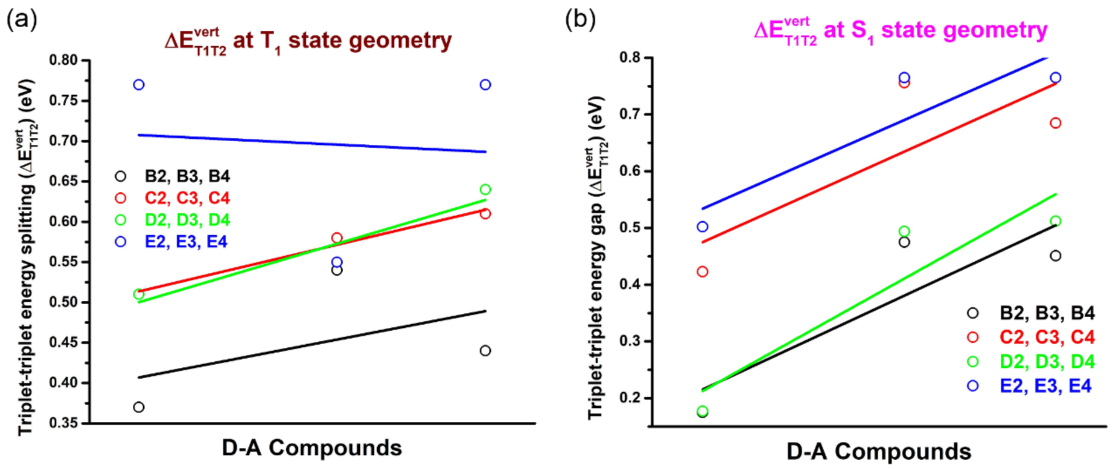

Fig. S5: Linear-fit presentation of calculated vertical triplet-triplet energy splitting gaps (ΔE_{T1T2}^{vert}) at (a) T_1- and (b) S_1-state geometry for studied D-A compounds. Higher nitrogen substituted D-A compounds show a large triplet-triplet gap, which is crucial for efficient RISC process from T_1 to S_1 state.

For validation of the used computational approach in this work i.e. DFT/B3LYP/6-31+G(d), we have performed some computations of experimental results, for example, the results reported by You et al., JACS, 146, 15977, 2024. In **Fig. S6**, we have shown the comparison of our computational and experimental results by You *et al.*. The calculated results are found in perfect trend with the report results, which consequently explains the reliability of the used computational methods for our designed nitrogen substituted PAH based OLED emitters.

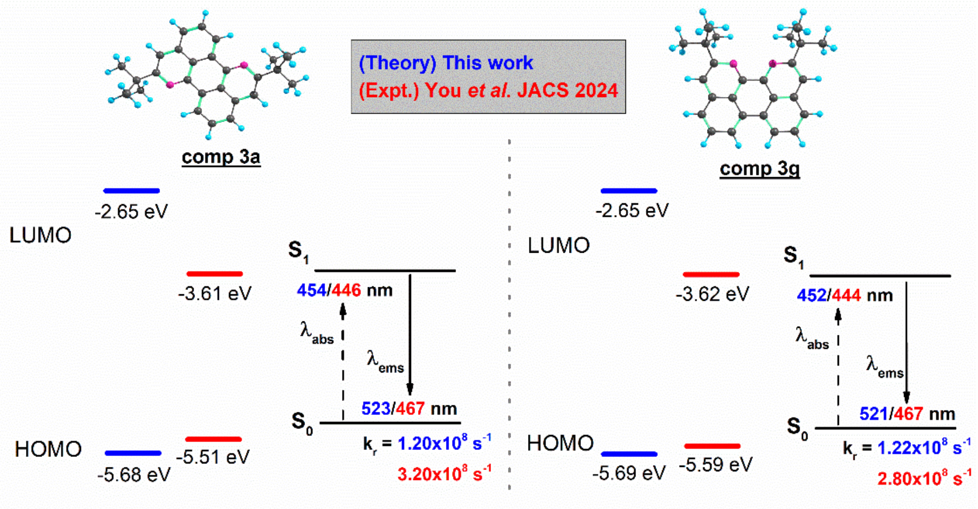

Fig. S6: Computational and experimental results comparison for nitrogen substituted PAH based emitters. (Ref: You et al., JACS, 146, 15977, 2024)

Table S18: Calculated twisting angles (in degree) between donor and acceptor at ground state geometries within the Grimme's empirical dispersion correction (GD3) method for some studied compounds.

Compounds		At ground state (S_0)
Lower nitrogen substituted compounds	**B1**	-38.786/-45.229
	**C1**	-51.034/-46.078
	**D1**	62.222/54.389
	**E1**	-51.912/-32.573
Higher nitrogen substituted compounds	**B4**	-41.595/-38.267
	**C4**	-53.298/-46.940
	**D4**	62.459/59.267
	**E4**	-44.846/-35.153

Table S19: Calculated HOMO/LUMO energies and HOMO-LUMO gaps within the Grimme's empirical dispersion correction (GD3) method for some studied compounds.

Compounds	HOMO (eV)	LUMO (eV)	HOMO-LUMO gap (eV)
**B1**	-5.51	-2.38	3.13
**C1**	-5.28	-2.13	3.15
**D1**	-5.72	-2.39	3.34
**E1**	-5.52	-2.11	3.41
**B4**	-5.35	-2.71	2.64
**C4**	-5.15	-2.52	2.63
**D4**	-5.26	-2.78	2.64
**E4**	-4.99	-2.63	2.36

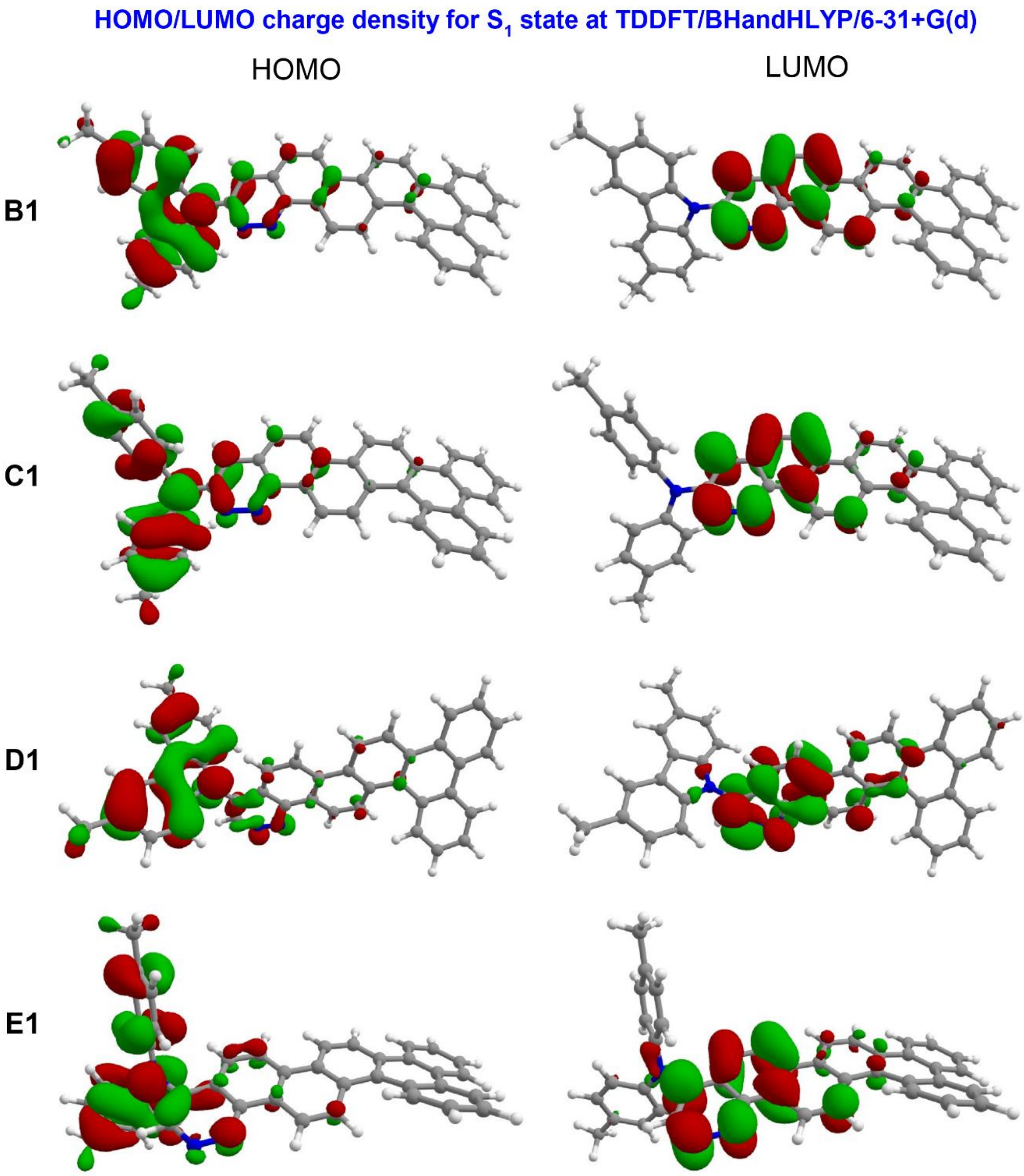

Fig. S7: Calculated HOMO/LUMO charge density distribution plots for singlet excited S1 state of lower number nitrogen substituted D-A compounds **B1**, **C1**, **D1**, and **E1** at TDDFT/BHandHLYP/6-31+G(d) theory level.

Table S20: TDDFT/BHandHLYP/6-31+G(d) theory level calculated photophysical properties of some studied compounds. Here, the TDDFT calculations are performed using the DFT/B3LYP/6-31+G(d) level optimized ground state geometries.

Compounds	Absorption			Emission		
	λ (nm)	E (eV)	*f*	λ (nm)	E (eV)	*f*
**B1**	361	3.44	0.0299	393	3.16	0.0212
**C1**	375	3.31	0.0475	431	2.88	0.0312

**D1**	355	3.50	0.3517	438	2.83	0.0329
**E1**	360	3.44	0.6468	420	2.95	0.4149
**B4**	354	3.51	0.1158	387	3.20	0.0463
**C4**	374	3.32	0.1015	422	2.94	0.0386
**D4**	355	3.49	0.0538	423	2.93	0.0961
**E4**	388	3.20	0.1690	475	2.61	0.1574

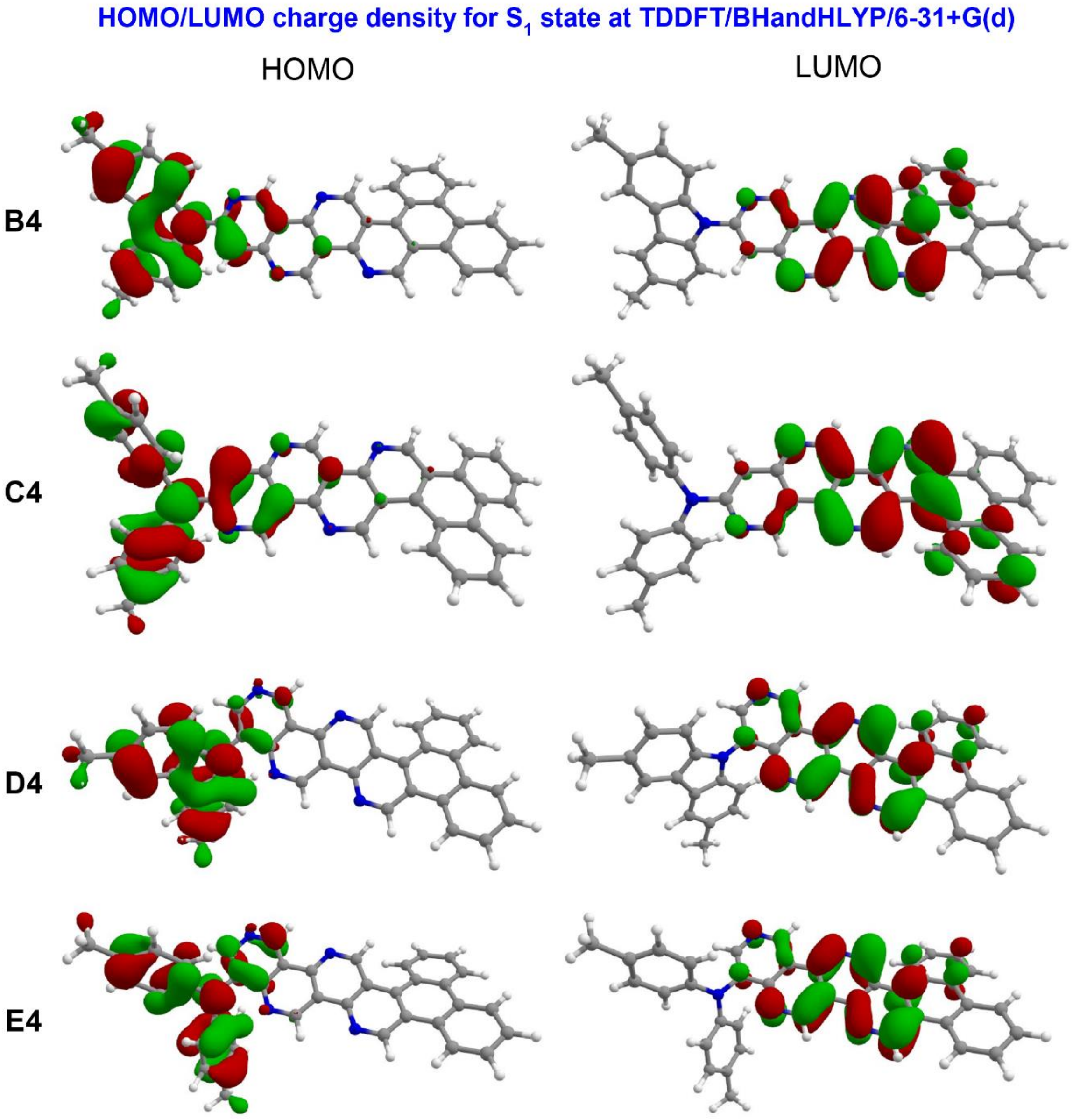

Fig. S8: Calculated HOMO/LUMO charge density distribution plots for singlet excited S_1 state of higher number nitrogen substituted D-A compounds **B4**, **C4**, **D4**, and **E4** at TDDFT/BHandHLYP/6-31+G(d) theory level.

Optimized cartesian coordinates for ground (S_0) state:

S_0 geometry for **B1**

6	2.877146000	-1.244845000	-0.389958000
1	3.466917000	-2.109383000	-0.678185000
6	3.483919000	-0.046463000	-0.031840000
7	2.770577000	1.072335000	0.258552000
7	1.461257000	1.047199000	0.227452000
6	0.791549000	-0.093081000	-0.066794000
6	1.477889000	-1.294586000	-0.398772000
6	0.715728000	-2.456897000	-0.733825000
1	1.237888000	-3.375997000	-0.988495000
6	-0.649847000	-2.413807000	-0.725976000
1	-1.190136000	-3.320330000	-0.973762000
6	-1.383227000	-1.225386000	-0.381592000
6	-0.657064000	-0.058330000	-0.050382000
6	-1.361124000	1.137014000	0.256377000
1	-0.791405000	2.044261000	0.421999000
6	-2.729055000	1.146265000	0.331075000
1	-3.229883000	2.087084000	0.522541000
6	-3.514346000	-0.024737000	0.085913000
6	-2.830990000	-1.191735000	-0.373074000
6	-3.615516000	-2.271775000	-0.861722000
1	-3.142621000	-3.183022000	-1.209276000
6	-4.976199000	-2.159024000	-0.981483000
1	-5.519502000	-2.982297000	-1.430081000
6	-5.684009000	-1.020529000	-0.503510000
6	-4.960268000	-0.020034000	0.176988000
6	-5.708897000	0.959582000	0.969968000
6	-5.117963000	1.656273000	2.053762000
1	-4.095004000	1.437157000	2.333415000
6	-5.830976000	2.569499000	2.813557000
1	-5.346495000	3.076364000	3.643958000
6	-7.181763000	2.811769000	2.523728000
1	-7.751090000	3.529198000	3.108814000
6	-7.804687000	2.086560000	1.521098000
1	-8.867280000	2.233024000	1.364008000
6	-7.105313000	1.134197000	0.745254000
6	-7.800052000	0.270683000	-0.210275000
6	-9.149057000	0.484502000	-0.573997000
1	-9.674788000	1.359430000	-0.208272000
6	-9.826694000	-0.394345000	-1.403156000
1	-10.863054000	-0.201338000	-1.667470000
6	-9.166769000	-1.528687000	-1.900828000
1	-9.687781000	-2.226368000	-2.551160000
6	-7.837022000	-1.746111000	-1.579275000
1	-7.341147000	-2.609681000	-2.007044000

6	-7.118931000	-0.856818000	-0.745069000
6	7.103696000	-0.421537000	0.286521000
6	5.782517000	-0.880317000	0.501387000
7	4.879846000	0.090087000	0.028516000
6	5.628039000	1.177755000	-0.470135000
6	7.004042000	0.882131000	-0.337658000
6	5.545203000	-2.094184000	1.155485000
6	8.190326000	-1.203698000	0.699129000
6	5.201482000	2.375333000	-1.052934000
6	7.963315000	1.798085000	-0.791827000
6	6.646167000	-2.849607000	1.557247000
6	7.974993000	-2.430036000	1.331021000
6	6.178216000	3.263246000	-1.498694000
6	7.560928000	2.998890000	-1.377117000
1	4.149076000	2.618389000	-1.132106000
1	9.022690000	1.571687000	-0.689882000
1	5.860438000	4.201516000	-1.949199000
1	4.540748000	-2.441796000	1.373338000
1	9.206128000	-0.850075000	0.535268000
1	6.471317000	-3.793407000	2.069898000
6	8.573626000	4.008847000	-1.869980000
1	8.485110000	4.961799000	-1.331956000
1	9.597614000	3.644879000	-1.733206000
1	8.437270000	4.227309000	-2.937051000
6	9.132560000	-3.297296000	1.774335000
1	9.059791000	-3.552275000	2.839263000
1	9.162974000	-4.243077000	1.217149000
1	10.091030000	-2.790974000	1.618012000

S_0 geometry for **C1**

6	2.996457000	-1.273987000	-0.292035000
1	3.583396000	-2.167925000	-0.478957000
6	3.608599000	-0.041842000	-0.055887000
7	2.881748000	1.091791000	0.156885000
7	1.573811000	1.055956000	0.144412000
6	0.906143000	-0.103868000	-0.062052000
6	1.599640000	-1.325359000	-0.290860000
6	0.838564000	-2.516191000	-0.518244000
1	1.364019000	-3.452566000	-0.690787000
6	-0.526765000	-2.478775000	-0.512015000
1	-1.063223000	-3.406353000	-0.677168000
6	-1.265435000	-1.267119000	-0.272929000
6	-0.542370000	-0.073801000	-0.046136000
6	-1.251609000	1.140979000	0.156094000
1	-0.684444000	2.061013000	0.241060000
6	-2.619546000	1.152818000	0.231659000

1	-3.122854000	2.105447000	0.341255000
6	-3.401243000	-0.037454000	0.089179000
6	-2.713080000	-1.237517000	-0.265954000
6	-3.494141000	-2.358921000	-0.658084000
1	-3.017051000	-3.294762000	-0.925448000
6	-4.855351000	-2.263120000	-0.786735000
1	-5.395170000	-3.124739000	-1.161673000
6	-5.567663000	-1.090164000	-0.409414000
6	-4.847301000	-0.030771000	0.179451000
6	-5.600329000	1.010543000	0.885270000
6	-5.011814000	1.801581000	1.903721000
1	-3.987128000	1.612942000	2.198581000
6	-5.728483000	2.773423000	2.583160000
1	-5.245179000	3.352771000	3.365519000
6	-7.081033000	2.982904000	2.276345000
1	-7.653356000	3.745271000	2.798376000
6	-7.701359000	2.170547000	1.341204000
1	-8.765023000	2.297074000	1.174041000
6	-6.998010000	1.158399000	0.649070000
6	-7.689708000	0.212328000	-0.227078000
6	-9.040002000	0.387811000	-0.606388000
1	-9.569258000	1.288900000	-0.317036000
6	-9.714437000	-0.562374000	-1.355677000
1	-10.751883000	-0.397590000	-1.634596000
6	-9.049399000	-1.732428000	-1.754061000
1	-9.567511000	-2.486178000	-2.341172000
6	-7.718406000	-1.915182000	-1.416298000
1	-7.218692000	-2.810179000	-1.768296000
6	-7.003424000	-0.953999000	-0.662934000
6	6.911033000	-1.345093000	-0.516368000
6	5.838828000	-0.989529000	0.310765000
7	4.997220000	0.114149000	-0.028553000
6	5.606684000	1.365244000	-0.366504000
6	6.623096000	1.888400000	0.438965000
6	5.621007000	-1.721547000	1.488573000
6	7.745531000	-2.410055000	-0.169525000
6	5.229158000	2.063629000	-1.522003000
6	7.250973000	3.086600000	0.091345000
6	6.451040000	-2.792378000	1.815293000
6	7.531321000	-3.158240000	0.995586000
6	5.851996000	3.265433000	-1.849623000
6	6.875806000	3.802334000	-1.052390000
1	4.442380000	1.668910000	-2.157612000
1	8.040200000	3.474497000	0.732138000
1	5.540259000	3.793389000	-2.748909000
1	4.803855000	-1.443356000	2.148622000
1	8.574862000	-2.666277000	-0.825488000

1	6.265256000	-3.343707000	2.735246000
6	7.532946000	5.115009000	-1.413455000
1	6.849411000	5.959919000	-1.254820000
1	8.426233000	5.295060000	-0.805781000
1	7.834170000	5.137433000	-2.468106000
6	8.426346000	-4.319282000	1.364741000
1	8.852820000	-4.193301000	2.367943000
1	7.872608000	-5.267259000	1.366527000
1	9.257606000	-4.422041000	0.659417000
1	7.094676000	-0.780042000	-1.425678000
1	6.923996000	1.356468000	1.336961000

S_0 geometry for **D1**

6	-3.308988000	-1.032926000	-0.823935000
6	-3.447304000	-2.145785000	-1.643050000
7	-2.405407000	-2.842164000	-2.144345000
7	-1.176353000	-2.468651000	-1.869785000
6	-0.945586000	-1.397976000	-1.069240000
6	-1.996825000	-0.628847000	-0.491366000
6	-1.669349000	0.448437000	0.386777000
1	-2.468151000	1.011046000	0.857921000
6	-0.362376000	0.756850000	0.642818000
1	-0.157815000	1.568765000	1.331357000
6	0.731246000	0.031890000	0.059023000
6	0.437147000	-1.055250000	-0.794851000
6	1.503655000	-1.776149000	-1.397217000
1	1.262161000	-2.552307000	-2.114068000
6	2.807081000	-1.490565000	-1.087245000
1	3.591137000	-2.031564000	-1.602237000
6	3.163232000	-0.450759000	-0.170335000
6	2.112297000	0.379317000	0.324012000
6	2.471900000	1.564711000	1.020755000
1	1.707813000	2.220976000	1.421169000
6	3.783137000	1.948639000	1.130278000
1	3.999478000	2.898732000	1.604282000
6	4.850811000	1.124270000	0.676260000
6	4.540402000	-0.153431000	0.167014000
6	5.625658000	-1.129582000	0.032693000
6	5.381759000	-2.525858000	0.026044000
1	4.370919000	-2.890660000	0.159429000
6	6.409705000	-3.448437000	-0.082691000
1	6.184464000	-4.511625000	-0.073316000
6	7.736841000	-3.004015000	-0.174759000
1	8.552011000	-3.717094000	-0.265158000
6	8.009232000	-1.648231000	-0.091551000
1	9.045741000	-1.330634000	-0.087506000

6	6.980502000	-0.687848000	0.040217000
6	7.281648000	0.725273000	0.272067000
6	8.591364000	1.243702000	0.155036000
1	9.394642000	0.606811000	-0.198182000
6	8.882999000	2.560612000	0.470880000
1	9.899504000	2.931086000	0.367830000
6	7.859873000	3.410609000	0.918774000
1	8.076106000	4.445412000	1.170862000
6	6.563435000	2.932599000	1.016957000
1	5.786310000	3.620167000	1.329860000
6	6.237276000	1.595500000	0.688189000
6	-6.496345000	0.030599000	0.627425000
6	-5.473618000	-0.927531000	0.431629000
7	-4.450459000	-0.353023000	-0.339635000
6	-4.830641000	0.959948000	-0.662553000
6	-6.088211000	1.232447000	-0.072781000
6	-5.553853000	-2.203125000	0.997555000
6	-7.619768000	-0.305313000	1.396341000
6	-4.177533000	1.909918000	-1.453067000
6	-6.682860000	2.487963000	-0.262901000
6	-6.684361000	-2.506939000	1.753320000
6	-7.727550000	-1.576551000	1.963262000
6	-4.796085000	3.146942000	-1.624504000
6	-6.042746000	3.458347000	-1.036165000
1	-3.222192000	1.699282000	-1.923944000
1	-7.649502000	2.705147000	0.186773000
1	-4.300864000	3.897958000	-2.236626000
1	-4.765952000	-2.937655000	0.859883000
1	-8.409886000	0.426033000	1.553202000
1	-6.764455000	-3.496415000	2.198735000
6	-6.664000000	4.820635000	-1.254001000
1	-6.813201000	5.028557000	-2.321365000
1	-7.639304000	4.896107000	-0.761547000
1	-6.027757000	5.621494000	-0.855110000
6	-8.933273000	-1.964800000	2.790636000
1	-9.472697000	-2.809611000	2.342898000
1	-8.644359000	-2.267787000	3.805318000
1	-9.638450000	-1.131727000	2.880819000
1	-4.429759000	-2.509371000	-1.929433000

S_0 geometry for **E1**

6	-3.462549000	0.916545000	-0.333111000
6	-3.663437000	2.206687000	-0.825980000
7	-2.672440000	3.058321000	-1.147975000
7	-1.416825000	2.695770000	-1.006866000
6	-1.119296000	1.470219000	-0.503894000

6	-2.113171000	0.521769000	-0.129489000
6	-1.696934000	-0.716818000	0.448427000
1	-2.443289000	-1.438207000	0.759653000
6	-0.369785000	-0.998179000	0.618743000
1	-0.106778000	-1.950689000	1.064383000
6	0.666701000	-0.090921000	0.217578000
6	0.287307000	1.150200000	-0.340515000
6	1.294810000	2.088027000	-0.697333000
1	0.986264000	3.069239000	-1.038646000
6	2.622464000	1.765021000	-0.602409000
1	3.357365000	2.523810000	-0.840835000
6	3.064691000	0.490709000	-0.122934000
6	2.072315000	-0.402045000	0.382823000
6	2.512191000	-1.555696000	1.086725000
1	1.795103000	-2.270266000	1.473928000
6	3.840793000	-1.753021000	1.360381000
1	4.116873000	-2.609013000	1.964852000
6	4.851759000	-0.894710000	0.844202000
6	4.467585000	0.139962000	-0.033381000
6	5.507956000	0.788178000	-0.837785000
6	5.212518000	1.440829000	-2.060893000
1	4.196650000	1.434400000	-2.435662000
6	6.198507000	2.037604000	-2.829929000
1	5.934225000	2.519675000	-3.767509000
6	7.535048000	1.990500000	-2.407134000
1	8.317426000	2.458874000	-2.998540000
6	7.861497000	1.291508000	-1.256449000
1	8.908004000	1.201780000	-0.987926000
6	6.877220000	0.656578000	-0.465140000
6	7.242521000	-0.198168000	0.664998000
6	8.562919000	-0.259065000	1.165787000
1	9.322849000	0.403360000	0.766484000
6	8.919135000	-1.142033000	2.172087000
1	9.942110000	-1.163286000	2.538572000
6	7.952253000	-2.001869000	2.715931000
1	8.219104000	-2.699676000	3.505300000
6	6.645698000	-1.942784000	2.259369000
1	5.911174000	-2.591345000	2.722501000
6	6.253651000	-1.041289000	1.241495000
6	-5.226871000	-2.272679000	0.161616000
6	-4.633942000	-1.236870000	-0.579708000
7	-4.557342000	0.079715000	-0.032935000
6	-5.727875000	0.662264000	0.549272000
6	-6.995755000	0.468458000	-0.012274000
6	-4.124503000	-1.525438000	-1.851450000
6	-5.306072000	-3.558751000	-0.366136000
6	-5.613203000	1.441986000	1.710949000

6	-8.122624000	1.040747000	0.582928000
6	-4.195037000	-2.825043000	-2.360625000
6	-4.788251000	-3.865570000	-1.636117000
6	-6.742919000	2.021177000	2.283793000
6	-8.022030000	1.830066000	1.735773000
1	-4.635242000	1.592459000	2.159942000
1	-9.097938000	0.877636000	0.129196000
1	-6.628919000	2.624077000	3.182637000
1	-3.671368000	-0.736456000	-2.444661000
1	-5.770548000	-4.344221000	0.227271000
1	-3.788032000	-3.024491000	-3.349646000
6	-9.240984000	2.454354000	2.375470000
1	-9.146484000	3.545958000	2.436205000
1	-10.149179000	2.229515000	1.806546000
1	-9.389199000	2.085887000	3.398851000
6	-4.876870000	-5.268172000	-2.192664000
1	-4.395084000	-5.338844000	-3.173428000
1	-4.390518000	-5.994639000	-1.529082000
1	-5.920463000	-5.587601000	-2.310639000
1	-4.666243000	2.592250000	-0.980976000
1	-5.624974000	-2.063109000	1.150388000
1	-7.101234000	-0.129356000	-0.912714000

S_0 geometry for **B2**

6	2.829476000	-1.066277000	-0.358076000
1	3.336351000	-1.976543000	-0.655197000
6	3.522725000	0.088984000	-0.019813000
7	2.896874000	1.253046000	0.279531000
6	1.574817000	1.277542000	0.269089000
6	0.752274000	0.162134000	-0.017853000
6	1.422140000	-1.047947000	-0.344953000
7	0.746942000	-2.204983000	-0.664313000
6	-0.559991000	-2.165860000	-0.657286000
1	-1.045387000	-3.106217000	-0.906024000
6	-1.367026000	-1.020301000	-0.337024000
6	-0.690699000	0.176315000	-0.009485000
6	-1.465264000	1.336215000	0.274824000
1	-0.977573000	2.292198000	0.432266000
6	-2.832792000	1.273891000	0.334213000
1	-3.382280000	2.190857000	0.508299000
6	-3.559617000	0.064270000	0.094737000
6	-2.813116000	-1.068089000	-0.348553000
6	-3.534828000	-2.190307000	-0.838008000
1	-3.013020000	-3.078912000	-1.173233000
6	-4.898540000	-2.143844000	-0.972818000
1	-5.394979000	-2.996294000	-1.421185000

6	-5.668656000	-1.038853000	-0.513587000
6	-5.004081000	0.002046000	0.166978000
6	-5.808709000	0.952531000	0.939133000
6	-5.266147000	1.687752000	2.022705000
1	-4.239385000	1.516315000	2.321897000
6	-6.031302000	2.576136000	2.760982000
1	-5.583877000	3.113436000	3.593018000
6	-7.387304000	2.752073000	2.448550000
1	-7.997769000	3.448837000	3.016793000
6	-7.961667000	1.986715000	1.446661000
1	-9.027496000	2.081923000	1.273354000
6	-7.208243000	1.058479000	0.692613000
6	-7.848113000	0.151112000	-0.260849000
6	-9.201311000	0.294973000	-0.642648000
1	-9.773545000	1.147175000	-0.293511000
6	-9.825281000	-0.625499000	-1.468725000
1	-10.866496000	-0.486389000	-1.747214000
6	-9.105276000	-1.732269000	-1.944566000
1	-9.584085000	-2.462256000	-2.591760000
6	-7.770599000	-1.881305000	-1.605107000
1	-7.228919000	-2.725342000	-2.015895000
6	-7.106748000	-0.948187000	-0.774048000
1	1.130767000	2.240045000	0.513227000
6	7.104474000	0.766139000	-0.323187000
6	5.751068000	1.151589000	-0.462265000
7	4.929471000	0.112522000	0.019801000
6	5.763179000	-0.921558000	0.485679000
6	7.113464000	-0.547563000	0.286486000
6	5.411260000	2.378371000	-1.042534000
6	8.125529000	1.620466000	-0.761970000
6	7.808772000	2.850052000	-1.340184000
6	6.447576000	3.204508000	-1.472554000
6	5.443980000	-2.126381000	1.122242000
6	8.144996000	-1.402373000	0.696548000
6	7.846479000	-2.619273000	1.311896000
6	6.491372000	-2.954774000	1.522409000
1	4.377907000	2.687237000	-1.137250000
1	6.195838000	4.163371000	-1.921788000
1	9.166689000	1.322915000	-0.654804000
1	9.182552000	-1.112985000	0.542691000
1	4.417948000	-2.415891000	1.320704000
1	6.251607000	-3.893215000	2.018409000
6	8.890318000	3.794119000	-1.817355000
1	8.861976000	4.746865000	-1.272292000
1	9.886611000	3.361374000	-1.676142000
1	8.777577000	4.029368000	-2.883683000
6	8.941997000	-3.565477000	1.751342000

1	8.847326000	-3.825749000	2.813347000
1	8.912446000	-4.505707000	1.184772000
1	9.932696000	-3.122148000	1.604217000

S_0 geometry for **C2**

6	2.938826000	-1.120526000	-0.226554000
1	3.439578000	-2.066314000	-0.395507000
6	3.643026000	0.068798000	-0.027404000
7	3.008694000	1.255519000	0.159051000
6	1.688068000	1.278608000	0.153054000
6	0.862045000	0.143369000	-0.021773000
6	1.535374000	-1.095896000	-0.213907000
7	0.854414000	-2.281104000	-0.402004000
6	-0.451825000	-2.239256000	-0.398675000
1	-0.938699000	-3.201136000	-0.540525000
6	-1.258705000	-1.064189000	-0.209020000
6	-0.579104000	0.160219000	-0.018764000
6	-1.353578000	1.346637000	0.127629000
1	-0.863855000	2.313575000	0.172425000
6	-2.721126000	1.294738000	0.191104000
1	-3.268647000	2.227095000	0.255917000
6	-3.450507000	0.066819000	0.091203000
6	-2.704496000	-1.110209000	-0.216125000
6	-3.428116000	-2.280840000	-0.572222000
1	-2.906622000	-3.202863000	-0.800910000
6	-4.791694000	-2.249753000	-0.712658000
1	-5.288495000	-3.148441000	-1.058884000
6	-5.560949000	-1.097947000	-0.386371000
6	-4.895108000	0.014995000	0.167276000
6	-5.700154000	1.049648000	0.822600000
6	-5.158100000	1.905786000	1.813714000
1	-4.131472000	1.769838000	2.130776000
6	-5.923003000	2.874658000	2.443054000
1	-5.475429000	3.504940000	3.207058000
6	-7.278679000	3.014225000	2.111361000
1	-7.889012000	3.772917000	2.594110000
6	-7.852802000	2.137739000	1.204931000
1	-8.918486000	2.212739000	1.020790000
6	-7.099541000	1.127487000	0.564595000
6	-7.739682000	0.115697000	-0.276963000
6	-9.092279000	0.215405000	-0.674765000
1	-9.663665000	1.103408000	-0.428459000
6	-9.716708000	-0.794512000	-1.388400000
1	-10.757417000	-0.687665000	-1.682738000
6	-8.997321000	-1.950105000	-1.730389000
1	-9.476139000	-2.750367000	-2.288469000

6	-7.663197000	-2.059797000	-1.374023000
1	-7.121876000	-2.946607000	-1.682461000
6	-6.998705000	-1.036721000	-0.656989000
1	1.246367000	2.260726000	0.309922000
6	6.806497000	1.738791000	0.476157000
6	5.751604000	1.306302000	-0.334332000
7	5.042493000	0.107623000	-0.007412000
6	5.799302000	-1.064597000	0.304111000
6	6.819482000	-1.497838000	-0.551152000
6	5.435759000	2.043733000	-1.484543000
6	7.530564000	2.884949000	0.140219000
6	7.218219000	3.638270000	-0.998322000
6	6.155684000	3.193282000	-1.801539000
6	5.554268000	-1.783488000	1.483879000
6	7.576007000	-2.627022000	-0.230359000
6	7.331695000	-3.363237000	0.936519000
6	6.305641000	-2.917816000	1.785522000
1	4.623359000	1.716604000	-2.126088000
1	5.892449000	3.750746000	-2.698732000
1	8.348195000	3.201140000	0.784880000
1	8.366093000	-2.943743000	-0.908097000
1	4.774754000	-1.448544000	2.162484000
1	6.098902000	-3.459544000	2.706613000
6	7.985226000	4.892643000	-1.350531000
1	7.363956000	5.789665000	-1.225652000
1	8.868739000	5.012016000	-0.714287000
1	8.323531000	4.877736000	-2.394206000
6	8.130374000	-4.603073000	1.268295000
1	8.456004000	-4.602715000	2.315637000
1	7.535112000	-5.513354000	1.115182000
1	9.023103000	-4.683596000	0.639027000
1	7.024161000	-0.943444000	-1.462561000
1	7.062634000	1.175070000	1.368647000

S_0 geometry for **D2**

6	-3.339426000	-1.503883000	0.025369000
6	-3.568668000	-2.870824000	-0.012966000
7	-2.588054000	-3.798319000	-0.078132000
6	-1.334508000	-3.372144000	-0.094342000
6	-0.955531000	-2.007447000	-0.063135000
6	-1.994383000	-1.038249000	-0.009659000
7	-1.750006000	0.310541000	0.002855000
6	-0.504214000	0.706254000	-0.030378000
1	-0.368345000	1.784591000	-0.013661000
6	0.646812000	-0.150919000	-0.070361000
6	0.413864000	-1.544001000	-0.091550000

6	1.535596000	-2.416263000	-0.176047000
1	1.389221000	-3.483842000	-0.297297000
6	2.814617000	-1.926844000	-0.129200000
1	3.636014000	-2.623492000	-0.243359000
6	3.100990000	-0.528891000	-0.018307000
6	1.996707000	0.370696000	-0.095986000
6	2.274986000	1.755536000	-0.250152000
1	1.469709000	2.478943000	-0.298627000
6	3.561756000	2.197521000	-0.420320000
1	3.716396000	3.253179000	-0.609336000
6	4.682270000	1.325993000	-0.320805000
6	4.454211000	-0.018041000	0.039587000
6	5.595566000	-0.822917000	0.482501000
6	5.434344000	-1.959599000	1.313707000
1	4.446310000	-2.222758000	1.671081000
6	6.515721000	-2.711840000	1.743208000
1	6.354601000	-3.570162000	2.390086000
6	7.814518000	-2.340969000	1.365252000
1	8.671138000	-2.924584000	1.691758000
6	8.005785000	-1.188794000	0.620015000
1	9.021685000	-0.878040000	0.404519000
6	6.921405000	-0.392514000	0.186637000
6	7.136137000	0.896193000	-0.472937000
6	8.414317000	1.313100000	-0.908816000
1	9.257532000	0.635382000	-0.836505000
6	8.624577000	2.573935000	-1.442739000
1	9.618573000	2.864882000	-1.771876000
6	7.548851000	3.467841000	-1.558818000
1	7.701493000	4.460249000	-1.974465000
6	6.281661000	3.073095000	-1.162393000
1	5.462711000	3.770023000	-1.297093000
6	6.038319000	1.786452000	-0.626408000
1	-0.577773000	-4.151873000	-0.130044000
6	-6.306638000	0.567790000	-0.526734000
6	-5.358766000	-0.395981000	-0.950020000
7	-4.430478000	-0.604134000	0.076581000
6	-4.774257000	0.219607000	1.153293000
6	-5.935016000	0.957663000	0.818240000
6	-5.426058000	-0.970652000	-2.222640000
6	-7.339105000	0.948254000	-1.395870000
6	-7.430234000	0.383018000	-2.669357000
6	-6.464015000	-0.570967000	-3.062191000
6	-4.169306000	0.347747000	2.406277000
6	-6.484715000	1.844567000	1.755507000
6	-5.893038000	1.994405000	3.010727000
6	-4.739516000	1.235497000	3.315435000
1	-4.695719000	-1.703422000	-2.553354000

1	-6.531125000	-1.008970000	-4.056213000
1	-8.071326000	1.688347000	-1.079074000
1	-7.376845000	2.415673000	1.506072000
1	-3.280595000	-0.219281000	2.666201000
1	-4.279437000	1.349352000	4.295212000
6	-8.537566000	0.776696000	-3.622736000
1	-9.160891000	-0.085231000	-3.895051000
1	-9.194126000	1.532481000	-3.178241000
1	-8.137037000	1.192643000	-4.556552000
6	-6.466565000	2.946386000	4.037796000
1	-6.740777000	2.423892000	4.963692000
1	-5.745288000	3.728186000	4.310442000
1	-7.366136000	3.443828000	3.659325000
1	-4.589105000	-3.245639000	0.011143000

S_0 geometry for **E2**

6	-3.494018000	-1.024608000	0.458319000
6	-3.799144000	-2.283750000	0.969660000
7	-2.879569000	-3.212277000	1.301643000
6	-1.599206000	-2.908992000	1.144988000
6	-1.140073000	-1.674635000	0.624568000
6	-2.115375000	-0.708433000	0.253935000
7	-1.780443000	0.496079000	-0.307966000
6	-0.513671000	0.767220000	-0.481503000
1	-0.311387000	1.735516000	-0.932433000
6	0.580150000	-0.089692000	-0.121639000
6	0.255743000	-1.346515000	0.434548000
6	1.318916000	-2.241208000	0.746316000
1	1.101932000	-3.250674000	1.077399000
6	2.627540000	-1.857564000	0.614924000
1	3.400582000	-2.588109000	0.819316000
6	3.005328000	-0.560583000	0.141387000
6	1.961468000	0.291199000	-0.326934000
6	2.329152000	1.469971000	-1.030371000
1	1.572195000	2.157931000	-1.387936000
6	3.640973000	1.723604000	-1.338342000
1	3.863242000	2.596614000	-1.940560000
6	4.702688000	0.903803000	-0.863410000
6	4.389098000	-0.154782000	0.013724000
6	5.478291000	-0.771011000	0.776215000
6	5.247527000	-1.448984000	1.999376000
1	4.245745000	-1.481179000	2.409774000
6	6.279837000	-2.018590000	2.727163000
1	6.065884000	-2.520285000	3.667234000
6	7.598560000	-1.916421000	2.260466000
1	8.417054000	-2.362317000	2.819332000

6	7.860717000	-1.190698000	1.109787000
1	8.893716000	-1.058553000	0.808787000
6	6.828186000	-0.582464000	0.360270000
6	7.123219000	0.301532000	-0.768142000
6	8.423785000	0.422017000	-1.308219000
1	9.221652000	-0.214743000	-0.942726000
6	8.712915000	1.332901000	-2.311104000
1	9.722208000	1.400386000	-2.708415000
6	7.696568000	2.161610000	-2.811164000
1	7.911081000	2.881187000	-3.596809000
6	6.408268000	2.044196000	-2.315819000
1	5.635184000	2.670501000	-2.745541000
6	6.084441000	1.112867000	-1.301009000
1	-0.894787000	-3.678196000	1.450346000
6	-6.958588000	-0.239513000	-0.195002000
6	-5.654695000	-0.569467000	-0.590887000
7	-4.519757000	-0.108577000	0.135094000
6	-4.517474000	1.194020000	0.710767000
6	-4.960645000	2.300423000	-0.031664000
6	-5.483001000	-1.370252000	-1.733106000
6	-8.055844000	-0.695881000	-0.928877000
6	-7.897997000	-1.504088000	-2.061927000
6	-6.587338000	-1.833642000	-2.443966000
6	-4.079746000	1.399763000	2.024408000
6	-4.966554000	3.571419000	0.534951000
6	-4.518278000	3.794493000	1.848296000
6	-4.072190000	2.684656000	2.574494000
1	-4.478945000	-1.624689000	-2.060582000
1	-6.425151000	-2.453227000	-3.324245000
1	-9.057296000	-0.425529000	-0.599767000
1	-5.312484000	4.413198000	-0.062717000
1	-3.742738000	0.555225000	2.619080000
1	-3.723140000	2.817800000	3.596699000
6	-9.087132000	-2.003094000	-2.850957000
1	-9.104673000	-3.099404000	-2.901807000
1	-10.029914000	-1.676197000	-2.399427000
1	-9.067963000	-1.632751000	-3.884419000
6	-4.523195000	5.183280000	2.446002000
1	-4.127182000	5.178505000	3.467240000
1	-3.911216000	5.877643000	1.855422000
1	-5.537544000	5.601671000	2.484973000
1	-4.838748000	-2.565125000	1.117196000
1	-5.290725000	2.158228000	-1.056349000
1	-7.113442000	0.375369000	0.686423000

S_0 geometry for **B3**

6	-2.780701000	1.122016000	-0.331374000
1	-3.282602000	2.037891000	-0.622499000
6	-3.466581000	-0.044191000	-0.003350000
7	-2.823793000	-1.215302000	0.274601000
7	-1.518571000	-1.283125000	0.266952000
6	-0.782566000	-0.172565000	0.008661000
6	-1.384891000	1.065746000	-0.308297000
7	-0.653636000	2.201777000	-0.622031000
7	0.632868000	2.143395000	-0.607378000
6	1.309638000	0.984294000	-0.278310000
6	0.648109000	-0.222025000	0.031462000
6	1.410289000	-1.387333000	0.297455000
1	0.894665000	-2.329474000	0.448428000
6	2.780776000	-1.316131000	0.340348000
1	3.337007000	-2.233243000	0.490320000
6	3.501516000	-0.095508000	0.122857000
6	2.751501000	1.047076000	-0.282418000
6	3.437667000	2.204315000	-0.727832000
1	2.863467000	3.080106000	-1.005929000
6	4.801050000	2.178529000	-0.871870000
1	5.287495000	3.054397000	-1.284591000
6	5.586132000	1.060693000	-0.467134000
6	4.945484000	-0.017171000	0.181263000
6	5.772187000	-0.984073000	0.907860000
6	5.254104000	-1.765116000	1.971096000
1	4.230602000	-1.616540000	2.292899000
6	6.040230000	-2.671066000	2.664784000
1	5.611053000	-3.244261000	3.482315000
6	7.393596000	-2.818268000	2.327410000
1	8.020405000	-3.527818000	2.860959000
6	7.944448000	-2.008836000	1.347112000
1	9.008892000	-2.083550000	1.156097000
6	7.169055000	-1.063228000	0.638471000
6	7.783341000	-0.113256000	-0.289853000
6	9.131841000	-0.227367000	-0.697296000
1	9.718467000	-1.085618000	-0.389290000
6	9.732804000	0.730474000	-1.497524000
1	10.770982000	0.613661000	-1.796870000
6	8.993542000	1.845854000	-1.920665000
1	9.454072000	2.604735000	-2.547534000
6	7.662738000	1.966757000	-1.555867000
1	7.105394000	2.818577000	-1.927356000
6	7.021962000	0.995920000	-0.750151000
6	-7.026531000	-0.778381000	-0.330307000
6	-5.669987000	-1.158082000	-0.439416000

7	-4.863512000	-0.099772000	0.040312000
6	-5.716222000	0.935430000	0.477761000
6	-7.057479000	0.545093000	0.259499000
6	-5.310730000	-2.392624000	-0.988858000
6	-8.034463000	-1.645669000	-0.772856000
6	-7.699631000	-2.882783000	-1.324715000
6	-6.334709000	-3.231621000	-1.424488000
6	-5.418608000	2.149893000	1.104675000
6	-8.103836000	1.394880000	0.639818000
6	-7.827730000	2.623182000	1.243790000
6	-6.480565000	2.974277000	1.475019000
1	-4.400427000	2.452246000	1.324639000
1	-6.258109000	3.920364000	1.964039000
1	-4.274933000	-2.701327000	-1.052443000
1	-9.078957000	-1.353002000	-0.688796000
1	-6.068636000	-4.197368000	-1.849527000
1	-9.135576000	1.093083000	0.472534000
6	-8.939876000	3.564360000	1.650367000
1	-8.862017000	3.845590000	2.708184000
1	-8.912551000	4.493627000	1.066144000
1	-9.923275000	3.107010000	1.498391000
6	-8.766514000	-3.841350000	-1.805301000
1	-9.767940000	-3.413572000	-1.688260000
1	-8.632930000	-4.092383000	-2.865445000
1	-8.740393000	-4.784820000	-1.244565000

S_0 geometry for **C3**

6	2.891293000	1.172889000	0.215002000
1	3.387514000	2.123285000	0.377056000
6	3.588060000	-0.025229000	0.028308000
7	2.937944000	-1.217667000	-0.149848000
7	1.633743000	-1.282140000	-0.150982000
6	0.894817000	-0.156142000	0.008487000
6	1.498691000	1.108598000	0.197214000
7	0.761795000	2.270987000	0.387845000
7	-0.524155000	2.209371000	0.377394000
6	-1.199485000	1.020638000	0.171727000
6	-0.535007000	-0.209240000	-0.009005000
6	-1.296811000	-1.397470000	-0.151424000
1	-0.779217000	-2.349338000	-0.201657000
6	-2.666984000	-1.334218000	-0.202787000
1	-3.221184000	-2.263290000	-0.255889000
6	-3.390306000	-0.098491000	-0.114511000
6	-2.641629000	1.080941000	0.167769000
6	-3.329839000	2.278054000	0.487861000
1	-2.755896000	3.178448000	0.672358000

6	-4.693322000	2.267250000	0.632710000
1	-5.180641000	3.181625000	0.950061000
6	-5.477106000	1.112193000	0.347854000
6	-4.834506000	-0.027987000	-0.180881000
6	-5.660552000	-1.066753000	-0.801942000
6	-5.141122000	-1.955579000	-1.776203000
1	-4.117018000	-1.841551000	-2.109695000
6	-5.925990000	-2.930032000	-2.371550000
1	-5.495259000	-3.586182000	-3.123322000
6	-7.279777000	-3.041750000	-2.022361000
1	-7.905636000	-3.804088000	-2.478742000
6	-7.832029000	-2.133637000	-1.133567000
1	-8.896712000	-2.188334000	-0.936863000
6	-7.057895000	-1.117960000	-0.527842000
6	-7.673937000	-0.075426000	0.293802000
6	-9.022891000	-0.146500000	0.709641000
1	-9.608638000	-1.033008000	0.493606000
6	-9.625407000	0.890086000	1.403545000
1	-10.663857000	0.804900000	1.712616000
6	-8.886867000	2.044284000	1.707008000
1	-9.348217000	2.865037000	2.249790000
6	-7.555732000	2.126609000	1.332608000
1	-6.998925000	3.013160000	1.612393000
6	-6.913276000	1.076525000	0.634683000
6	6.695499000	-1.764181000	-0.497014000
6	5.668478000	-1.307950000	0.334093000
7	4.977788000	-0.092110000	0.014401000
6	5.754303000	1.072025000	-0.289417000
6	6.763337000	1.495295000	0.582775000
6	5.361321000	-2.028319000	1.496148000
6	7.402977000	-2.922844000	-0.168746000
6	7.099116000	-3.661828000	0.981462000
6	6.064322000	-3.190217000	1.805429000
6	5.534899000	1.788538000	-1.475012000
6	7.535409000	2.616703000	0.272192000
6	7.317201000	3.352569000	-0.900307000
6	6.301881000	2.915376000	-1.766248000
1	4.766768000	1.457297000	-2.168683000
1	6.116266000	3.456741000	-2.691839000
1	4.568612000	-1.681705000	2.152320000
1	8.199676000	-3.260284000	-0.828388000
1	5.808288000	-3.737426000	2.710651000
1	8.317161000	2.927448000	0.961965000
6	8.133873000	4.583059000	-1.222078000
1	8.494825000	4.565379000	-2.257545000
1	7.538257000	5.497910000	-1.102778000
1	9.004840000	4.668038000	-0.563884000

6	7.844285000	-4.931955000	1.322867000
1	8.732478000	-5.055438000	0.694141000
1	8.170445000	-4.937470000	2.370190000
1	7.210255000	-5.816783000	1.177250000
1	6.942775000	-1.210983000	-1.398546000
1	6.947466000	0.939987000	1.498017000

S_0 geometry for **D3**

6	-3.285880000	-1.332788000	0.235915000
6	-3.506314000	-2.696411000	0.438289000
7	-2.528970000	-3.616464000	0.568023000
7	-1.264673000	-3.263110000	0.507277000
6	-0.956575000	-1.959091000	0.292581000
6	-1.927051000	-0.939402000	0.137157000
7	-1.599873000	0.368965000	-0.154026000
7	-0.360180000	0.709387000	-0.258917000
6	0.659141000	-0.199550000	-0.064346000
6	0.418678000	-1.561971000	0.201537000
6	1.515030000	-2.454176000	0.316208000
1	1.318007000	-3.514211000	0.431503000
6	2.799768000	-1.973881000	0.260483000
1	3.615090000	-2.685875000	0.296361000
6	3.100689000	-0.582493000	0.083168000
6	2.010012000	0.297512000	-0.175364000
6	2.273214000	1.628717000	-0.583252000
1	1.439804000	2.300905000	-0.749821000
6	3.561945000	2.025046000	-0.833337000
1	3.723446000	3.026037000	-1.215423000
6	4.679117000	1.179581000	-0.575274000
6	4.454232000	-0.072554000	0.036868000
6	5.597132000	-0.777708000	0.622396000
6	5.439467000	-1.731468000	1.658870000
1	4.453740000	-1.917737000	2.067268000
6	6.522489000	-2.389033000	2.219784000
1	6.363811000	-3.105929000	3.021021000
6	7.819514000	-2.100803000	1.770692000
1	8.677337000	-2.612053000	2.199389000
6	8.007687000	-1.114489000	0.816015000
1	9.022662000	-0.852827000	0.539505000
6	6.921600000	-0.415228000	0.242445000
6	7.132985000	0.721170000	-0.655130000
6	8.408860000	1.042398000	-1.171321000
1	9.251492000	0.389186000	-0.974257000
6	8.617422000	2.175307000	-1.940728000
1	9.609702000	2.394273000	-2.326089000
6	7.542128000	3.032278000	-2.221666000

1	7.693167000	3.924611000	-2.823222000
6	6.277029000	2.725128000	-1.748460000
1	5.458049000	3.384092000	-2.011623000
6	6.035131000	1.567626000	-0.971324000
6	-6.427461000	0.375752000	-0.453800000
6	-5.492996000	-0.656740000	-0.692640000
7	-4.358157000	-0.443168000	0.117278000
6	-4.584179000	0.719632000	0.886353000
6	-5.854640000	1.242373000	0.557646000
6	-5.733068000	-1.640217000	-1.656226000
6	-7.628518000	0.405405000	-1.174397000
6	-7.899878000	-0.578370000	-2.127767000
6	-6.937669000	-1.586601000	-2.355672000
6	-3.799616000	1.305659000	1.881529000
6	-6.328899000	2.386496000	1.213866000
6	-5.554051000	2.999565000	2.199216000
6	-4.298239000	2.438219000	2.520796000
1	-2.825577000	0.911938000	2.147637000
1	-3.693108000	2.909347000	3.292540000
1	-5.010423000	-2.421166000	-1.871142000
1	-8.349369000	1.200436000	-0.996186000
1	-7.138809000	-2.347328000	-3.107011000
1	-7.305979000	2.792440000	0.960680000
6	-6.037226000	4.239977000	2.917466000
1	-6.060159000	4.091847000	4.004724000
1	-5.380316000	5.097707000	2.722187000
1	-7.047451000	4.516809000	2.597663000
6	-9.193089000	-0.572427000	-2.912465000
1	-9.810543000	0.294554000	-2.654889000
1	-9.006847000	-0.539831000	-3.993579000
1	-9.787049000	-1.474148000	-2.714139000
1	-4.515253000	-3.086973000	0.522795000

S_0 geometry for **E3**

6	-3.451111000	1.046500000	-0.181511000
6	-3.735089000	2.405363000	-0.410004000
7	-2.811271000	3.361562000	-0.589593000
7	-1.523086000	3.082863000	-0.547607000
6	-1.150547000	1.805575000	-0.287118000
6	-2.060678000	0.740549000	-0.080783000
7	-1.655598000	-0.527153000	0.273188000
7	-0.399315000	-0.799885000	0.384941000
6	0.565314000	0.152018000	0.127277000
6	0.245012000	1.483561000	-0.196304000
6	1.289432000	2.429912000	-0.365244000
1	1.029399000	3.470627000	-0.523772000

6	2.599677000	2.027029000	-0.307585000
1	3.372704000	2.781487000	-0.386526000
6	2.980504000	0.663333000	-0.072709000
6	1.944078000	-0.262799000	0.239298000
6	2.286161000	-1.558578000	0.700069000
1	1.492357000	-2.266554000	0.907192000
6	3.597556000	-1.873765000	0.947581000
1	3.818838000	-2.846947000	1.369710000
6	4.662680000	-0.980515000	0.636243000
6	4.360742000	0.230223000	-0.024156000
6	5.456148000	0.969844000	-0.657011000
6	5.233107000	1.867723000	-1.730783000
1	4.232935000	1.983765000	-2.129649000
6	6.271048000	2.557064000	-2.337024000
1	6.062306000	3.229070000	-3.165308000
6	7.588053000	2.358698000	-1.897446000
1	8.410927000	2.895919000	-2.361570000
6	7.842362000	1.426710000	-0.904306000
1	8.873746000	1.231872000	-0.633176000
6	6.803687000	0.696260000	-0.283586000
6	7.089045000	-0.386511000	0.658606000
6	8.387292000	-0.615096000	1.168547000
1	9.189985000	0.073911000	0.930594000
6	8.668030000	-1.700093000	1.982803000
1	9.675803000	-1.847649000	2.362014000
6	7.644943000	-2.600829000	2.316866000
1	7.852353000	-3.456422000	2.954080000
6	6.358819000	-2.383572000	1.850391000
1	5.580457000	-3.073504000	2.154363000
6	6.043254000	-1.276250000	1.027771000
6	-6.914372000	0.231042000	-0.232491000
6	-5.736528000	0.503967000	0.471379000
7	-4.459770000	0.105410000	-0.045981000
6	-4.343563000	-1.209728000	-0.611181000
6	-4.730578000	-2.328799000	0.133974000
6	-5.817004000	1.153719000	1.712047000
6	-8.150021000	0.608191000	0.298423000
6	-8.246796000	1.270078000	1.529486000
6	-7.054855000	1.536482000	2.223353000
6	-3.894590000	-1.381007000	-1.923577000
6	-4.653067000	-3.600558000	-0.428363000
6	-4.181569000	-3.795425000	-1.736799000
6	-3.806776000	-2.662578000	-2.470673000
1	-3.612706000	-0.515025000	-2.516829000
1	-3.450687000	-2.778431000	-3.492158000
1	-4.907881000	1.353226000	2.272686000
1	-9.054902000	0.388706000	-0.264048000

1	-7.095879000	2.040057000	3.187152000
1	-4.952910000	-4.461012000	0.166539000
6	-4.066224000	-5.184331000	-2.321731000
1	-3.961208000	-5.152011000	-3.411484000
1	-3.188898000	-5.710703000	-1.921966000
1	-4.945726000	-5.794975000	-2.085473000
6	-9.584109000	1.679377000	2.102575000
1	-10.403140000	1.435306000	1.418165000
1	-9.782842000	1.171078000	3.054972000
1	-9.621769000	2.758316000	2.298564000
1	-4.761687000	2.750891000	-0.469771000
1	-5.081381000	-2.199303000	1.153588000
1	-6.863703000	-0.278003000	-1.190363000

S_0 geometry for **B4**

6	-2.790998000	1.089573000	-0.366402000
1	-3.308475000	1.993846000	-0.662954000
6	-3.472247000	-0.074358000	-0.026449000
7	-2.841013000	-1.239561000	0.263118000
6	-1.518563000	-1.258327000	0.248897000
6	-0.716976000	-0.128446000	-0.032408000
6	-1.383956000	1.083071000	-0.359571000
7	-0.711525000	2.242871000	-0.690866000
6	0.596067000	2.211886000	-0.682955000
6	1.384247000	1.060506000	-0.340926000
6	0.719550000	-0.137397000	-0.022677000
7	1.390102000	-1.300084000	0.243223000
6	2.699825000	-1.259424000	0.307174000
1	3.187020000	-2.218482000	0.456001000
6	3.504582000	-0.087075000	0.111865000
6	2.818249000	1.078050000	-0.321160000
7	3.468518000	2.202680000	-0.730818000
6	4.776333000	2.136598000	-0.832263000
1	5.250640000	3.037519000	-1.211259000
6	5.585847000	1.026282000	-0.446874000
6	4.943741000	-0.054849000	0.187274000
6	5.758562000	-1.041660000	0.891511000
6	5.219418000	-1.876752000	1.900849000
1	4.187663000	-1.752520000	2.204633000
6	5.998198000	-2.808012000	2.567330000
1	5.557437000	-3.425666000	3.344870000
6	7.359880000	-2.925741000	2.251527000
1	7.980969000	-3.655323000	2.764209000
6	7.927106000	-2.070264000	1.320887000
1	8.994828000	-2.134987000	1.147115000

6	7.161106000	-1.097448000	0.639630000
6	7.787490000	-0.112883000	-0.245594000
6	9.148809000	-0.195643000	-0.616438000
1	9.743816000	-1.047387000	-0.307259000
6	9.753267000	0.787267000	-1.382767000
1	10.801302000	0.694466000	-1.654260000
6	9.006528000	1.896698000	-1.809079000
1	9.471322000	2.672912000	-2.410780000
6	7.664615000	1.990371000	-1.479613000
1	7.102185000	2.838865000	-1.851593000
6	7.022975000	0.994166000	-0.706432000
1	-1.039484000	-2.206575000	0.477680000
1	1.116114000	3.129807000	-0.944779000
6	-7.065583000	0.544785000	0.284757000
6	-5.717117000	0.928349000	0.477291000
7	-4.878185000	-0.106219000	0.020424000
6	-5.695845000	-1.155797000	-0.447817000
6	-7.050739000	-0.775389000	-0.310644000
6	-5.404379000	2.140560000	1.103120000
6	-8.101513000	1.397127000	0.688477000
6	-7.809317000	2.621560000	1.291856000
6	-6.455961000	2.966516000	1.497446000
6	-5.351657000	-2.387843000	-1.014381000
6	-8.068905000	-1.638937000	-0.737692000
6	-6.385191000	-3.223313000	-1.432992000
6	-7.747695000	-2.873590000	-1.302502000
1	-4.380119000	2.437870000	1.298487000
1	-6.220665000	3.910573000	1.984853000
1	-9.137536000	1.099797000	0.539479000
1	-4.317244000	-2.693523000	-1.105889000
1	-9.111143000	-1.344684000	-0.631860000
1	-6.129633000	-4.186318000	-1.871063000
6	-8.909726000	3.565540000	1.723737000
1	-8.813071000	3.839423000	2.782132000
1	-8.888572000	4.498966000	1.145624000
1	-9.897894000	3.113731000	1.585547000
6	-8.825838000	-3.827908000	-1.766749000
1	-9.823812000	-3.397789000	-1.629271000
1	-8.713511000	-4.075470000	-2.830338000
1	-8.792534000	-4.773941000	-1.210369000

S_0 geometry for **C4**

6	2.899426000	1.140628000	0.239770000
1	3.411002000	2.080541000	0.408277000
6	3.591795000	-0.057714000	0.039120000
7	2.952047000	-1.244987000	-0.135842000

6	1.631201000	-1.261754000	-0.126305000
6	0.825437000	-0.112345000	0.040543000
6	1.496619000	1.128349000	0.233371000
7	0.819017000	2.317602000	0.435423000
6	-0.487834000	2.283857000	0.431948000
6	-1.276598000	1.101502000	0.219176000
6	-0.608526000	-0.123706000	0.034095000
7	-1.280654000	-1.309480000	-0.100880000
6	-2.590062000	-1.276828000	-0.168365000
1	-3.076477000	-2.247031000	-0.210144000
6	-3.396256000	-0.091038000	-0.102699000
6	-2.709222000	1.115482000	0.198106000
7	-3.361908000	2.278632000	0.480168000
6	-4.669345000	2.223491000	0.587739000
1	-5.144412000	3.160780000	0.864093000
6	-5.478500000	1.076891000	0.327919000
6	-4.834971000	-0.067945000	-0.180461000
6	-5.650183000	-1.127259000	-0.770993000
6	-5.110944000	-2.068584000	-1.681905000
1	-4.078863000	-1.978728000	-1.996262000
6	-5.889397000	-3.067809000	-2.242068000
1	-5.448012000	-3.767492000	-2.946388000
6	-7.251170000	-3.150342000	-1.915866000
1	-7.872086000	-3.932122000	-2.345146000
6	-7.818611000	-2.197423000	-1.085386000
1	-8.886449000	-2.242492000	-0.905880000
6	-7.052848000	-1.155265000	-0.515332000
6	-7.679967000	-0.078626000	0.254749000
6	-9.041300000	-0.120131000	0.632467000
1	-9.635636000	-1.001610000	0.420423000
6	-9.646671000	0.941808000	1.284148000
1	-10.694716000	0.879314000	1.564300000
6	-8.900284000	2.092186000	1.583786000
1	-9.365381000	2.930685000	2.094991000
6	-7.558311000	2.148993000	1.246053000
1	-6.996123000	3.033904000	1.520938000
6	-6.915630000	1.073103000	0.589031000
1	1.154943000	-2.228819000	-0.266629000
1	-1.008759000	3.224617000	0.591772000
6	6.772414000	1.489872000	0.569049000
6	5.752965000	1.066672000	-0.291821000
7	4.989752000	-0.104316000	0.010881000
6	5.696349000	-1.310779000	0.316751000
6	6.741503000	-1.737201000	-0.509151000
6	5.515212000	1.792538000	-1.468634000
6	7.535424000	2.617113000	0.256874000
6	7.298618000	3.360353000	-0.907101000

6	6.273409000	2.924486000	-1.762070000
6	5.388793000	-2.060231000	1.461298000
6	7.464460000	-2.890035000	-0.194059000
6	6.107669000	-3.215796000	1.757667000
6	7.160432000	-3.655279000	0.938637000
1	4.735865000	1.465001000	-2.151017000
1	6.072431000	3.472185000	-2.680890000
1	8.324679000	2.926597000	0.938899000
1	4.582884000	-1.738715000	2.113565000
1	8.274295000	-3.201816000	-0.850604000
1	5.850553000	-3.783290000	2.650299000
6	8.104414000	4.597989000	-1.229757000
1	8.436243000	4.599910000	-2.275165000
1	7.511898000	5.510099000	-1.077003000
1	8.993695000	4.672622000	-0.594908000
6	7.925824000	-4.916669000	1.268536000
1	8.800819000	-5.033621000	0.620157000
1	8.277061000	-4.913954000	2.308058000
1	7.298672000	-5.809384000	1.142331000
1	6.990907000	-1.164031000	-1.397514000
1	6.971163000	0.929366000	1.478033000

S_0 geometry for **D4**

6	-3.289462000	-1.481621000	0.133536000
6	-3.495665000	-2.852484000	0.198284000
7	-2.506219000	-3.774502000	0.192192000
6	-1.256820000	-3.336736000	0.138680000
6	-0.910906000	-1.966235000	0.077271000
6	-1.952379000	-0.999059000	0.058687000
7	-1.720551000	0.352165000	-0.040058000
6	-0.478641000	0.758729000	-0.101731000
6	0.663528000	-0.108800000	-0.057216000
6	0.448841000	-1.495506000	0.017831000
7	1.475234000	-2.399209000	-0.010684000
6	2.704705000	-1.941933000	0.001358000
1	3.481116000	-2.695856000	-0.087930000
6	3.074023000	-0.555104000	0.034494000
6	2.013318000	0.378051000	-0.099273000
7	2.225642000	1.710553000	-0.279914000
6	3.467644000	2.102065000	-0.452203000
1	3.588582000	3.164323000	-0.644700000
6	4.622834000	1.269567000	-0.357264000
6	4.429496000	-0.064254000	0.051300000
6	5.587098000	-0.843220000	0.482741000
6	5.455893000	-1.989303000	1.305058000
1	4.479089000	-2.275405000	1.674226000

6	6.558232000	-2.721148000	1.713826000
1	6.425367000	-3.588743000	2.354075000
6	7.842688000	-2.317671000	1.319889000
1	8.715515000	-2.885590000	1.630513000
6	8.002889000	-1.159009000	0.577264000
1	9.009852000	-0.831866000	0.346253000
6	6.897911000	-0.381634000	0.162676000
6	7.078389000	0.906762000	-0.510265000
6	8.345313000	1.352760000	-0.950227000
1	9.208731000	0.703164000	-0.863396000
6	8.519164000	2.609217000	-1.506787000
1	9.505235000	2.922989000	-1.838274000
6	7.418631000	3.469494000	-1.644370000
1	7.544643000	4.456830000	-2.080037000
6	6.161893000	3.048087000	-1.243156000
1	5.323163000	3.717804000	-1.393550000
6	5.958033000	1.765765000	-0.681115000
1	-0.467688000	-4.083218000	0.143869000
1	-0.310508000	1.829412000	-0.184550000
6	-5.921622000	0.992057000	0.748295000
6	-4.753163000	0.293278000	1.136768000
7	-4.395816000	-0.598974000	0.121141000
6	-5.322187000	-0.473473000	-0.920213000
6	-6.283636000	0.505176000	-0.567313000
6	-4.153383000	0.516091000	2.378923000
6	-6.484641000	1.935495000	1.620155000
6	-5.898371000	2.179959000	2.862982000
6	-4.736714000	1.458369000	3.222465000
6	-5.377807000	-1.136319000	-2.149768000
6	-7.317660000	0.811032000	-1.463646000
6	-6.417631000	-0.809170000	-3.017890000
6	-7.396999000	0.157580000	-2.695001000
1	-3.258755000	-0.020699000	2.679708000
1	-4.280599000	1.646438000	4.192617000
1	-7.382548000	2.477248000	1.329346000
1	-4.637208000	-1.881097000	-2.426425000
1	-8.060219000	1.562034000	-1.201208000
1	-6.475689000	-1.316106000	-3.979195000
6	-6.485361000	3.195343000	3.819285000
1	-6.752100000	2.737320000	4.780829000
1	-5.775207000	4.004966000	4.033813000
1	-7.391896000	3.651440000	3.406804000
6	-8.505525000	0.470208000	-3.676640000
1	-9.173569000	1.246022000	-3.287121000
1	-8.106684000	0.826401000	-4.635570000
1	-9.116560000	-0.416521000	-3.891121000
1	-4.511784000	-3.235944000	0.256152000

S_0 geometry for **E4**

6	3.446867000	-1.007263000	-0.468884000
6	3.730233000	-2.264298000	-1.000091000
7	2.801728000	-3.185235000	-1.333830000
6	1.524453000	-2.872446000	-1.163908000
6	1.096824000	-1.634131000	-0.631612000
6	2.075115000	-0.675439000	-0.249247000
7	1.752341000	0.524422000	0.338157000
6	0.488402000	0.806056000	0.520962000
6	-0.595742000	-0.049040000	0.132154000
6	-0.289989000	-1.297732000	-0.435598000
7	-1.257197000	-2.210124000	-0.759952000
6	-2.513538000	-1.855292000	-0.635800000
1	-3.239250000	-2.635675000	-0.844911000
6	-2.972500000	-0.576388000	-0.172107000
6	-1.974117000	0.306302000	0.315743000
7	-2.273304000	1.466854000	0.962902000
6	-3.537917000	1.693366000	1.235534000
1	-3.727714000	2.603281000	1.797941000
6	-4.636793000	0.884898000	0.816670000
6	-4.356719000	-0.195524000	-0.041983000
6	-5.461611000	-0.832430000	-0.754464000
6	-5.255857000	-1.591212000	-1.932888000
1	-4.262020000	-1.665037000	-2.355957000
6	-6.308129000	-2.188998000	-2.606229000
1	-6.118469000	-2.755212000	-3.513857000
6	-7.616474000	-2.033912000	-2.124680000
1	-8.450437000	-2.501288000	-2.641382000
6	-7.851948000	-1.236141000	-1.016619000
1	-8.878213000	-1.075969000	-0.707474000
6	-6.799930000	-0.598087000	-0.321341000
6	-7.064172000	0.344666000	0.768062000
6	-8.357732000	0.523588000	1.308763000
1	-9.177112000	-0.100833000	0.971400000
6	-8.613268000	1.479221000	2.278399000
1	-9.617907000	1.591357000	2.676977000
6	-7.570782000	2.295139000	2.744963000
1	-7.760784000	3.047500000	3.505536000
6	-6.289043000	2.124379000	2.248923000
1	-5.495458000	2.742852000	2.651820000
6	-6.001769000	1.148390000	1.265635000
1	0.785719000	-3.613772000	-1.453369000
1	0.253812000	1.755639000	0.995100000
6	4.957669000	2.293889000	0.035617000
6	4.502718000	1.195734000	-0.712025000

7	4.488706000	-0.109375000	-0.142830000
6	5.612760000	-0.588525000	0.586781000
6	6.923002000	-0.266085000	0.205158000
6	4.067379000	1.412538000	-2.024817000
6	4.977655000	3.567261000	-0.525130000
6	4.531880000	3.801455000	-1.837361000
6	4.074102000	2.700015000	-2.568914000
6	5.425294000	-1.401479000	1.718057000
6	8.010197000	-0.741105000	0.942022000
6	6.519871000	-1.883302000	2.431763000
6	7.836418000	-1.561279000	2.064093000
1	3.721746000	0.574424000	-2.623623000
1	3.726862000	2.841779000	-3.590607000
1	5.331887000	4.402481000	0.076838000
1	4.416700000	-1.650964000	2.035084000
1	9.016574000	-0.475979000	0.623745000
1	6.344972000	-2.511913000	3.303185000
6	4.547144000	5.193757000	-2.426606000
1	4.187743000	5.191968000	-3.461354000
1	3.908125000	5.879395000	-1.854503000
1	5.558548000	5.620681000	-2.426417000
6	9.014870000	-2.080799000	2.855957000
1	9.963922000	-1.753139000	2.418237000
1	8.988752000	-1.726851000	3.895013000
1	9.024873000	-3.177917000	2.889467000
1	4.766504000	-2.552202000	-1.159930000
1	5.285154000	2.143567000	1.059935000
1	7.090607000	0.357546000	-0.667728000

Optimized cartesian coordinates for ground (S_0) state with GD3 correction:

S_0 geometry for **B1**

6	2.879925000	-1.220854000	-0.385541000
1	3.476077000	-2.077985000	-0.682852000
6	3.480366000	-0.026900000	-0.004646000
7	2.764894000	1.086080000	0.303245000
7	1.455998000	1.058609000	0.264732000
6	0.790827000	-0.079036000	-0.052144000
6	1.481412000	-1.274350000	-0.399882000
6	0.725619000	-2.434158000	-0.754351000
1	1.253343000	-3.347253000	-1.018797000
6	-0.639998000	-2.395247000	-0.751769000
1	-1.176331000	-3.300155000	-1.013512000
6	-1.378110000	-1.213731000	-0.394335000
6	-0.657478000	-0.048728000	-0.042524000
6	-1.366482000	1.140233000	0.278780000
1	-0.801430000	2.047479000	0.459315000

6	-2.734206000	1.143155000	0.348706000
1	-3.240861000	2.078650000	0.551981000
6	-3.512763000	-0.027924000	0.084791000
6	-2.825552000	-1.184871000	-0.391733000
6	-3.607002000	-2.257112000	-0.902484000
1	-3.131941000	-3.161274000	-1.265192000
6	-4.967425000	-2.143828000	-1.025719000
1	-5.507641000	-2.959288000	-1.492068000
6	-5.678359000	-1.015494000	-0.528503000
6	-4.957424000	-0.029460000	0.174571000
6	-5.706923000	0.928685000	0.990197000
6	-5.117913000	1.590870000	2.096195000
1	-4.096852000	1.358965000	2.373548000
6	-5.831128000	2.485259000	2.877526000
1	-5.349247000	2.967148000	3.724076000
6	-7.180232000	2.739477000	2.588256000
1	-7.749891000	3.441798000	3.191034000
6	-7.801612000	2.044294000	1.563428000
1	-8.863499000	2.197599000	1.407865000
6	-7.101334000	1.112530000	0.763753000
6	-7.791604000	0.275659000	-0.217789000
6	-9.136055000	0.505054000	-0.588365000
1	-9.659944000	1.375247000	-0.208876000
6	-9.811430000	-0.352035000	-1.441879000
1	-10.844348000	-0.147604000	-1.710897000
6	-9.153329000	-1.479171000	-1.958503000
1	-9.672792000	-2.159760000	-2.627862000
6	-7.827104000	-1.710181000	-1.631739000
1	-7.332117000	-2.566264000	-2.075361000
6	-7.111174000	-0.842691000	-0.773228000
6	7.095993000	-0.422516000	0.287103000
6	5.774058000	-0.867272000	0.524303000
7	4.874957000	0.110129000	0.062518000
6	5.624420000	1.185867000	-0.456666000
6	6.999652000	0.878070000	-0.341800000
6	5.532375000	-2.075994000	1.185924000
6	8.180958000	-1.213240000	0.685328000
6	5.198502000	2.382029000	-1.041204000
6	7.960588000	1.781033000	-0.815487000
6	6.631633000	-2.841492000	1.572419000
6	7.961306000	-2.434562000	1.325043000
6	6.176979000	3.257809000	-1.507301000
6	7.559253000	2.981122000	-1.402787000
1	4.145937000	2.629480000	-1.108225000
1	9.018802000	1.544500000	-0.723742000
1	5.863134000	4.195888000	-1.960608000
1	4.525719000	-2.407840000	1.418592000

1	9.196568000	-0.869546000	0.500539000
1	6.457319000	-3.782328000	2.090570000
6	8.571274000	3.981925000	-1.914555000
1	8.538526000	4.914521000	-1.336034000
1	9.591614000	3.588020000	-1.851629000
1	8.378469000	4.244921000	-2.962478000
6	9.113332000	-3.313051000	1.759197000
1	9.067446000	-3.530863000	2.833956000
1	9.101658000	-4.276683000	1.233091000
1	10.078328000	-2.836143000	1.556589000

S_0 geometry for **C1**

6	2.995002000	-1.266190000	-0.292106000
1	3.584650000	-2.158080000	-0.479972000
6	3.604011000	-0.034524000	-0.047625000
7	2.877563000	1.098511000	0.170483000
7	1.570001000	1.062096000	0.153704000
6	0.903898000	-0.097695000	-0.061273000
6	1.598855000	-1.318289000	-0.294052000
6	0.841137000	-2.509338000	-0.527511000
1	1.369788000	-3.443545000	-0.701861000
6	-0.524092000	-2.473780000	-0.523848000
1	-1.058904000	-3.401621000	-0.692605000
6	-1.264643000	-1.263891000	-0.281901000
6	-0.544183000	-0.069701000	-0.048915000
6	-1.255393000	1.143749000	0.156372000
1	-0.690867000	2.065030000	0.244612000
6	-2.622947000	1.152337000	0.230934000
1	-3.129946000	2.102969000	0.343209000
6	-3.400607000	-0.039812000	0.085060000
6	-2.711832000	-1.236837000	-0.276901000
6	-3.493095000	-2.355362000	-0.677479000
1	-3.016443000	-3.289508000	-0.951371000
6	-4.854188000	-2.258088000	-0.807186000
1	-5.393641000	-3.116701000	-1.189582000
6	-5.566304000	-1.087813000	-0.421041000
6	-4.845144000	-0.035697000	0.178452000
6	-5.595177000	0.995445000	0.899540000
6	-5.004503000	1.763896000	1.933667000
1	-3.981347000	1.563619000	2.227176000
6	-5.717846000	2.727435000	2.627862000
1	-5.234028000	3.290804000	3.421410000
6	-7.069125000	2.948081000	2.321546000
1	-7.639022000	3.703608000	2.855974000
6	-7.691851000	2.154732000	1.371581000
1	-8.755024000	2.288315000	1.206592000

6	-6.991265000	1.152228000	0.662780000
6	-7.682622000	0.222107000	-0.229934000
6	-9.029212000	0.410644000	-0.615665000
1	-9.554271000	1.312276000	-0.320325000
6	-9.705217000	-0.527044000	-1.379236000
1	-10.739801000	-0.352608000	-1.662755000
6	-9.045121000	-1.697033000	-1.786597000
1	-9.564660000	-2.440886000	-2.384911000
6	-7.716938000	-1.891449000	-1.444116000
1	-7.220637000	-2.785019000	-1.804564000
6	-7.000386000	-0.943052000	-0.676344000
6	6.921804000	-1.316391000	-0.489976000
6	5.829415000	-0.983478000	0.321039000
7	4.991395000	0.120255000	-0.016447000
6	5.603902000	1.365055000	-0.361141000
6	6.645432000	1.869625000	0.424029000
6	5.589524000	-1.734867000	1.480288000
6	7.754158000	-2.381447000	-0.144136000
6	5.207906000	2.071290000	-1.504548000
6	7.280711000	3.060310000	0.067134000
6	6.418563000	-2.807734000	1.805861000
6	7.516426000	-3.152680000	1.002700000
6	5.838525000	3.266763000	-1.840981000
6	6.886830000	3.785650000	-1.064833000
1	4.400993000	1.686882000	-2.120674000
1	8.090761000	3.434887000	0.689581000
1	5.514726000	3.803661000	-2.730484000
1	4.755673000	-1.469865000	2.124459000
1	8.601279000	-2.620167000	-0.783912000
1	6.218325000	-3.376017000	2.712050000
6	7.544498000	5.096900000	-1.428621000
1	6.931051000	5.949280000	-1.106169000
1	8.526180000	5.200026000	-0.953190000
1	7.682980000	5.189078000	-2.512524000
6	8.396432000	-4.330961000	1.351141000
1	8.479770000	-4.462226000	2.436113000
1	7.988224000	-5.264593000	0.940245000
1	9.407609000	-4.210575000	0.946836000
1	7.121562000	-0.727991000	-1.380787000
1	6.960300000	1.324378000	1.309115000

S_0 geometry for **D1**

6	-3.312947000	-1.056470000	-0.790278000
6	-3.455283000	-2.190463000	-1.579838000
7	-2.415619000	-2.902965000	-2.061760000
7	-1.184979000	-2.524376000	-1.799402000

6	-0.951762000	-1.434170000	-1.025302000
6	-2.000684000	-0.650939000	-0.463219000
6	-1.674155000	0.439972000	0.397518000
1	-2.473525000	1.008567000	0.859885000
6	-0.367463000	0.753773000	0.645690000
1	-0.162321000	1.575848000	1.321813000
6	0.725051000	0.020386000	0.070275000
6	0.430843000	-1.084553000	-0.761146000
6	1.496800000	-1.816262000	-1.351938000
1	1.255725000	-2.606567000	-2.053245000
6	2.800022000	-1.523046000	-1.050168000
1	3.585099000	-2.072340000	-1.555236000
6	3.154990000	-0.465326000	-0.154021000
6	2.105693000	0.375499000	0.323743000
6	2.468071000	1.577331000	0.990658000
1	1.705675000	2.243988000	1.376852000
6	3.779797000	1.963512000	1.086356000
1	3.997713000	2.925228000	1.535653000
6	4.845554000	1.126991000	0.650142000
6	4.531393000	-0.162697000	0.176202000
6	5.611621000	-1.146053000	0.071096000
6	5.360325000	-2.540169000	0.115043000
1	4.347887000	-2.894335000	0.265972000
6	6.383526000	-3.470466000	0.033192000
1	6.154075000	-4.531702000	0.080065000
6	7.712425000	-3.034859000	-0.079165000
1	8.523975000	-3.754423000	-0.147857000
6	7.991693000	-1.678262000	-0.042895000
1	9.029938000	-1.366367000	-0.051762000
6	6.967539000	-0.709371000	0.058934000
6	7.272482000	0.709576000	0.242844000
6	8.581626000	1.221964000	0.097528000
1	9.380470000	0.573586000	-0.244768000
6	8.878269000	2.547251000	0.371051000
1	9.894185000	2.912839000	0.247165000
6	7.860531000	3.412261000	0.802773000
1	8.080871000	4.453723000	1.021590000
6	6.564080000	2.940068000	0.926304000
1	5.790905000	3.638773000	1.224050000
6	6.232787000	1.594430000	0.640666000
6	-6.511324000	0.064227000	0.578103000
6	-5.514101000	-0.921209000	0.388559000
7	-4.447813000	-0.357878000	-0.329886000
6	-4.774310000	0.975125000	-0.626957000
6	-6.041568000	1.270987000	-0.071386000
6	-5.652098000	-2.208498000	0.913813000
6	-7.670318000	-0.254116000	1.298558000

6	-4.063924000	1.923453000	-1.367452000
6	-6.590025000	2.549177000	-0.242217000
6	-6.817080000	-2.495456000	1.622585000
6	-7.836488000	-1.536906000	1.823818000
6	-4.636692000	3.184556000	-1.521448000
6	-5.892100000	3.518944000	-0.964558000
1	-3.099582000	1.691028000	-1.808063000
1	-7.563991000	2.782019000	0.182801000
1	-4.098564000	3.937664000	-2.093306000
1	-4.881143000	-2.961051000	0.779023000
1	-8.439605000	0.500266000	1.448763000
1	-6.945538000	-3.493076000	2.037250000
6	-6.457458000	4.907800000	-1.163519000
1	-6.564311000	5.146822000	-2.229444000
1	-7.444256000	5.008701000	-0.699071000
1	-5.803102000	5.672372000	-0.724847000
6	-9.080097000	-1.913091000	2.598324000
1	-9.629435000	-2.723630000	2.101968000
1	-8.831473000	-2.262569000	3.608666000
1	-9.762231000	-1.062016000	2.698489000
1	-4.440030000	-2.556218000	-1.854673000

S_0 geometry for **E1**

6	-3.469999000	-0.931561000	0.305606000
6	-3.674942000	-2.232712000	0.766793000
7	-2.685458000	-3.095837000	1.061469000
7	-1.428953000	-2.733110000	0.925526000
6	-1.129426000	-1.495544000	0.452406000
6	-2.121715000	-0.536400000	0.103231000
6	-1.708138000	0.715789000	-0.445515000
1	-2.455632000	1.444252000	-0.736306000
6	-0.381556000	1.001451000	-0.608796000
1	-0.118995000	1.965164000	-1.029739000
6	0.654795000	0.084240000	-0.230228000
6	0.276641000	-1.171733000	0.295814000
6	1.284550000	-2.118418000	0.628152000
1	0.977823000	-3.109121000	0.942492000
6	2.611421000	-1.791127000	0.543632000
1	3.348525000	-2.554095000	0.762215000
6	3.050825000	-0.503127000	0.100544000
6	2.059690000	0.400941000	-0.385712000
6	2.502033000	1.570882000	-1.061054000
1	1.786583000	2.294851000	-1.433445000
6	3.831397000	1.773714000	-1.327182000
1	4.109075000	2.643151000	-1.911479000
6	4.840566000	0.903476000	-0.827502000

6	4.452247000	-0.148209000	0.026792000
6	5.486378000	-0.809218000	0.826472000
6	5.181671000	-1.474309000	2.040362000
1	4.163549000	-1.467486000	2.409748000
6	6.161647000	-2.084595000	2.806114000
1	5.891627000	-2.577357000	3.736406000
6	7.500614000	-2.036349000	2.389916000
1	8.278449000	-2.514434000	2.979496000
6	7.835614000	-1.323589000	1.250009000
1	8.884110000	-1.232776000	0.989598000
6	6.857074000	-0.676309000	0.461575000
6	7.227452000	0.193255000	-0.655037000
6	8.547956000	0.253475000	-1.155300000
1	9.303201000	-0.421679000	-0.768546000
6	8.910134000	1.151631000	-2.145983000
1	9.933041000	1.172219000	-2.512603000
6	7.948944000	2.027561000	-2.674512000
1	8.220659000	2.737221000	-3.451520000
6	6.641721000	1.968581000	-2.219697000
1	5.911478000	2.629101000	-2.672665000
6	6.243619000	1.051869000	-1.218018000
6	-5.199045000	2.277200000	-0.133091000
6	-4.601367000	1.232727000	0.589557000
7	-4.559165000	-0.081233000	0.039341000
6	-5.744451000	-0.638661000	-0.530430000
6	-7.001592000	-0.412378000	0.042775000
6	-4.038189000	1.509687000	1.840875000
6	-5.230288000	3.565740000	0.394253000
6	-5.655029000	-1.423337000	-1.689545000
6	-8.145668000	-0.960520000	-0.540141000
6	-4.060072000	2.810502000	2.348403000
6	-4.658658000	3.861739000	1.642811000
6	-6.802653000	-1.979573000	-2.249995000
6	-8.070885000	-1.758691000	-1.689635000
1	-4.682834000	-1.594427000	-2.143483000
1	-9.113909000	-0.771141000	-0.081519000
1	-6.712439000	-2.586164000	-3.148835000
1	-3.573854000	0.710791000	2.411512000
1	-5.696632000	4.361897000	-0.182903000
1	-3.608490000	3.004396000	3.318963000
6	-9.305408000	-2.384312000	-2.296463000
1	-9.391694000	-3.442060000	-2.013143000
1	-10.217976000	-1.878738000	-1.962613000
1	-9.277959000	-2.343436000	-3.391784000
6	-4.714901000	5.261899000	2.208620000
1	-3.987789000	5.398053000	3.016403000
1	-4.507430000	6.013917000	1.437926000

1	-5.709387000	5.483945000	2.618936000
1	-4.679567000	-2.616052000	0.915049000
1	-5.634984000	2.071023000	-1.106375000
1	-7.081576000	0.196111000	0.938600000

S_0 geometry for **B4**

6	-2.791811000	1.079954000	-0.375162000
1	-3.315557000	1.978525000	-0.678318000
6	-3.468536000	-0.082211000	-0.021718000
7	-2.836858000	-1.244467000	0.278273000
6	-1.514537000	-1.260739000	0.259999000
6	-0.716088000	-0.131108000	-0.032934000
6	-1.385199000	1.077395000	-0.369708000
7	-0.715920000	2.235861000	-0.711330000
6	0.591777000	2.206698000	-0.703864000
6	1.382113000	1.059257000	-0.352724000
6	0.719898000	-0.138115000	-0.024553000
7	1.391685000	-1.298555000	0.249527000
6	2.700950000	-1.254293000	0.313610000
1	3.192291000	-2.210448000	0.468435000
6	3.502079000	-0.080771000	0.111393000
6	2.815696000	1.079961000	-0.332915000
7	3.466473000	2.200830000	-0.752519000
6	4.774286000	2.132426000	-0.854541000
1	5.248649000	3.029605000	-1.242082000
6	5.583116000	1.024690000	-0.458947000
6	4.939743000	-0.046854000	0.189234000
6	5.751113000	-1.022389000	0.910247000
6	5.209622000	-1.831747000	1.938849000
1	4.179523000	-1.693429000	2.243087000
6	5.985015000	-2.753619000	2.621799000
1	5.543616000	-3.352518000	3.413444000
6	7.345437000	-2.884840000	2.304363000
1	7.963997000	-3.606859000	2.830576000
6	7.915281000	-2.051287000	1.355374000
1	8.982602000	-2.124447000	1.182339000
6	7.152334000	-1.089056000	0.655916000
6	7.778996000	-0.123292000	-0.249058000
6	9.137031000	-0.221191000	-0.627794000
1	9.727858000	-1.073249000	-0.311466000
6	9.743505000	0.746784000	-1.411435000
1	10.788961000	0.642715000	-1.688610000
6	9.001794000	1.855671000	-1.848439000
1	9.468338000	2.620017000	-2.463776000
6	7.662378000	1.962914000	-1.512819000
1	7.103318000	2.809305000	-1.894595000

6	7.018772000	0.982035000	-0.722077000
1	-1.032502000	-2.205670000	0.495605000
1	1.110103000	3.123296000	-0.973433000
6	-7.057414000	0.542787000	0.289410000
6	-5.708520000	0.921094000	0.488575000
7	-4.873085000	-0.113419000	0.028001000
6	-5.691184000	-1.156939000	-0.449557000
6	-7.045111000	-0.772816000	-0.313334000
6	-5.392159000	2.127288000	1.123300000
6	-8.092356000	1.394509000	0.694393000
6	-7.796611000	2.614051000	1.305721000
6	-6.442853000	2.954062000	1.518530000
6	-5.347780000	-2.385265000	-1.023636000
6	-8.065438000	-1.628890000	-0.747622000
6	-6.383549000	-3.214316000	-1.449896000
6	-7.745246000	-2.860496000	-1.319490000
1	-4.366336000	2.416125000	1.323504000
1	-6.208302000	3.894652000	2.012821000
1	-9.127774000	1.098656000	0.539153000
1	-4.313144000	-2.690559000	-1.114592000
1	-9.105942000	-1.329274000	-0.641040000
1	-6.132140000	-4.175433000	-1.894322000
6	-8.891987000	3.562972000	1.738409000
1	-8.784105000	3.845022000	2.793525000
1	-8.870849000	4.491116000	1.151842000
1	-9.883222000	3.114033000	1.612268000
6	-8.821782000	-3.811781000	-1.792510000
1	-9.821090000	-3.381861000	-1.663327000
1	-8.699097000	-4.058464000	-2.855124000
1	-8.791903000	-4.757789000	-1.235906000

S_0 geometry for **C4**

6	2.898360000	1.135629000	0.238303000
1	3.414901000	2.072463000	0.408758000
6	3.586656000	-0.063708000	0.031012000
7	2.946438000	-1.249922000	-0.149278000
6	1.625767000	-1.263859000	-0.136833000
6	0.822864000	-0.113026000	0.036393000
6	1.496040000	1.126748000	0.233088000
7	0.821302000	2.316694000	0.439950000
6	-0.485643000	2.284385000	0.437446000
6	-1.276368000	1.103656000	0.221330000
6	-0.610495000	-0.122829000	0.031801000
7	-1.283481000	-1.308222000	-0.105402000
6	-2.592508000	-1.272791000	-0.172486000
1	-3.082741000	-2.241179000	-0.215525000

6	-3.395489000	-0.085057000	-0.105004000
6	-2.708571000	1.120170000	0.200880000
7	-3.361920000	2.281901000	0.488040000
6	-4.669222000	2.224546000	0.598034000
1	-5.144442000	3.160329000	0.878965000
6	-5.477506000	1.077703000	0.334441000
6	-4.832801000	-0.061571000	-0.183863000
6	-5.644828000	-1.114989000	-0.785981000
6	-5.104040000	-2.037247000	-1.714994000
1	-4.074146000	-1.934403000	-2.033410000
6	-5.879344000	-3.032351000	-2.286360000
1	-5.437966000	-3.718339000	-3.003947000
6	-7.239304000	-3.127278000	-1.954531000
1	-7.857845000	-3.905684000	-2.393202000
6	-7.808713000	-2.190115000	-1.107369000
1	-8.875853000	-2.243396000	-0.925818000
6	-7.045774000	-1.153000000	-0.524495000
6	-7.672666000	-0.089116000	0.262683000
6	-9.030102000	-0.143590000	0.652200000
1	-9.620011000	-1.027686000	0.438599000
6	-9.637170000	0.908562000	1.318131000
1	-10.682153000	0.836345000	1.607122000
6	-8.896101000	2.061693000	1.621262000
1	-9.362710000	2.892401000	2.143628000
6	-7.557173000	2.130307000	1.273597000
1	-6.998518000	3.016079000	1.552951000
6	-6.912821000	1.064515000	0.602141000
1	1.146770000	-2.228941000	-0.280488000
1	-1.005043000	3.225340000	0.600742000
6	6.781345000	1.463991000	0.549015000
6	5.744795000	1.058679000	-0.300085000
7	4.983145000	-0.110780000	0.001157000
6	5.691329000	-1.311206000	0.315026000
6	6.761502000	-1.717818000	-0.488854000
6	5.488843000	1.797787000	-1.463283000
6	7.544147000	2.590345000	0.237425000
6	7.288593000	3.349238000	-0.913275000
6	6.247069000	2.930262000	-1.755741000
6	5.364774000	-2.068745000	1.447772000
6	7.491124000	-2.862391000	-0.162820000
6	6.090825000	-3.217337000	1.754588000
6	7.167583000	-3.637926000	0.958151000
1	4.693566000	1.480161000	-2.131598000
1	6.033836000	3.491347000	-2.663427000
1	8.348189000	2.887434000	0.907610000
1	4.539147000	-1.757835000	2.079923000
1	8.322021000	-3.159639000	-0.799474000

1	5.821680000	-3.793610000	2.637808000
6	8.087346000	4.593502000	-1.226144000
1	8.237516000	4.712767000	-2.305494000
1	7.570553000	5.494376000	-0.867917000
1	9.073034000	4.569234000	-0.748420000
6	7.930906000	-4.899499000	1.290463000
1	8.911528000	-4.916609000	0.802032000
1	8.090452000	-4.998992000	2.370883000
1	7.382762000	-5.792442000	0.959942000
1	7.025213000	-1.130705000	-1.363843000
1	6.992483000	0.885854000	1.443957000

S_0 geometry for **D4**

6	-3.290003000	-1.168419000	-0.861786000
6	-3.506900000	-2.327756000	-1.594895000
7	-2.523942000	-3.127153000	-2.065818000
6	-1.270128000	-2.769449000	-1.826893000
6	-0.914981000	-1.612454000	-1.093992000
6	-1.949627000	-0.786096000	-0.577031000
7	-1.709928000	0.337356000	0.177524000
6	-0.465382000	0.662816000	0.413911000
6	0.670684000	-0.080365000	-0.051813000
6	0.447807000	-1.233304000	-0.825157000
7	1.470289000	-1.968695000	-1.359473000
6	2.700006000	-1.639425000	-1.044072000
1	3.477586000	-2.209893000	-1.543517000
6	3.072406000	-0.554824000	-0.180770000
6	2.022700000	0.302663000	0.239115000
7	2.249598000	1.476114000	0.891427000
6	3.499503000	1.861281000	1.014865000
1	3.633609000	2.826622000	1.494543000
6	4.645333000	1.105093000	0.623747000
6	4.427871000	-0.206404000	0.160086000
6	5.560997000	-1.122315000	0.080641000
6	5.387997000	-2.528153000	0.077496000
1	4.395408000	-2.946260000	0.190517000
6	6.467518000	-3.392382000	0.005543000
1	6.303727000	-4.466271000	0.016032000
6	7.769206000	-2.871532000	-0.050022000
1	8.624576000	-3.539021000	-0.109949000
6	7.967855000	-1.502421000	0.028575000
1	8.985950000	-1.132142000	0.056967000
6	6.886078000	-0.597807000	0.120974000
6	7.100268000	0.834951000	0.334745000
6	8.384197000	1.421028000	0.261803000
1	9.237076000	0.822503000	-0.037277000

6	8.588525000	2.759429000	0.554662000
1	9.587135000	3.182344000	0.487335000
6	7.502335000	3.564720000	0.932039000
1	7.652521000	4.615607000	1.163445000
6	6.229527000	3.021486000	0.986390000
1	5.403201000	3.673836000	1.243832000
6	5.994802000	1.659949000	0.682074000
1	-0.486274000	-3.408294000	-2.223297000
1	-0.290691000	1.551047000	1.015464000
6	-6.334268000	0.155580000	0.667191000
6	-5.391729000	-0.879748000	0.454494000
7	-4.385332000	-0.410171000	-0.397534000
6	-4.677623000	0.915529000	-0.735544000
6	-5.880214000	1.299140000	-0.096010000
6	-5.529817000	-2.126770000	1.069929000
6	-7.435224000	-0.073097000	1.503357000
6	-7.598331000	-1.313611000	2.123046000
6	-6.635357000	-2.323203000	1.895505000
6	-3.991209000	1.791828000	-1.578993000
6	-6.391067000	2.589005000	-0.297519000
6	-4.524060000	3.065296000	-1.761655000
6	-5.717666000	3.484593000	-1.129274000
1	-4.801405000	-2.916950000	0.914132000
1	-6.760921000	-3.287849000	2.383316000
1	-8.161720000	0.719318000	1.671354000
1	-3.068163000	1.495849000	-2.066626000
1	-7.314945000	2.889263000	0.192669000
1	-4.001886000	3.764582000	-2.411861000
6	-8.779343000	-1.590133000	3.027347000
1	-9.394626000	-2.414055000	2.642394000
1	-8.453578000	-1.874620000	4.036458000
1	-9.424973000	-0.710197000	3.122275000
6	-6.240899000	4.884755000	-1.363080000
1	-7.185512000	5.052313000	-0.834055000
1	-5.526344000	5.642656000	-1.015456000
1	-6.418432000	5.073727000	-2.429927000
1	-4.526833000	-2.633098000	-1.815846000

S_0 geometry for **E4**

6	-3.456456000	1.026844000	-0.435042000
6	-3.744477000	2.297778000	-0.928949000
7	-2.818198000	3.232829000	-1.227091000
6	-1.540074000	2.919330000	-1.060851000
6	-1.109814000	1.666160000	-0.565593000
6	-2.086435000	0.694155000	-0.215182000
7	-1.766360000	-0.522347000	0.338226000

6	-0.502859000	-0.809873000	0.512110000
6	0.581306000	0.056794000	0.148005000
6	0.276562000	1.324517000	-0.378896000
7	1.243588000	2.247553000	-0.673180000
6	2.499317000	1.887045000	-0.562893000
1	3.227446000	2.672097000	-0.745205000
6	2.956026000	0.591869000	-0.144981000
6	1.959047000	-0.306000000	0.317233000
7	2.260746000	-1.486698000	0.926165000
6	3.526243000	-1.719609000	1.189729000
1	3.717789000	-2.646681000	1.722656000
6	4.623458000	-0.896496000	0.794211000
6	4.339277000	0.206761000	-0.032851000
6	5.438558000	0.861676000	-0.734928000
6	5.224579000	1.640577000	-1.898429000
1	4.228608000	1.715925000	-2.316836000
6	6.271821000	2.256437000	-2.562942000
1	6.077178000	2.838959000	-3.459058000
6	7.582533000	2.097485000	-2.088128000
1	8.412684000	2.578268000	-2.598576000
6	7.825672000	1.278685000	-0.996993000
1	8.854063000	1.115803000	-0.696349000
6	6.778541000	0.623195000	-0.310625000
6	7.047442000	-0.341825000	0.757478000
6	8.341152000	-0.523813000	1.296523000
1	9.156190000	0.115623000	0.977169000
6	8.602306000	-1.501306000	2.242658000
1	9.606901000	-1.615471000	2.640690000
6	7.565151000	-2.336598000	2.686877000
1	7.759759000	-3.106009000	3.428967000
6	6.282729000	-2.162628000	2.193593000
1	5.493001000	-2.795810000	2.580975000
6	5.989768000	-1.164683000	1.234474000
1	-0.802885000	3.671799000	-1.324049000
1	-0.268941000	-1.773182000	0.957797000
6	-4.923803000	-2.298035000	-0.000534000
6	-4.457937000	-1.189357000	-0.723839000
7	-4.490576000	0.110975000	-0.150158000
6	-5.635971000	0.558265000	0.561394000
6	-6.929947000	0.199519000	0.156968000
6	-3.954570000	-1.388801000	-2.014331000
6	-4.886353000	-3.569324000	-0.564700000
6	-4.371596000	-3.787068000	-1.853960000
6	-3.903638000	-2.673537000	-2.560747000
6	-5.485887000	1.371751000	1.696283000
6	-8.040386000	0.642203000	0.877213000
6	-6.604657000	1.822257000	2.393512000

6	-7.904870000	1.466733000	2.002347000
1	-3.594470000	-0.539218000	-2.587840000
1	-3.500959000	-2.804752000	-3.563239000
1	-5.247802000	-4.416171000	0.016191000
1	-4.487930000	1.644613000	2.027294000
1	-9.034306000	0.347810000	0.545782000
1	-6.462559000	2.450817000	3.270668000
6	-4.342153000	-5.175863000	-2.450038000
1	-3.780162000	-5.196808000	-3.390235000
1	-3.875800000	-5.895517000	-1.765204000
1	-5.355906000	-5.541914000	-2.661731000
6	-9.111371000	1.979469000	2.754682000
1	-9.992889000	1.354278000	2.574029000
1	-8.930133000	2.001553000	3.835971000
1	-9.365959000	3.003188000	2.447310000
1	-4.782022000	2.583178000	-1.084282000
1	-5.302819000	-2.155190000	1.006867000
1	-7.061916000	-0.431496000	-0.716670000
